# Reprogramming *Escherichia coli* for the production of prenylated indole diketopiperazine alkaloids

**DOI:** 10.1038/s41598-019-45519-y

**Published:** 2019-06-25

**Authors:** Pavlina Dubois, Isabelle Correia, Fabien Le Chevalier, Steven Dubois, Isabelle Jacques, Nicolas Canu, Mireille Moutiez, Robert Thai, Muriel Gondry, Olivier Lequin, Pascal Belin

**Affiliations:** 1 Institute for Integrative Biology of the Cell (I2BC), CEA, CNRS, Univ. Paris-Sud, Université Paris-Saclay, 91198 Gif-sur-Yvette, cedex France; 2 SIMOPRO, CEA, 91198 Gif-sur-Yvette, cedex France; 3 Sorbonne Université, Ecole Normale Supérieure, PSL University, CNRS, Laboratoire des Biomolécules (LBM), 75005 Paris, France; 4 Present Address: Isabelle B. Jacques, APTEEUS, Institut Pasteur de Lille, Lille, France

**Keywords:** Metabolic engineering, Biocatalysis

## Abstract

Prenylated indole diketopiperazine (DKP) alkaloids are important bioactive molecules or their precursors. In the context of synthetic biology, efficient means for their biological production would increase their chemical diversification and the discovery of novel bioactive compounds. Here, we prove the suitability of the *Escherichia coli* chassis for the production of prenylated indole DKP alkaloids. We used enzyme combinations not found in nature by co-expressing bacterial cyclodipeptide synthases (CDPSs) that assemble the DKP ring and fungal prenyltransferases (PTs) that transfer the allylic moiety from the dimethylallyl diphosphate (DMAPP) to the indole ring of tryptophanyl-containing cyclodipeptides. Of the 11 tested combinations, seven resulted in the production of eight different prenylated indole DKP alkaloids as determined by LC-MS/MS and NMR characterization. Two were previously undescribed. Engineering *E. coli* by introducing a hybrid mevalonate pathway for increasing intracellular DMAPP levels improved prenylated indole DKP alkaloid production. Purified product yields of 2–26 mg/L per culture were obtained from culture supernatants. Our study paves the way for the bioproduction of novel prenylated indole DKP alkaloids in a tractable chassis that can exploit the cyclodipeptide diversity achievable with CDPSs and the numerous described PT activities.

## Introduction

**Figure 1:**
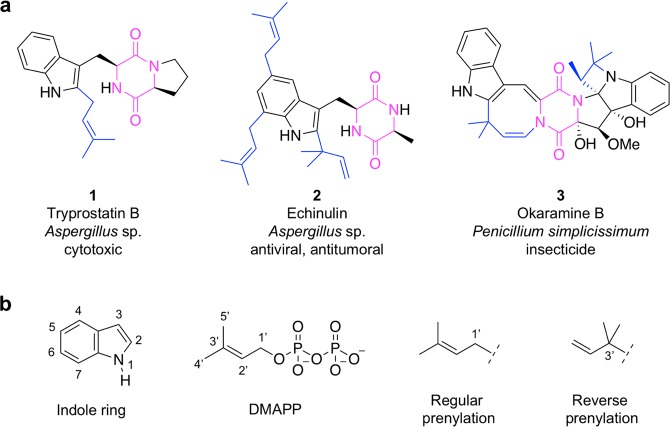
Diversity of indole DKP alkaloid prenylation. (**a**) The chemical structures of diverse prenylated indole DKP alkaloids are shown. The DKP ring is coloured in pink and bonds involving atoms of the prenyl subunits are colored in blue. (**b**) Numbering of the indole ring (N1 to C7) and DMAPP (C1’ to C5’) is indicated. The chemical structures of the allylic moiety resulting from regular prenylation and reverse prenylation are shown. Dashed lines indicate the site of attachment to the indole ring.

Indole diketopiperazine (DKP) alkaloids are natural products mainly isolated from fungi^1,2^. They exhibit various biological activities, including antimicrobial, antiviral, anticancer, and immunomodulatory^1,3^. They are derived from tryptophan, for which condensation with another amino acid results in the formation of a cyclodipeptide (CDP) carrying the 2,5-DKP ring. However, their final chemical structure may be quite complex. Inspection of the natural indole DKP alkaloid chemical structures shows the pervasive presence of a prenyl group on the indole ring. The prenyl group consists of an allylic subunit with at least five carbons (**1**–**3** in Fig. 1a)^1,3^. The prenyl group can be found at the periphery of the molecules and it is proposed to increase lipophilicity to favour interactions with biological membranes and bioactivity^4^. Alternatively, the prenyl group is also found embedded in the molecule, where it provides a carbon skeleton essential for the acquisition of complex polycyclic structures^5^. Prenylated indole DKP alkaloids thus possess specific properties that make them good candidates for lead discovery and drug development.

In the last 15 years, significant progress has been made in the identification and characterization of the enzymes involved in prenylated indole DKP alkaloids biosynthesis in fungi^2,6,7^. The assembly of the DKP ring is performed by non-ribosomal peptide synthetases (NRPSs)^6^. NRPSs are large multidomain modular enzymes specialised in the biosynthesis of a large variety of secondary peptide metabolites^8,9^. They proceed through binding and activation of amino acids, peptide chain elongation and final product release after the introduction of possible modifications, such as cyclisation^2,6^. In the case of prenylated indole DKP alkaloids, a bimodular NRPS assembles a tryptophanyl-containing CDP from tryptophan or its derivatives and another amino acid. Prenylation of the indole moiety is catalysed by prenyltransferases (PTs) belonging to the recently discovered dimethylallyl tryptophan synthase (DMATS) superfamily^2^. These PTs are soluble enzymes that use dimethylallyl diphosphate (DMAPP) to attach the five-carbon allylic moiety to the indole ring. More than 40 DMATS superfamily members have been described^10^. The ease of recombinant expression of these enzymes in *Escherichia coli* and their subsequent purification have allowed their extensive *in vitro* biochemical characterization^11^. Approximately 15 DMATS superfamily PTs are active on Trp-containing CDPs and most have been extensively biochemically characterised *in vitro*^12^. They catalyse prenylation in either a regular (reg) or reverse (rev) mode, depending on the allylic carbon atom involved in attachment to the indole ring (Fig. 1b). Prenylation is generally stereo- and regioselective on the indole ring, but stereo- and regioselectivity may be influenced by the nature of the substrate. These enzymes exhibit varying promiscuity and some of them are highly specific, whereas others exhibit more relaxed specificity. They represent a promising biotechnological tool for the production of highly diverse prenylated indole DKP alkaloids from CDPs^12,13^.

The isolation of prenylated indole DKP alkaloids from natural producers is difficult because of low amounts and the controlled transcription of biosynthetic genes, for which the regulation is often unknown. The chemoenzymatic synthesis of these compounds performed with purified recombinant PTs and chemically synthesised CDPs has allowed an increase in their chemical diversity and production. The biological production of prenylated indole DKP alkaloids using recombinant hosts represents another attractive alternative. In addition to its ecological and economic benefits, it would allow larger scale production of highly diverse prenylated indole DKPs alkaloids. High yields of **1** (250 mg/L) have been obtained in *Aspergillus nidulans* by overexpressing the corresponding NRPS and PT genes from *A. fumigatus*^14^. Recently, combinatorial engineering was implemented in *Aspergillus* using the co-expression of one NRPS gene from *Neosartorya fischeri*, *ftmPS*, with one of three different DMATS superfamily PT genes, *CdpC2PT* from *N. fischeri*, *CdpNPT* from *A. fumigatus*, or *CdpC3PT* from *N. fischeri*^15^. This approach could be used to increase the diversity of prenylated indole DKP alkaloids, provided that NRPSs that synthesise different Trp-containing CDPs can be co-expressed with PTs.

Cyclodipeptide synthases (CDPSs) constitute a recently described family of enzymes that synthesise a large panel of CDPs^16–18^. They originate mainly from bacteria in which several CDPSs have been shown to belong to secondary DKP metabolite biosynthetic pathways^18–22^. CDPSs are small enzymes of approximately 25–30 kDa that use aminoacyl-tRNAs as substrates in a ping-pong mechanism involving the formation of a dipeptidyl-enzyme intermediate that undergoes intramolecular cyclisation, leading to the CDP products^23–25^. The activity of more than 100 CDPSs has thus far been characterised by expression in *E. coli* and identification of the produced CDPs in culture supernatants^17,21,26–32^. Recently, we increased the number of CDPs synthesised by CDPSs in *E. coli* by assessing the incorporation of non-proteinogenic amino acids using the promiscuity of aminoacyl-tRNA synthetases^33^. In total, approximately 300 different CDPs have been produced *in vivo* by CDPSs. Trp-containing CDPs represent an important group of CDPs produced by CDPSs. In the context of prenylated indole DKP alkaloid production, the association of CDPSs and DMATS superfamily PTs in *E. coli*, which produces DMAPP *via* the deoxyxylulose 5-phosphate (DXP) pathway, could be a very powerful means to increase chemical diversity. Here, we are the first to show the production of prenylated indole DKP alkaloids by *E. coli* by combinatorial engineering of bacterial CDPSs and fungal PTs.

## Results

### Expression system for CDPSs and DMATS superfamily PTs

We designed a two-plasmid system for the co-expression of CDPSs and PTs. CDPS genes were cloned in pIJ196 under the control of the T5 promoter followed by two *lacO* operator sequences. Such constructs have proven to be efficient for the soluble expression of active CDPSs in auto-induced minimal medium^29^. We constructed pIJ194 for the expression of PTs. It was derived from pRSFDuet-1 (Novagen) by elimination of the original *Xba*I site and creation of one *Spe*I site and one *Xba*I site upstream and downstream of the PT gene transcription unit, respectively. PT genes were cloned in pIJ194 under the control of the T7 promoter and one *lacO* operator sequence. *E. coli* BL21AI (Thermofisher) was chosen as the expression strain, thus allowing induction of both CDPS and PT gene expression through different means.

We chose 11 DMATS superfamily PTs for which activity on CDPs has been previously characterised *in vitro*. The selected PTs catalyse regular or reverse prenylation at the N1, C2, C3 or C7 atom of the indole ring of various tryptophanyl-containing CDPs. BrePT, EchPT1, and NotF exhibit C2 reverse prenylation on cyclo(l-Trp-l-Pro) (cWP; throughout the text, CDPs containing l-amino acids are annotated cXX, X being one l-amino acid) and share high sequence identity (50–86%)^34–36^. Another group of PTs that catalyse C3 reverse prenylation on cWW is comprised of AnaPT^37^, CdpC3PT^38^, CdpNPT^39,40^, and RoqD^41,42^. They are distinguishable by their capacity to prenylate one or two tryptophanyls of cWW and the stereochemistry of prenylation. CdpC3PT, CdpNPT, and RoqD share 50–69% sequence identity, but display different substrate specificities. AnaPT is poorly related to the three other PTs, sharing only 29–32% sequence identity. The four remaining selected PTs CdpC2PT^43^, CdpC7PT^44^, CTrpPT^45^, and FtmPT1^46^ have singular activities. The tested associations are described in Table 1.

**Table 1:** Studied associations of CDPSs and PTs.

CDPS	Organism	Main produced CDP^†^	Associated PT in this study		
			Name	Organism	Main activity^‡^
CDPS14	*Streptomyces cattleya*	cWW	AnaPT	*Neosartorya fischeri*	reverse prenylation at C3 of cWW (*anti*-*cis*) on one or two Trp
			CdpC2PT	*Neosartorya fischeri*	reverse prenylation at C2 of cWW on one or two Trp
			CdpC7PT	*Aspergillus terreus*	regular prenylation at C7 or reverse prenylation at N1 of cWW
			CdpNPT	*Aspergillus fumigatus*	reverse prenylation at C3 of cWW (*anti*-*cis* and *syn*-*cis*)
			CTrpPT	*Aspergillus orizae*	regular prenylation at C7 and reverse prenylation at N1 of cWW
			RoqD	*Penicillium chrysogenum*	reverse prenylation at C3 of cWW
CDPS68	*Streptomyces* sp. *NRRL F-5053*	cWL	CdpC3PT	*Neosartorya fischeri*	reverse prenylation at C3 of cWL (*syn*-*cis*)
CDPS74	*Streptomyces* sp. *NRRL S-1868*	cWP	BrePT	*Aspergillus versicolor*	reverse prenylation at C2 of cWP
			EchPT1	*Aspergillus ruber*	reverse prenylation at C2 of cWP
			FtmPT1	*Aspergillus fumigatus*	regular prenylation at C2 of cWP
			NotF	*Aspergillus sp*.	reverse prenylation at C2 of cWP

^†^Data for CDPS14^29^ and CDPS68 and CDPS74^28^ were previously published.

^‡^Data for PT activities were taken from published studies: AnaPT^37^, CdpC2PT^43^, CdpC3PT^38^, CdpC7PT^44^, CdpNPT^39,40^, CTrpPT^45^, RoqD^41,42^, BrePT^34^, EchPT1^35^, FtmPT1^46^ and NotF^36^; *anti*-*cis* and *syn*-*cis* refers to the stereochemistry of prenylation at C3.

### Nine of the 11 selected PTs are abundantly produced by *E. coli*

We assessed the efficacy of our expression system under the specific conditions used herein (synthetic genes and co-expression with a CDPS in minimal medium). *E. coli* BL21AI bacteria harbouring each pIJ194-PT plasmid plus the corresponding pIJ196-CDPS plasmid were grown and PT production induced for 48 hours. Analysis of whole cell fractions by SDS-PAGE and Coomassie blue staining showed the presence of a clearly visible additional band between 40 and 55 kDa for all samples (Supplementary Fig. S1a), except CdpC2PT and EchPT1. This is consistent with the expected molecular weight of the recombinant PTs (Supplementary Tables S1 and S2). Peptide mass fingerprinting (PMF) and additional peptide MS/MS sequencing analyses performed on trypsin-digested excised protein bands confirmed the presence of the expected recombinant PTs (Supplementary Table S3). Examination of the soluble fractions by SDS-PAGE revealed an additional band between 40 and 55 kDa for the expression of AnaPT, CdpNPT, CdpC3PT, FtmPT1, and NotF (Supplementary Fig. S1b). Expression of BrePT, CTrpPT, CdpC7PT, and RoqD resulted in the presence of an additional band detected only in the insoluble fractions (Supplementary Fig. S1b,c). Finally, we obtained no evidence of protein production for CdpC2PT or EchPT1 in either the soluble or insoluble fractions. We detected a faint band of approximately 50 kDa in the soluble extract for CdpC2PT, but PMF and peptide MS/MS sequencing did not confirm the presence of CdpC2PT.

### Evidence for prenylated CDP accumulation in culture supernatants upon co-expression of CDPSs and PTs

Bacterial cultures of BL21AI co-expressing CDPS and PT were performed in autoinducing minimal medium (Table 1). We investigated the presence of prenylated CDPs in the culture supernatants as CDPs produced by recombinant CDPSs are recovered in culture supernatants. Culture supernatants were subjected to solid phase extraction and methanol eluates analysed by C18 reverse phase LC-MS/MS.

**Figure 2:**
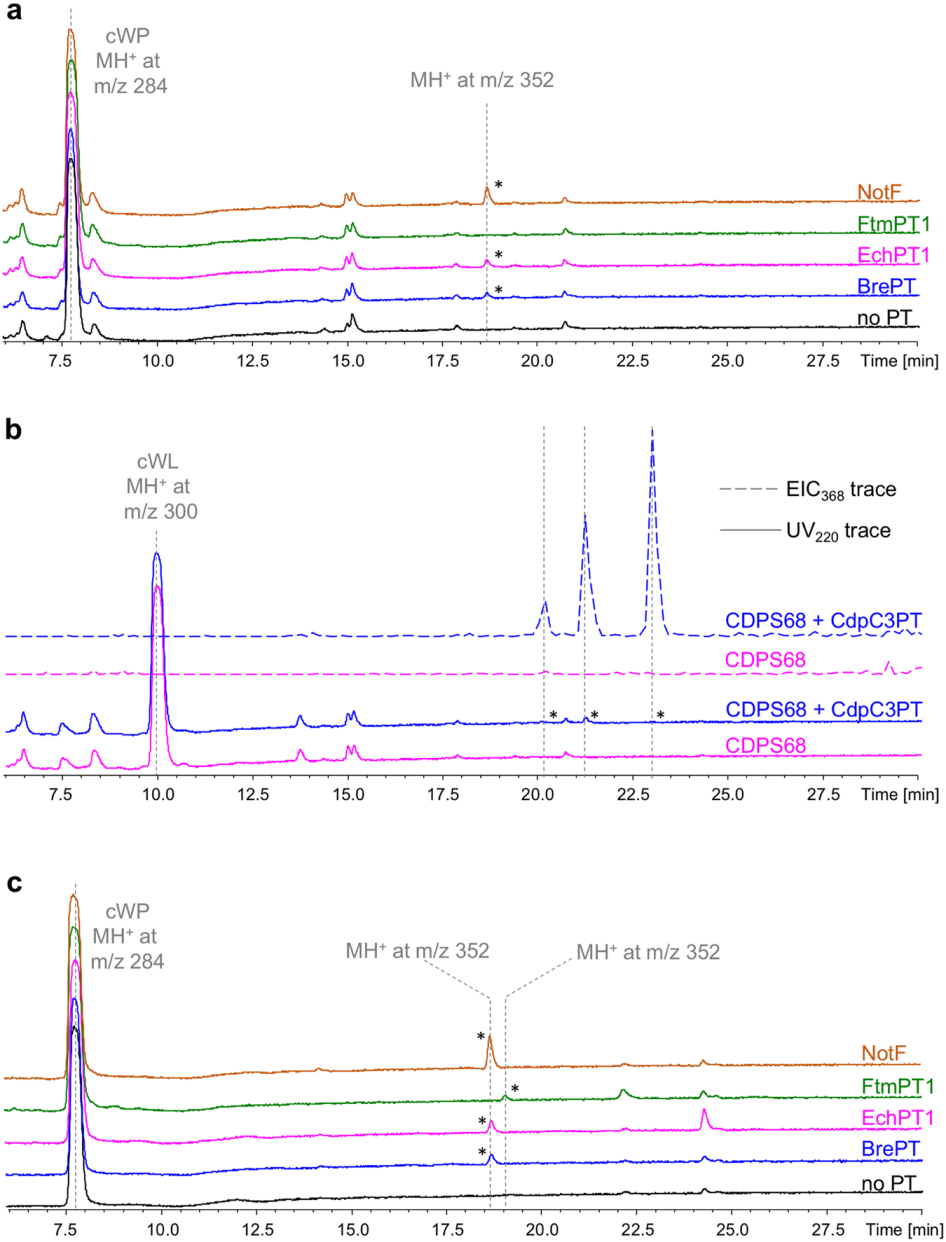
LC-MS/MS analysis of metabolite production by recombinant *E. coli*. Samples corresponding to 50 µl of culture supernatants were analysed. (**a**) SPE-treated bacterial supernatants of cultures of BL21AI expressing CDPS74 and BrePT (blue), EchPT1 (pink), FtmPT1 (green), NotF (orange) or CDPS74 alone (black) were analysed. UV traces recorded at 220 nm are shown between 6 and 30 min with the absorbance scale set from 0 to 700 mU. Asterisks highlight specific peaks for which the MS data are indicated. (**b**) SPE-treated bacterial supernatants of cultures of BL21AI expressing CDPS68 and CdpC3PT (blue) or CDPS68 alone (pink) were analysed. UV chromatograms recorded at 220 nm (UV_220_, plain lines) and extracted ion current at m/z 368 (EIC_368_, dotted lines) are shown between 6 and 30 min. The Y-axis of the UV_220_ traces was set from 0 to 700 mU and that of the EIC_368_ traces from 0 to 3,380,000.

The association of BrePT, EchPT1, or NotF with CDPS74, which synthesises cWP, resulted in the appearance of one additional peak on chromatograms, with a retention time of 18.6 min and MH^+^ ion at m/z 352 (Fig. 2a). The delayed retention time and 68 Da increase in mass with respect to those of cWP (retention time of 7.7 min and MH^+^ ion at m/z 284) are consistent with five-carbon unit prenylation. Furthermore, the same observed retention time for the co-expression of BrePT, EchPT1, or NotF with CDPS74 is coherent with the known identical activity of these three PTs on cWP (Table 1). MSn fragmentation associated with these three additional peaks was similar (Supplementary Figs S2–S4). MS2 spectra showed the presence of an MH^+^ daughter-ion at m/z 284, for which the fragmentation (MS3) corresponds to that of cWP (Supplementary Fig. S5). They also showed the presence of an MH^+^ daughter-ion at m/z 198, which could correspond to the prenylated indole moiety.

We observed three additional compounds for CdpC3PT co-expressed with the cWL-synthesizing CDPS68, characterised by an MH^+^ ion at m/z 368, with retention times of 20.2, 21.2, and 23.0 min (Fig. 2b). Only the compound with a retention time of 21.2 min was visible on UV chromatograms recorded at 220 nm. MSn spectra of the three compounds were highly similar, revealing a MH^+^ daughter-ion at m/z 300, corresponding to cWL, and a MH^+^ daughter-ion at m/z 198 (Supplementary Figs S6–S9).

Concerning the association of PTs with CDPS14, we detected the presence of a compound with a delayed retention time and a 68 Da increase in mass with respect to that of cWW for the co-expression of CDPS14 and CTrpPT (Supplementary Fig. S10). This compound was not visible on UV chromatograms and was detected only on extracted ionic current chromatograms. However, the extracted ionic currents were too weak to obtain MSn fragmentation, preventing us from assessing the cyclodipeptide nature of this compound.

### DMAPP metabolic engineering increases prenylated CDP production

**Figure 3:**
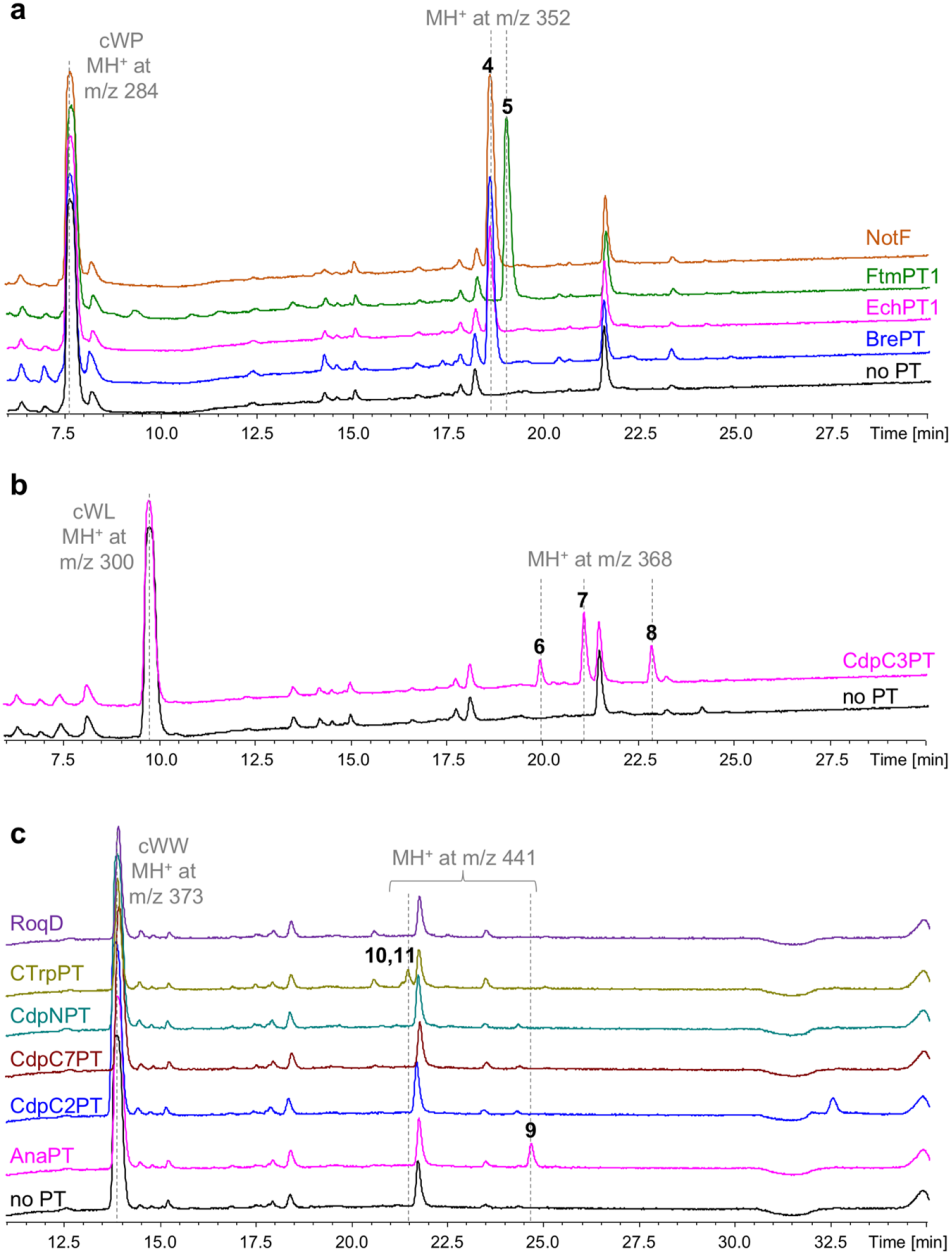
LC-MS/MS analysis of metabolite production by recombinant *E. coli* engineered for DMAPP/IPP production by the mevalonate pathway. SPE-treated culture supernatants of BL21AI bacteria carrying pJBEI-3085 and expressing CDPS74 (**a**), CDPS68 (**b**), or CDPS14 (**c**) plus various PTs, as indicated, were analysed. Each sample corresponded to 50 µl of culture supernatant. UV chromatograms recorded at 220 nm are shown between 6 and 30 min in (**a**,**b**) and between 11 and 35 min in (**c**). The scale of the Y-axis scale of the UV chromatograms was set from 0 to 700 mU. The major CDP synthesised by each CDPS is indicated in each panel with its MS characteristics. Bold Arabic numbers are placed above the peaks for which MS data are indicated.

DMAPP is the prenyl donor necessary for the activity of DMATS superfamily PTs. It is naturally produced in *E. coli via* the DXP pathway. The production of DMAPP and its isomer isopentenyl diphosphate (IPP) in *E. coli* has been the subject of intensive research over the last 15 years to increase their bioavailability for the production of high-value chemicals. We investigated the effects of DMAPP engineering on prenylated CDP production using plasmid pJBEI-3085 which was developed by the laboratory of Taek Soon Lee for terpene production^47^. This plasmid encodes mevalonate-dependent isoprenoid pathways for DMAPP and IPP production in *E. coli* upon IPTG/lactose induction. It is comprised of the MevT and MBI operons, which allow the production of mevalonate from acetyl-CoA (three genes) and the conversion of mevalonate to DMAPP and IPP (four genes), respectively. The two operons are under the control of IPTG/lactose-inducible promoters. Plasmid pJBEI-3085 carries a p15A origin of replication and a chloramphenicol resistance gene. It is thus compatible with the plasmid system we developed for prenylated CDP production. BL21AI bacteria carrying pJBEI-3085 and a CDPS/PT combination (Table 1) were grown as previously in autoinducing minimal medium. Culture supernatants were extracted using solid-phase columns and SPE samples were analysed by reverse-phase LC-MS/MS. The compounds previously detected without DMAPP engineering were recovered, but in much larger amounts (Fig. 3a,b). This increase was particularly significant for one product resulting from the co-expression of CDPS74 and FtmPT1 (**5** in Fig. 3a). Fragmentation spectra suggest that **5** is derived from cWP, given the increase in mass of 68 Da on the indole ring (Supplementary Fig. S11).

Novel additional peaks appeared for the combination of CDPS14 with either AnaPT or CTrpPT (**9**, **10** and **11** in Fig. 3c). They showed delayed retention times, a 68-Da increase in mass and fragmentation patterns indicating that the corresponding compounds were derived from cWW (Supplementary Figs S12–S14). Compounds **10** and **11** were difficult to separate under our HPLC conditions and analysis of the extracted ionic current did not reveal differences in mass spectra. However, their subsequent purification and NMR analysis clearly revealed two isomers (see below). The specific search for compounds with MH^+^ at m/z 441 for CdpC2PT, CdpC7PT, CdpNPT and RoqD was unsuccessful.

Prenylation is described as a chemical modification that favours interactions with biological membranes. We examined whether prenylated CDPs could associate with *E. coli* membranes and thus be less easily recovered from culture supernatants. We thus prepared ethyl-acetate extracts of whole bacterial lysates. Bacterial cultures of BL21AI bacteria carrying pJBEI-3085 and CDPS/PT combinations were performed and induced for prenylated-CDP production as above. Supernatants were treated by SPE to recover extracellular prenylated CDPs and the whole bacterial lysates prepared and extracted with ethyl acetate. Samples were analysed by LC-MS/MS and peak areas on the chromatograms recorded at 220 nm of the CDPs and prenylated CDPs analysed (Supplementary Fig. S15). Globally, we detected no or only very low amounts of prenylated CDPs in whole bacterial ethyl acetate extracts. We did not detect prenylated CDPs for the CDPS/PT associations that inefficiently produced prenylated CDPs in culture supernatants (CDPS14 associated with CdpC2PT, CdpC7PT, CdpNPT or RoqD), even on EIC chromatograms. We obtained similar results using sonication or chemical lysis to prepare the bacterial lysates.

We replaced pJBEI-3085 by pJBEI-3122 in an effort to optimize the production of prenylated CDPs^47^. pJBEI-3122 is derived from pJBEI-3085 by replacing two genes of the MevT operon from *Saccharomyces cerevisiae* by their orthologues from *Staphylococcus aureus*. This plasmid was designed to limit the accumulation of a toxic compound and its use in place of pJBEI-3085 resulted in an increased production of the terpene limonene^47^. However, we did not observe any increase in prenylated-CDP production when using pJBEI-3122 instead of pJBEI-3085.

### Production scale-up and NMR characterisation of prenylated CDPs

We structurally characterised the prenylated CDPs produced in *E. coli* by performing 0.5- to 1-litre cultures of BL21AI bacteria overexpressing one CDPS/PT combination and purifying compounds **4**–**11** from the supernatants. We were able to fully characterise these compounds by ^1^H, ^13^C, and ^15^N NMR in DMSO-*d*_6_ (Table 2 and Supplementary Figs S16–S37).

**Table 2:** Characterisation of the prenylated CDPs produced in *E. coli*.

Compound		Characterization	
#	Enzyme combination	Amount of lyophilised powder (culture volume) and purity of prenylated CDP^†^	NMR structure^‡^
**4**	CDPS74 NotF	17 mg (1 l); 95%	
**5**	CDPS74 FtmPT1	26 mg (1 l); 95%	
**6**	CDPS68 CdpC3PT	RT = 15.1 min§ 2 mg (800 ml); 74%	
**7**	CDPS68 CdpC3PT	RT = 18.5 min^§^ 2.5 mg (800 ml); no contaminant detected	
**8**	CDPS68 CdpC3PT	RT = 26.1 min^§^ 4.5 mg (800 ml); 95%	
**9**	CDPS14 AnaPT	6 mg (800 ml); 88%	
**10, 11**	CDPS14 CTrpPT	3.8 mg (450 ml); 82%	

^†^Amounts were determined by weighing after lyophilisation of the HPLC fractions; purity was evaluated from HPLC chromatograms recorded at 220 nm.

^‡^NMR structures are given based on our data.

^§^When several products were purified from one enzyme combination, the retention time (RT) observed during HPLC purification is indicated for each compound.

Compound **4** was obtained upon co-expression of CDPS74 and NotF. Its ^1^H spectrum showed the characteristic signals of a reverse *tert*-dimethylallyl moiety and was comprised of three vinylic protons and two methyl groups. The absence of an aromatic H2 proton and the observation of long-range scalar ^1^H-^13^C correlations in the HMBC spectrum between quaternary carbon C2 and H1, H4’ and H5’ protons (Supplementary Fig. S16) demonstrated that the prenyl group was attached at the C2 position of the indole ring. The ^1^H NMR data of **4** in DMSO-*d*_6_ (Supplementary Figs S17–S19) are similar to those of cyclo-2-*tert*-prenyl-l-Trp-l-Pro in CD_3_OD obtained upon conversion of cWP by BrePT^34^.

We detected signals of a dimethylallyl moiety in the 1D ^1^H NMR spectrum of **5** (Supplementary Figs S20–S22). Long-range ^13^C-^1^H HMBC connectivity from indole carbon C2 to H1’ and H2’ protons proved that a regular prenylation occurred at position C2 of the indole group (Supplementary Fig. S16). The ^1^H and ^13^C chemical shifts of **5** in DMSO-*d*_6_ are very similar to those of tryprostatin B previously reported for CDCl_3_^48^.

The combination of the cWL-producing CDPS68 and CdpC3PT gave three isolated compounds, **6**, **7** and **8**. Compound **6** (Supplementary Figs S23–S25) was shown to contain a reverse prenyl group attached to carbon C2 of the indole ring, as shown by characteristic signals of a *tert*-dimethylallyl moiety. It also contained HMBC correlations between C3’ and H1 and between C2 and H4’ and H5’ protons (Supplementary Fig. S16). Regular prenylation at position N1 of **7** was inferred from characteristic signals corresponding to a dimethylallyl group, disappearance of the indole HN signal, and the observation of HMBC connectivity between H1’ protons of the prenyl group and C2 and C8 carbons of the indole ring (Supplementary Figs S16 and S26–S28). The NMR analysis of compound **8** (Supplementary Figs S29–S31) revealed the presence of a *tert*-dimethylallyl moiety attached to position C3 of an indoline group, forming a fused five-membered cyclic structure with the DKP ring. The stereochemistry of **8** was determined from the analysis of through-space dipolar correlations in a ^1^H-^1^H ROESY experiment (Supplementary Figs S16 and S31). The strong ROE correlation between H11 and H14 protons of the DKP ring proved the *syn* orientation of substituents on C11 and C14. The observation of several ROEs between prenyl protons, the H2 indoline proton, and one H10 proton (stereospecifically assigned as *anti* to H11) indicate that these protons lie on the face opposite of the H11 hydrogen. Among these three compounds, only **8** has been previously described^38^.

Compound **9** resulted from the co-expression of CDPS14, which synthesises cWW, and AnaPT. NMR analysis (Supplementary Figs S32–S34) proved that reverse prenylation occurred at the C3 position on the indoline ring, which is fused *via* a five-membered ring to the DKP ring. ROE correlations established that the stereochemistry in the indoline ring is opposite to that of **8**, as H2 and the prenyl group lie on the same face as H11 (Supplementary Figs S16 and S34). Accordingly, our NMR data showed that **9** corresponds to the mono-prenylated product obtained upon *in vitro* conversion of cWW by AnaPT^37^.

Compounds **10** and **11** were obtained upon co-expression of CDPS14 and CTrpPT. Our purification conditions did not allow us to separate the two compounds and a mixture of both compounds was analysed by NMR (Supplementary Figs S35–S37). Indeed, the ^1^H NMR spectrum revealed the presence of two sets of signals corresponding to different prenylated derivatives of cWW, with 78% and 22% proportions. The predominant compound (**10**) contains a regular dimethylallyl moiety attached at position C7 of the indole ring. The minor compound (**11**) is characterised by the presence of a reverse dimethylallyl moiety linked to an indole N1 atom. Compounds **10** and **11** correspond to the previously described products obtained *in vitro* after conversion of cWW by CTrpPT^45^.

## Discussion

Prenylated indole DKP alkaloids isolated from nature have revealed many biological activities, making them attractive molecules for the development of novel therapeutics. Herein, we show, for the first time, the possibility to produce prenylated indole DKP alkaloids using the widely-used *E. coli* chassis. In commonly known biosynthetic pathways, NRPSs synthesise CDPs which are further prenylated by PTs. Instead, we associated a CDPS from bacteria and a DMATS superfamily PT from fungi for the production of prenylated indole DKP alkaloids. Among the 11 unprecedented associations tested here, eight efficiently produced indole DKP alkaloids, as shown by mass spectrometry and NMR characterisation. These eight active associations involve three CDPSs that essentially synthesise cWP, cWL, and cWW, and eight DMATS superfamily PTs that catalyse reverse and regular N1 prenylations, reverse and regular C2 prenylations, reverse C3 prenylation, and regular C7 prenylation (Table 2).

The importance of engineering the DMAPP pathway in *E. coli* is a significant issue highlighted by our data. DMAPP is naturally produced by the DXP pathway in *E. coli*, but its bioavailability has proven to be a bottleneck for the recombinant production of numerous DMAPP-derived compounds^49^. We used plasmid pJBEI-3085, encoding seven proteins from bacteria and yeast, to introduce the mevalonate-dependent production of DMAPP in *E. coli*^47^. Without such DMAPP pathway engineering, only five CDPS/PT associations led to prenylated indole DKP alkaloids production; with DMAPP pathway engineering, nine CDPS/PT associations were effective and the levels of prenylated indole DKP alkaloids for four of these associations were clearly above those obtained without DMAPP pathway engineering.

The association of the cWP-synthesizing CDPS74 with PTs was the most efficient for high-level production of prenylated compounds, as shown by the peak areas of UV chromatograms (Figs 2 and 3) and the final amounts of purified compounds (Table 2). Each association led to a single detected compound. The structural characterisation of the products of the CDPS74/FtmPT1 and CDPS74/NotF associations are consistent with the major activities of FtmPT1 and NotF on cWP, consisting of regular prenylation and reverse prenylation at C2, respectively. We also observed the products of the CDPS74/EchPT1 and CDPS74/BrePT associations in significant amounts on UV chromatograms (Fig. 3). Their LC-MS/MS characteristics (retention time, MSn spectra) are similar to those of the product of the CDPS74/NotF association. These results are consistent with previous *in vitro* studies showing that EchPT1, BrePT, and NotF catalyse reverse prenylation at C2 on cWP^34–36^.

We purified three compounds and characterised them by NMR to determine the activity of CdpC3PT on cWL, showing reverse prenylation at C3, reverse prenylation at C2, and regular prenylation at N1. CdpC3PT is a versatile enzyme known to be active on a large set of CDPs *in vitro*. These include cWL, for which the reverse prenylation at C3 is its major activity^38,50^. Our results represent the first demonstration of CdpC3PT reverse prenylation of cWL at C2 and regular prenylation at N1. Similarly, the concomitant expression of CdpC3PT with a cWP-producing NRPS in *Aspergillus* resulted in the production of three prenylated cWPs with reverse prenylation at C3, reverse prenylation at C2, and regular prenylation at N1^15^.

We also observed conversion of cWW by PTs in *E. coli*. CTrpPT overexpression led to regular prenylation at C7 and reverse prenylation at N1, as previously described *in vitro*^45^. In addition, AnaPT overexpression resulted in the accumulation of the singly C3-reverse prenylated cWW. The diprenylated form of cWW was not detected *in vivo*, in contrast to the results of the *in vitro* assay with AnaPT^37^. Finally, we observed no activity on cWW for CdpC2PT, CdpC7PT, CdpNPT or RoqD. Among these four PTs, we only detected CdpC7PT and RoqD in insoluble fractions and obtained no evidence of the expression of CdpC2PT in *E. coli*. However, we did not observe a clear correlation between the detected expression level and activity; although EchPT1 was not detected by SDS-PAGE, it was active in *E. coli*, as shown by the production of prenylated cWP upon expression (Figs 2 and 3). Kinetic parameters of CdpC2PT and CdpC7PT for DMAPP and cWW indicate a high affinity for both substrates^43,44^. CdpNPT has a clearly weak affinity for DMAPP, with a K_M_ value of 650 µM^39^, suggesting that the intracellular level of DMAPP could limit its activity. One common point of the inactive PTs under our conditions was the CDP substrate, cWW. It may be informative to assess the activities of CdpC2PT, CdpC7PT, and CdpNPT with other CDP substrates, such as cWP (CdpNPT and CdpC7PT)^39,44^ or cWL (CdpC2PT and CdpC7PT)^43,44^.

In the context of the discovery of new bioactive compounds, combinatorial engineering of biosynthetic pathways is a highly promising approach. The system presented here could be very effective for the discovery of novel prenylated indole alkaloids for several reasons. First, the activity of many CDPSs that can incorporate tryptophan have been described^28–31^, resulting in a large variety of indole CDPs that are potential substrates for PTs. Second, we have recently shown that CDPSs expressed in *E. coli* can incorporate non-canonical amino acids into CDPs, thus broadening the variety of synthesised CDPs^33^. The *in vivo* activity of PTs on CDPs containing non-canonical amino acids and the generated chemical diversity merit further investigation. Third, the production of prenylated CDPs in *E. coli* culture supernatants constitutes a technological advantage. SPE treatments of culture supernatants resulted in efficient prenylation of CDPs in tractable samples deprived of salts and easily amenable to concentration and lyophilization. Such properties could be highly useful for screening activity in medium- to high-throughput strategies. Finally, purified prenylated CDPs were obtained in the range of 2 to 26 mg from 0.5 to 1 litre of culture supernatant, suggesting higher concentrations in culture supernatants. Furthermore, we observed high amounts of unconverted CDPs in culture supernatants, whether they were treated or not by SPE (see Fig. 3). This suggests that CDPs escape the activity of the PTs, probably due to leakage into the culture supernatants. Several synthetic biology approaches have been developed to limit such leakage in reconstructed biosynthetic pathways. These approaches aim to bring together and spatially organise the enzymes involved in the same pathway in the cell^51^. Some of these approaches, such as gene fusions or enzyme scaffolding, could be used to optimise CDP utilisation by PTs. Thus, our study paves the way to the discovery of novel bioactive alkaloid DKPs through *E. coli* engineering.

## Methods

### Bacterial strains and media

*E. coli* DH5α (Thermofisher) was used for cloning experiments and *E. coli* BL21AI (Thermofisher) for metabolite production. Bacteria of strain DH5α were rendered chemically competent using the high efficiency transformation protocol described by Inoue *et al*.^52^. Bacteria of strain BL21AI were rendered electrocompetent using the method of Sambrook *et al*.^53^. LB medium was used for standard protocols. Minimal medium, used for metabolite production, consisted of a base of M9 minimal salts supplemented with oligo-elements and vitamins as previously described^29^. Carbon sources consisted of 0.5% glucose for starter cultures and 0.05% glucose, 0.5% glycerol, 0.2% lactose, and 0.2% arabinose for DKP production cultures. Ampicillin (200 µg/ml), kanamycin (50 mg/ml), and chloramphenicol (25 µg/ml) were added as required.

### DNA manipulations and plasmids

DNA was manipulated using standard protocols unless otherwise stated^53^. Molecular biology enzymes were purchased from New England Biolabs (Ozyme, France) unless otherwise stated. Oligonucleotides were obtained from Sigma Aldrich. Plasmid preparations were made using Plasmid MiniPrep or Plasmid MidiPrep DNA preparation kits obtained from Sigma-Aldrich. DNA fragments were gel-purified using the QIAquick gel extraction kit (Qiagen).

Plasmids pIJ196-CDPS14, pIJ196-CDPS68 and pIJ196-CDPS74 encode CDPS14 from *Streptomyces cattleya*, CDPS68 from *Streptomyces* sp. NRRL F-5053, and CDPS74 from *Streptomyces* sp. NRRL S-1868, respectively. Their constructions has been described previously^28,29^. They carry the *col*E1 origin of replication and ampicillin resistance gene and the expression of the CDPS genes is under the control of the constitutive PT5 promoter followed by two *lacO* sequences. pIJ196-CDPS refers to a pIJ196 derivative that allows the expression of a CDPS.

Plasmid pIJ194 was constructed for cloning PT genes. It is derived from pRSFDuet-1 (RSF1030 replicon and kanamycin resistance gene; Novagen). First, the single *Xba*I site in pRSFDuet-1 was eliminated by digestion with *Xba*I, filling in with T4 DNA polymerase, and self-ligation using T4 DNA ligase. Second, *Spe*I and *Xba*I sites were introduced in place of the *Pfo*I site (upstream of the T7 promoter-1; position 3766 of pRSFDuet-1) and the *Eco*O109I site (downstream of the second T7 transcription terminator; position 477 of pRSFDuet-1), respectively. A DNA cassette containing these replacements was generated by PCR synthesis by overlapping extension. Three PCRs were performed using Phusion DNA polymerase, pRSFDuet-1 as a matrix (5 ng), and oligonucleotides 5′-GGGCCAGACTGGAGGTGGCAAC-3′ and 5′-CATAAGGGAGAGCGTCGAGA**ACTAGT**CACCATCGAATGCGCAAAACC-3′ for PCR1, 5′-GGTTTTGCGCCATTCGATGGTG**ACTAGT**TCTCGACGCTCTCCCTTATG-3′ and 5′-CCCTCAAGACCCGTTTAG**TCTAGA**AAGGGGTTATGCTAGTTATTG-3′ for PCR2, and 5′-CAATAACTAGCATAACCCCTT**TCTAGA**CTAAACGGGTCTTGAGGG-3′ and 5′-CAGTTTCATTTGATGCTCGATG-3′ for PCR3. PCR fragments 1 to 3 were gel-purified and used as a matrix (5 ng each) in PCR4 using oligonucleotides 5′-GGGCCAGACTGGAGGTGGCAAC-3′ and 5′-CAGTTTCATTTGATGCTCGATG-3′. PCRs were performed according to the manufacturer’s instructions: 98 °C for 30 s; 30 cycles of 10 s at 98 °C, 30 s at 64 °C and 15 s at 72 °C; and 72 °C for 10 min. The DNA fragment from PCR4 was gel-purified and digested with *Acl*I and *Age*I. After gel purification, the *Acl*I-*Age*I fragment was cloned between the *Acl*I and *Age*I sites of the *Xba*I-free pRSFDuet-1 described above. The DNA sequence between the *Acl*I and *Age*I sites was verified by DNA sequencing (Eurofins Genomics).

PT sequences were obtained from databases. Synthetic PT genes, optimised for expression in *E. coli* and designed with a 5′ *Nco*I site carrying the start codon and a 3′ *Xho*I site located after the stop codon, were obtained from Sigma-Aldrich (Supplementary Table S1). Synthetic PT genes were purchased and cloned in a commercial vector. For each construct, an *Nco*I-*Xho*I fragment carrying the PT gene was gel-purified and cloned between the *Nco*I and *Xho*I sites of pIJ194. The DNA sequence of the cloned fragment was verified by DNA sequencing (Eurofins Genomics). pIJ194-PT refers to a pIJ194 derivative carrying a PT gene cloned as described.

Plasmids pJBEI-3085 and pJBEI-3122 (p15A origin of replication and chloramphenicol resistance gene) encode the mevalonate-dependent pathway for DMAPP and IPP production^47^. They were kindly provided by the laboratory of Taek Soon Lee.

Plasmids intended for electroporation were dialyzed against deionised water using a 0.022 µm membrane (Millipore).

### Analysis of PT expression

Electrocompetent BL21AI bacteria were transformed with pIJ194-PT or empty pIJ194 and spread on LB agar plates containing 50 µg/ml kanamycin at 37 °C. Transformants were then grown overnight at 37 °C in liquid minimal medium supplemented with 50 µg/ml kanamycin and 0.5% glucose. These starter cultures were used to inoculate minimal medium containing 0.05% glucose, 0.5% glycerol, 0.2% lactose, 0.2% arabinose, and kanamycin. After 3.5 h at 37 °C, cultures were transferred to 20 °C for 24 h. Whole cell fractions were prepared by resuspending the pellet of a 1-ml culture in 200 µl of 1X SDS-PAGE loading buffer^53^ and heating at 100 °C for 30 min. Soluble and insoluble fractions were prepared by chemical lysis. Pellets of 2-ml cultures were frozen at −80 °C for one night. After thawing at 4 °C, they were resuspended in 400 µl of 100 mM Tris HCl pH 8, 300 mM KCl, 0.5% Triton X100 and 1 mg/ml lysozyme. PMSF was then added to a final concentration of 0.5 mM. After 1 h of agitation at 4 °C, 4 µl of 1 M MgCl_2_ and 10 units benzonase were added and agitation was continued for another hour at 4 °C. After centrifugation at 20,000 x g for 30 min, supernatants were saved as soluble fractions. Pellets were resuspended in 400 µl 1X SDS-PAGE loading buffer and heated at 100 °C for 30 min. Samples were conserved at −20 °C until SDS-PAGE analysis.

Samples were analysed by 12% SDS-PAGE and Coomassie Brilliant blue staining. Gel images were captured using the Infinity1000/26MX system (Vilber Lourmat). Gel pieces with bands at approximately 40–55 kDa appearing in the samples corresponding to the overexpression of one PT were excised from the gels and treated for peptide mass fingerprinting (PMF) and peptide MS/MS sequencing. Gel pieces were thoroughly washed three times with 100 mM NH_4_HCO_3_ and then two times with 50 mM NH_4_HCO_3_ in 50% CH_3_CN before drying. Trypsin digestion was performed by covering the dried gel pieces with 50 µl of 50 mM NH_4_HCO_3_ solution containing 250 ng trypsin (sequencing grade) and incubation at 50 °C for 2 h. Samples were then acidified with 5% trifluoroacetic acid (TFA) and 0.5 µl was spotted onto a MALDI plate and concentrated using the dried-droplet method with 0.5 µl of a 3-cyano-4-hydroxycinnamic acid matrix solution at 10 mg/ml in 50% CH_3_CN in 0.1% TFA. MS and PSD (Post-Source Decay) MS/MS spectra were acquired using an ABI 4800 MALDI-TOF/TOF mass spectrometer (Applied Biosystems, Foster City, USA) in positive reflectron mode. Each MS spectrum was the result of 1000 shots. Analyses of the peptide mass fingerprints from baseline-corrected, noise-filtered de-isotoped spectra were performed using Data Explorer® processing software (Version 4.9, Applied Biosystems, Foster City, USA) and proteins identified by an on-line MASCOT search (http://www.matrixscience.com). Search parameters against the NCBI protein database were as follows: enzymatic cleavage, trypsic; restriction in « other Fungi » taxonomy; variable modifications, Met oxidation, deamidation (NQ); missed cleavages, 1; MS tolerance, 150 ppm; and MS/MS tolerance, 0.25 atomic mass unit. Each MS/MS spectrum was the result of 2000 shots. The sequence of the tryptic fragments was identified by using MASCOT search engine (same search parameters as for PMF analyses) after smoothing and noise-filtered processing by Data Explorer® software.

### Cultivation for analytical scale DKP production

Electrocompetent BL21AI bacteria were simultaneously transformed with pIJ196-CDPS and pIJ194-PT (or empty pIJ194). Transformants were selected on LB agar plates containing ampicillin (200 µg/ml) and kanamycin (50 µg/ml). A few transformant colonies were used to inoculate 5 ml minimal medium containing antibiotics and 0.5% glucose and starter cultures were grown overnight at 37 °C. Cultures for the production of metabolites were performed in 15 ml minimal medium containing appropriate antibiotics and 0.05% glucose, 0.5% glycerol, 0.2% lactose, and 0.2% arabinose (in a 150 ml Erlenmeyer flask). Medium was prewarmed to 37 °C and inoculated with a starter culture at 1/50 of the culture volume. Cultures were grown 3.5 h at 37 °C and transferred to 20 °C for 48 h.

For DKP production with DMAPP pathway engineering, electrocompetent BL21AI bacteria were transformed with pJBEI-3085 or pJBEI-3122. Transformants selected on chloramphenicol (25 µg/ml) were rendered electrocompetent as described above. Electrocompetent BL21AI bearing pJBEI-3085 or pJBEI-3122 were treated as above except that 25 µg/ml chloramphenicol was added to all cultures.

### Preparation of bacterial culture extracts for metabolite analysis

Bacterial supernatants were recovered after centrifugation at 20,000 × g for 10 min. For solid phase extraction (SPE), 5 ml unacidified culture supernatant was loaded onto 30 mg Strata-X polymeric sorbent (Phenomenex) previously conditioned and equilibrated as recommended by the manufacturer. After washing with 1 ml 5% methanol, the elution was carried out with 600 µl methanol. Samples were conserved in well screwed tubes at 4 °C before LC-MS/MS analysis.

Ethyl acetate extractions were performed on bacterial lysates. Sonicated lysates were prepared by resuspension of the bacterial pellet of a 1-ml culture in 1 ml of 50 mM Tris-HCl pH 7.5 and sonication using a Vibra-Cell^TM^ ultrasonic processor equipped with a microtip (10 burst of 15 s at 30% power separated by intervals of 1 min on ice). The efficiency of sonication was verified by optical microscopy. Chemical lysates were prepared as above. Equal volumes of lysate and ethyl acetate were mixed in a 2-ml Eppendorf tube and vortexed for 15 min using a Vibrax^®^ agitator. After centrifugation 5 min at 20,000 × g, the organic upper phase was saved and the aqueous phase was extracted a second time with 500 µl ethyl acetate. After centrifugation and recovery of the organic phase, the two organic phases were pooled and evaporated in a SpeedVac (SPD121P concentrator equipped with a RVT5105 vapor trap; Thermofisher). Dried extracts were conserved at 4 °C and resuspended in methanol before LC-MS/MS analysis.

### LC-MS/MS analysis

LC separations were carried out using an Agilent 1100 HPLC equipped with an ACE Excel 3 C18-PFP column (150 × 4.6 mm, 3 µm, 100 Å) and a flow rate of 0.6 ml/min. The solvents were 0.1% formic acid in water (A) and 90% CH_3_CN in water containing 0.1% formic acid (B). HPLC runs started with 5 min at 30% B followed by a linear gradient of 30% to 100% B in 28 min. After 3 min at 100% B in A, the system returned to 30%B in A in 2 min and the column was equilibrated for 25 min. The Agilent 1100 HPLC machine was coupled *via* a split system to an Esquire HCT ion trap mass spectrometer (Bruker Daltonik GmbH). All MS and MS/MS spectra were acquired in positive mode within the 50 to 600 m/z range with an automatic selection of parent-ions for MS/MS fragmentation.

### Scale-up production and isolation of DKP for structural analysis

Scale-up productions were performed as the cultivations for the analysis of DKP production, except that the starter cultures were 25 ml in 250-ml Erlenmyer flasks and the production cultures were 500 ml in 3-L Erlenmeyer flasks. After centrifugation, 500 ml culture supernatant was loaded onto 1 g Strata-X polymeric sorbent (Phenomenex) previously conditioned and equilibrated as recommended by the manufacturer. After washing with 12 ml 5% methanol, elution was carried out with 10 ml methanol. Samples were conserved in well-screwed tubes at 4 °C.

Purification was carried out using an Hitachi LP1100/LP3101 HPLC equipped with a Purospher Star RP-18e column (250 × 10 mm, 5 µm; VWR) mounted with a guard column. Solvents A and B were the same as for the analytical conditions. The flow rate was 4.75 ml/min. HPLC was carried out according to the following conditions: **4** and **5** (products of CDPS74 and Not or FtmPT1 activities, respectively), 5-min step with 30% solvent B in solvent A followed by a linear gradient of 30–50% solvent B in solvent A in 20 min; **6**, **7**, and **8** (products of CDPS68 and CdpC3PT activities), 5-min step with 40% solvent B in solvent A followed by a linear gradient of 40–51% solvent B in solvent A in 22 min; **9** (product of CDPS14 and AnaPT activities), 5-min step with 40% solvent B in solvent A followed by a linear gradient of 40–60% solvent B in solvent A in 20 min; **10** and **11** (products of CDPS14 and CTrpPT activities), 5-min step with 30% solvent B in solvent A followed by a linear gradient of 30–50% solvent B in solvent A in 30 min; all gradients were followed by a linear gradient to 100% solvent B in solvent A in 2 min, a 2-min step at 100% solvent B in solvent A, a linear gradient to the initial condition of solvent B in solvent A in 2 min and a 30-min equilibration step in the initial condition. Peaks containing the desired compound were identified after collection at the peak maximum and direct analyses by ESI-MS and MS/MS mass spectrometry using an Esquire HCT ion trap mass spectrometer set in positive mode. Fractions containing the desired compound were pooled, diluted with H_2_O to reduce CH_3_CN content, and lyophilised. The amount of compound was determined by weighing. Purity was evaluated by LC analysis using a Lachrome Elite system (VWR) equipped with a L-2455 diode array detector and L-2200 autosampler. Chromatographic conditions were the same as those for LC-MS/MS.

### NMR spectroscopy

NMR experiments were recorded on a Bruker Avance III spectrometer equipped with a TCI cryoprobe and operating at a ^1^H frequency of 500.3 MHz. Spectra were recorded at 26 °C in DMSO-*d*_6_, (99.96%, Euriso-top). ^1^H and ^13^C resonances were assigned through the analysis of 1D ^1^H, 1D ^13^C DEPTQ, 2D ^1^H-^1^H COSY, 2D ^1^H-^1^H ROESY, 2D ^1^H-^13^C HSQC, 2D ^1^H-^13^C HMBC spectra. ^1^H and ^13^C chemical shifts were referenced to the DMSO solvent signal (*δ* 2.50 and 39.5 ppm, respectively). ^15^N resonances were assigned via the analysis of 2D ^1^H-^15^N HMBC. ^15^N chemical shifts were referenced indirectly to liquid ammonia using the chemical shift of the lock solvent. NMR experiments were processed and analysed using the Bruker TOPSPIN 3.5 program. NMR data for compounds **4**–**11** are described below.

Compound **4**. ^1^H NMR (500.3 MHz, DMSO): *δ* 10.67 (s, 1H, H1), 7.49 (d, *J* = 7.9 Hz, 1H, H4), 7.35 (dt, *J* = 8.0, 0.9 Hz, 1H, H7), 7.05 (ddd, *J* = 8.0, 7.0, 1.1 Hz, 1H, H6), 6.96 (ddd, *J = *7.9, 7.0, 1.1 Hz, 1H, H5), 6.22 (br s, 1H, H12), 6.21 (dd, *J* = 17.4, 10.5 Hz, 1H, H2’), 5.07 (dd, *J* = 17.4, 1.2 Hz, 1H, H1’_*trans* to H2’_), 5.05 (dd, *J* = 10.5, 1.2 Hz, 1H, H1’_*cis* to H2’_), 4.33 (ddd, *J* = 9.7, 4.7, 1.2 Hz 1H, H11), 4.19 (m, 1H, H14), 3.52 (dd, *J* = 15.1, 4.8 Hz, 1H, H10_*gauche* to H11_), 3.46 (m, 1H, H17), 3.36 (m, 1H, H17’), 2.95 (dd, *J* = 15.1, 9.7 Hz, 1H, H10’_*anti* to H11_), 2.10 (m, 1H, H19), 1.89–1.75 (m, 3H, H19’, H18 and H18’), 1.51 (s, 3H, H4’), 1.50 (s, 3H, H5’); ^13^C NMR (125.8 MHz, DMSO): *δ* 169.4 (C13), 165.7 (C16), 146.4 (C2’), 141.4(C2), 134.8 (C8), 128.6 (C9), 120.7 (C6), 118.6 (C5), 117.8 (C4), 111.3 (C1’), 111.1 (C7), 104.4 (C3), 58.5 (C14), 55.1 (C11), 44.8 (C17), 38.9 (C3’), 27.9 (C5’), 27.8 (C4’), 27.6 (C19), 25.2 (C10), 22.3 (C18); ^15^N NMR (50.7 MHz, DMSO): *δ* 134.3 (N1), 124.5 (N15), 115.5 (N12).

Compound **5**. ^1^H NMR (500.3 MHz, DMSO): *δ* 10.70 (s, 1H, H1), 7.46 (d, *J* = 7.9 Hz, 1H, H4), 7.25 (dt, *J* = 8.0, 0.9 Hz, 1H, H7), 7.21 (s, 1H, H12), 6.98 (ddd, *J* = 8.0, 7.0, 1.2 Hz, 1H, H6), 6.91 (ddd, *J* = 7.9, 7.0, 1.1 Hz, 1H, Η5), 5.32 (m, 1H, H2’), 4.24 (t, *J* = 5.6 Hz, 1H, H11), 3.98 (ddd, *J* = 10.0, 6.8, 1.2 Hz, 1H, H14), 3.54 (dd, *J* = 15.9, 7.7 Hz, 1H, H1’), 3.42–3.36 (m, 2H, H1’ and H17), 3.21 (dd, *J* = 14.7, 5.3 Hz, 1H, H10), 3.15 (ddd, *J* = 11.5, 9.1, 4.2 Hz, 1H, H17’), 3.00 (dd, *J* = 14.7, 6.0 Hz, 1H, H10’), 1.88 (dtd, *J* = 12.1, 7.1, 2.9 Hz, 1H, H19), 1.72 (d, *J* = 1.2 Hz, 3H, H4’), 1.70 (d, *J* = 1.3 Hz, 3H, H5’), 1.60 (m, 1H, H18), 1.38 (m, 1H, H18’), 1.10 (m, 1H, H19’); ^13^C NMR (125.8 MHz, DMSO): *δ* 168.4 (C13), 165.4 (C16), 137.1 (C2), 135.3 (C8), 132.1 (C3’), 128.0 (C9), 121.6 (C2’), 120.2 (C6), 118.2 (C5), 118.1 (C4), 110.5 (C7), 104.2 (C3), 58.4 (C14), 55.4 (C11), 44.5 (C17), 27.5 (C19), 26.2 (C10), 25.5 (C5’), 24.9 (C1’), 21.6 (C18), 17.8 (C4’); ^15^N NMR (50.7 MHz, DMSO): *δ* 135.2 (N1), 126.2 (N15), 117.1(N12).

Compound **6**. ^1^H NMR (500.3 MHz, DMSO): *δ* 10.51 (s, 1H, H1), 8.28 (d, *J* = 3.6 Hz, 1H, H15), 7.75 (d, *J* = 3.8 Hz, 1H, H12), 7.43 (d, *J* = 7.8 Hz, 1H, Η4), 7.31 (dt, *J* = 8.0, 0.9 Hz, 1H, H7), 7.01 (ddd, *J* = 8.0, 7.0, 1.2 Hz, 1H, H6), 6.91 (ddd, *J* = 8.0, 7.0, 1.1 Hz, 1H, H5), 6.17 (dd, *J* = 17.4, 10.5 Hz, 1H, H2’), 5.07 (dd, *J* = 17.4, 1.3 Hz, 1H, H1’_*trans* to H2’_), 5.03 (dd, *J* = 10.5, 1.3 Hz, 1H, H1’_*cis* to H2’_), 3.95 (m, 1H, H11), 3.61 (m, 1H, H14), 3.31 (dd, *J* = 14.5, 4.8 Hz, 1H, H10), 3.11 (dd, *J* = 14.5, 8.2 Hz, 1H, H10’), 1.70 (m,1H, H18), 1.485 (s, 3H, H5’), 1.480 (s, 3H, H4’), 1.41 (ddd, *J* = 13.5, 9.0, 4.8 Hz, 1H, H17), 1.23 (ddd, *J* = 13.5, 9.6, 5.3 Hz, H17’), 0.84 (d, *J* = 6.8 Hz, 3H, H19), 0.83 (d, *J* = 6.8 Hz, 3H, H20); ^13^C NMR (125.8 MHz, DMSO): *δ* 167.62 (C13), 167.59 (C16), 146.5 (C2’), 141.3 (C2), 134.8 (C8), 129.0 (C9), 120.4 (C6), 118.23 (C5), 118.04 (C4), 110.9 (C1’), 110.8 (C7), 104.7 (C3), 55.9 (C11), 52.9 (C14), 44.7 (C17), 38.8 (C3’), 31.1 (C10), 28.02 (C4’), 27.97 (C5’), 23.5 (C18), 23.0 (C19), 21.3 (C20); ^15^N NMR (50.7 MHz, DMSO): *δ* 133.4 (N1), 117.9 (N15), 117.7 (N12).

Compound **7**. ^1^H NMR (500.3 MHz, DMSO): *δ* 8.07 (d, *J* = 2.6 Hz, 1H, H12), 7.93 (d, *J* = 2.8 Hz, 1H, H15), 7.55 (dt, *J* = 8.0, 1.0 Hz, 1H, H4), 7.31 (dt, *J* = 8.25, 0.9 Hz, 1H, H7), 7.07 (ddd, *J* = 8.2, 7.0, 1.2 Hz, 1H, H6), 7.02 (s, 1H, H2), 6.95 (ddd, *J* = 8.0, 7.0, 1.0 Hz, 1H, H5), 5.27 (t of hept., *J* = 7.0, 1.4 Hz, 1H, H2’), 4.68 (d, *J* = 7.0 Hz, 2H, H1’), 4.09 (m, 1H, H11), 3.40 (m, 1H, H14), 3.26 (dd, *J* = 14.4, 3.7 Hz, 1H, H10), 2.95 (dd, *J* = 14.4, 4.7 Hz, 1H, H10’), 1.79 (d, *J* = 1.3 Hz, 3H, H4’), 1.69 (d, *J* = 1.3 Hz, 3H, H5’), 1.16 (m,1H, H18), 0.65 (ddd, *J* = 13.5, 9.1, 4.7 Hz, 1H, H17), 0.50 (d, *J* = 6.6 Hz, 3H, H19), 0.37 (d, *J* = 6.6 Hz, 3H, H20), −0.04 (m, 1H, H17’); ^13^C NMR (125.8 MHz, DMSO): *δ* 167.5 (C13), 167.0 (C16), 135.6 (C8), 135.1 (C3’), 128.4 (C9), 127.7 (C2), 120.9 (C6), 120.5 (C2’), 119.4 (C4), 118.6 (C5), 109.5 (C7), 108.0 (C3), 55.5 (C11), 52.4 (C14), 43.9 (C17), 43.3 (C1’), 29.0 (C10), 25.4 (C5’), 22.9 (C18), 22.7 (C20), 21.3 (C19), 17.8 (C4’); ^15^N NMR (50.7 MHz, DMSO): *δ* 138.1 (N1), 120.0 (N15), 117.1 (N12).

Compound **8**: ^1^H NMR (500.3 MHz, DMSO): *δ* 7.98 (s, 1H, H15), 7.16 (dd, *J* = 7.5, 1.3 Hz, 1H, Η4), 6.99 (td, *J* = 7.6, 1.3 Hz, 1H, H6), 6.62 (td, *J* = 7.5, 1.1 Hz, 1H, H5), 6.53 (dd, *J* = 7.8, 1.2 Hz, 1H, Η7), 6.44 (d, *J* = 1.1 Hz, 1H, H1), 5.99 (dd, *J* = 17.4, 10.9 Hz, 1H, H2’), 5.39 (d, *J* = 1.1 Hz, 1H, H2), 5.08 (dd, *J* = 10.8, 1.4 Hz, 1H, H1’_*cis* to H2’_), 5.04 (dd, *J* = 17.3, 1.4 Hz, 1H, H1’_*trans* to H2’_), 4.00 (ddd, *J* = 6.8, 5.0, 1.9 Hz, 1H, H14), 3.86 (ddd, *J* = 11.1, 6.4, 1.8 Hz, 1H, H11), 2.35 (dd, *J* = 12.7, 6.4 Hz, 1H, H10_*gauche*_ _to H11_), 2.23 (dd, *J* = 12.7, 11.1 Hz, 1H, H10_*anti to H11*_), 1.85 (m,1H, H18), 1.75 (m, 1H, H17), 1.43 (dt, *J* = 13.9, 6.6 Hz, 1H, H17’), 1.04 (s, 3H, H5’), 0.88 (s, 3H, H4’), 0.84 (d, *J* = 6.6 Hz, 3H, H19), 0.83 (d, *J* = 6.6 Hz, 3H, H20); ^13^C NMR (125.8 MHz, DMSO): *δ* 169.2 (C16), 166.1 (C13), 151.0 (C8), 144.1 (C2’), 129.0 (C9), 128.4 (C6), 124.7 (C4), 117.3 (C5), 114.0 (C1’), 108.5 (C7), 76.5 (C2), 60.7 (C3), 58.1 (C11), 52.8 (C14), 40.7 (C3’), 38.2 (C17), 36.5 (C10), 23.9 (C18), 22.7 (C4’), 22.6 (C19), 22.2 (C5’), 22.1 (C20); ^15^N NMR (50.7 MHz, DMSO): *δ* 144.5 (N12), 117.4 (N15), 85.8 (N1).

Compound **9**: ^1^H NMR (500.3 MHz, DMSO): *δ* 10.77 (d, *J* = 2 Hz, 1H, H20), 7.55 (d, *J* = 7.9 Hz, 1H, H25), 7.53 (s, 1H, H15), 7.30 (d, *J* = 8.1 Hz, 1H, H22), 7.14 (d, *J* = 2.5 Hz, 1H, H19), 7.06 (dd, *J* = 7.1, 1.0 Hz, 1H, Η4), 7.04 (ddd, *J* = 8.1, 7.0, 1.2 Hz, 1H, H23), 6.97 (ddd, *J* = 8.0, 6.7, 1.1 Hz, 1H, H24), 6.95 (ddd, *J* = 7.5, 6.7, 1.3 Hz, 1H, H6), 6.58 (td, *J* = 7.5, 1.2 Hz, 1H, H5), 6.52 (d, *J* = 7.7 Hz, 1H, Η7), 6.38 (s, 1H, H1), 5.98 (dd, *J* = 17.4, 10.9 Hz, 1H, H2’), 5.29 (s, 1H, H2), 5.09 (dd, *J* = 10.9, 1.5 Hz, 1H, H1’_*cis* to H2’_), 5.06 (dd, *J* = 17.4, 1.5 Hz, 1H, H1’_*trans* to H2’_), 4.32 (m, 1H, H14), 4.25 (td, *J* = 8.9, 1.0 Hz, 1H, H11), 3.31 (dd, *J* = 15.1, 4.5 Hz, 1H, H17), 2.98 (dd, *J* = 15.1, 6.7 Hz, 1H, H17’), 2.64 (dd, *J* = 13.9, 9.2 Hz, 1H, H10_*gauche* to H11_), 2.12 (dd, *J* = 13.8, 8.6 Hz, 1H, H10_*anti* to H11_), 1.02 (s, 3H, H5’), 0.93 (s, 3H, H4’); ^13^C NMR (125.8 MHz, DMSO): *δ* 169.7 (C16), 168.7 (C13), 149.0 (C8), 144.2 (C2’), 136.0 (C21), 131.6 (C9), 127.9 (C6), 127.2 (C26), 124.9 (C4), 124.2 (C19), 120.9 (C23), 118.4 (C25), 118.3 (C24), 117.3 (C5), 113.9 (C1’), 111.3 (C22), 109.4 (C18), 108.6 (C7), 78.3 (C2), 61.2 (C3), 57.1 (C11), 55.4 (C14), 41.3 (C3’), 35.3 (C10), 24.7 (C17), 22.5 (C4’), 22.1 (C5’); ^15^N NMR (50.7 MHz, DMSO): *δ* 140.9 (N12), 131.4 (N20), 116.1 (N15), 81.8 (N1).

Compound **10**: ^1^H NMR (500.3 MHz, DMSO): *δ* 10.84 (d, *J* = 2.4 Hz, 1H, H20), δ 10.75 (d, *J* = 2.4 Hz, 1H, H1), 7.70 (d, *J* = 2.8 Hz, 1H, H15), 7.65 (d, *J* = 2.8 Hz, 1H, H12), 7.36 (d, *J* = 7.9 Hz, 1H, H25), 7.28 (dt, *J* = 8.1, 0.9 Hz, 1H, H22), 7.17 (dd, *J* = 8.1, 0.8 Hz, 1H, Η4), 7.04 (ddd, *J* = 8.2, 7.0, 1.2 Hz, 1H, H23), 6.95 (ddd, *J* = 8.0, 7.0, 1.1 Hz, 1H, H24), 6.87 (t, *J* = 7.2 Hz, 1H, H5), 6.81 (dd, *J* = 7.1, 1.0 Hz, 1H, H6), 6.63 (d, *J* = 2.3 Hz, 1H, H19), 6.53 (d, *J* = 2.5 Hz, 1H, Η2), 5.31 (t of hept., *J* = 7.3, 1.4 Hz, 1H, H2’), 3.89 (m, 1H, H14), 3.83 (m, 1H, H11), 3.44 (d, *J* = 7.3 Hz, 2H, H1’), 2.71 (dd, *J* = 14.8, 4.3 Hz, 1H, H17), 2.68 (dd, *J* = 14.7, 4.0 Hz, 1H, H10), 2.24 (dd, *J* = 14.4, 6.5 Hz, 1H, H17’), 2.08 (dd, *J* = 14.3, 6.9 Hz, 1H, H10’), 1.64 (d, *J* = 0.8 Hz, 3H, H4’), 1.59 (d, *J* = 0.8 Hz, 3H, H5’); ^13^C NMR (125.8 MHz, DMSO): *δ* 166.8 (C13 and C16), 136.1 (C21), 134.9 (C8), 131.8 (C3’), 127.4 (C26), 127.3 (C9), 124.47 (C19), 124.2 (C7 and C2), 122.2 (C2’), 120.8 (C23), 119.9 (C6), 118.7 (C5), 118.6 (C25), 118.3 (C24), 116.2 (C4), 111.3 (C22), 109.2 (C3), 108.8 (C18), 55.3 (C11 and C14), 30.2 (C10), 29.9 (C17), 29.0 (C1’), 25.4 (C5’), 17.7 (C4’); ^15^N NMR (50.7 MHz, DMSO): *δ* 132.1 (N20), 130.6 (N1), 118.4 (N12), 118.3 (N15).

Compound **11**: ^1^H NMR (500.3 MHz, DMSO): *δ* 10.84 (d, *J* = 2.4 Hz, 1H, H20), 7.76 (d, *J* = 2.7 Hz, 1H, H15), 7.75 (d, *J* = 2.8 Hz, 1H, H12), 7.35 (m, 2H, H7 and H25), 7.32 (ddd, *J* = 7.8, 1.4, 0.8 Hz, 1H, Η4), 7.29 (dt, *J* = 8.1, 0.9 Hz, 1H, H22), 7.04 (ddd, *J* = 8.2, 7.0, 1.2 Hz, 1H, H23), 7.02 (ddd, *J* = 8.3, 7.0, 1.4 Hz, 1H, H6), 6.96 (m, 1H, H5), 6.95 (m, 1H, H24), 6.65 (s, broad, 1H, Η2), 6.57 (m, 1H, H19), 6.02 (dd, *J* = 17.5, 10.7 Hz, 1H, H2’), 5.13 (dd, *J* = 10.7, 0.9 Hz, 1H, H1’_*cis* to H2’_), 5.11 (dd, *J* = 17.4, 0.9 Hz, 1H, H1’_*trans* to H2’_), 3.89 (m, 1H, H14), 3.84 (m, 1H, H11), 2.74 (dd, *J* = 14.4, 4.4 Hz, 1H, H17), 2.68 (m, 1H, H10), 2.30 (dd, *J* = 14.0, 6.7 Hz, 1H, H17’), 2.00 (dd, *J* = 14.3, 7.0 Hz, 1H, H10’), 1.63 (s, 3H, H5’), 1.61 (s, 3H, H4’); ^13^C NMR (125.8 MHz, DMSO): *δ* 166.9 (C13), 166.8 (C16), 144.0 (C2’), 136.0 (C21), 134.9 (C8), 129.1 (C9), 127.3 (C26), 125.1 (C2), 124.49 (C19), 120.9 (C23), 120.4 (C6), 118.8 (C4), 118.6 (C25), 118.4 (C5), 118.3 (C24), 113.4 (C7), 113.3 (C1’), 111.3 (C22), 108.7 (C18), 107.6 (C3), 58.5 (C3’), 55.3 (C14), 55.2 (C11), 30.3 (C17), 29.9 (C10), 27.5 (C5’), 27.4 (C4’).

## Supplementary information

Supplementary Information

## Data Availability

The datasets generated during and/or analysed during the current study are available from the corresponding authors on reasonable request.
