# Author Correction: Reprogramming *Escherichia coli* for the production of prenylated indole diketopiperazine alkaloids

**DOI:** 10.1038/s41598-019-51404-5

**Published:** 2019-10-15

**Authors:** Pavlina Dubois, Isabelle Correia, Fabien Le Chevalier, Steven Dubois, Isabelle Jacques, Nicolas Canu, Mireille Moutiez, Robert Thai, Muriel Gondry, Olivier Lequin, Pascal Belin

**Affiliations:** 1 Institute for Integrative Biology of the Cell (I2BC), CEA, CNRS, Univ. Paris-Sud, Université Paris-Saclay, 91198 Gif-sur-Yvette, cedex France; 2 SIMOPRO, CEA, 91198 Gif-sur-Yvette, cedex France; 3 Sorbonne Université, Ecole Normale Supérieure, PSL University, CNRS, Laboratoire des Biomolécules (LBM), 75005 Paris, France; 4 Present Address: Isabelle B. Jacques, APTEEUS, Institut Pasteur de Lille, Lille, France

Correction to: *Scientific Reports* 10.1038/s41598-019-45519-y, published online 25 June 2019

In Figure 2, panel 2C should not be shown. The correct Figure 2 appears below as Fig. 1.

**Figure 1:**
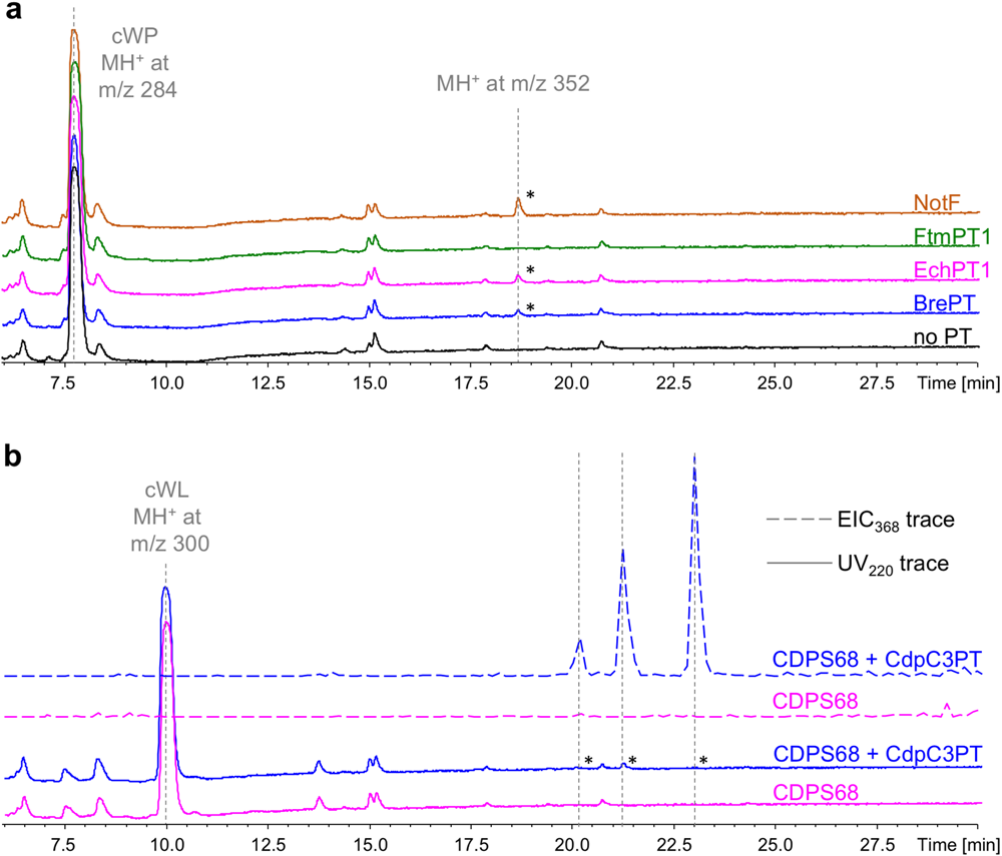
LC-MS/MS analysis of metabolite production by recombinant *E. coli*. Samples corresponding to 50 μl of culture supernatants were analysed. (**a**) SPE-treated bacterial supernatants of cultures of BL21AI expressing CDPS74 and BrePT (blue), EchPT1 (pink), FtmPT1 (green), NotF (orange) or CDPS74 alone (black) were analysed. UV traces recorded at 220 nm are shown between 6 and 30 min with the absorbance scale set from 0 to 700 mU. Asterisks highlight specific peaks for which the MS data are indicated. (**b**) SPE-treated bacterial supernatants of cultures of BL21AI expressing CDPS68 and CdpC3PT (blue) or CDPS68 alone (pink) were analysed. UV chromatograms recorded at 220 nm (UV_220_, plain lines) and extracted ion current at m/z 368 (EIC_368_, dotted lines) are shown between 6 and 30 min. The Y-axis of the UV_220_ traces was set from 0 to 700 mU and that of the EIC_368_ traces from 0 to 3,380,000.

