# Supplementary material for: Reprogramming Escherichia coli for the production of prenylated indole diketopiperazine alkaloids
Source: Sci Rep. 2019 Jun 25;9:9208. doi: 10.1038/s41598-019-45519-y (PMC6592928; doi:10.1038/s41598-019-45519-y)
Supplement: Supplementary file 1 — Supplementary Information [file 41598_2019_45519_MOESM1_ESM.pdf]

**Supplementary Information**

# Reprogramming *Escherichia coli* for the production of prenylated indole diketopiperazine alkaloids

Pavlina Dubois[1], Isabelle Correia[3], Fabien Le Chevalier[1], Steven Dubois[2], Isabelle Jacques[1,4], Nicolas Canu[1], Mireille Moutiez[1], Robert Thai[2], Muriel Gondry[1], Olivier Lequin[3], and Pascal Belin[1]

Pavlina Dubois and Isabelle Correia contributed equally.

[1]Institute for Integrative Biology of the Cell (I2BC), CEA, CNRS, Univ. Paris-Sud, Université Paris-Saclay, 91198, Gif-sur-Yvette cedex, France

[2]SIMOPRO, CEA, 91198, Gif-sur-Yvette cedex, France

[3]Sorbonne Université, Ecole Normale Supérieure, PSL University, CNRS, Laboratoire des Biomolécules (LBM), 75005, Paris, France

[4]Present address: Isabelle B. Jacques, APTEEUS, Institut Pasteur de Lille, Lille, France

**Correspondence to**:

Olivier Lequin, 33 1 44273113, olivier.lequin@upmc.fr

Pascal Belin, 33 1 69082662, pascal.belin@cea.fr

**Supplementary Table S1.** Sequence data of synthetic PT genes.

| PT | Sequence of synthetic gene† |
|---|---|
| AnaPT | CCATGGCCAGCCCGCTGAGCATGCAGACCGATAGCGTGCAAGGTACAGCAGAGAATAAAAGCTTAGAGACCAATGGCACCAGCAATGACCAGCAACTGCCGTGGAAAGTGCTGGGCAAATCTTTAGGTTTAC<br>CTACCATCGAGCAAGAGCAGTATTGGCTGAACACCGCACCGTACTTCAACAATTTACTGATCCAGTGCGGCTATGATGTTCACCAGCAGTACCAGTATTTAGCCTTCTACCATCGCCATGTTTTACCGGTGCTGG<br>GCCCGTTTATTCGTAGTAGCGCCGAGGCCAACTATATTAGCGGTTTCAGCGCCGAGGGTTATCCGATGGAGCTGAGCGTGAATTACCAAGCTAGTAAAGCCACAGTGCGTTTAGGTTGTGAGCCCGTTGGTGAG<br>TTTGCCGGCACAAGTCAAGATCCGATGAACCAGTTCATGACCCGCGAAGTGTTAGGTCGTTTAAGCCGTCTGGATCCGACCTTCGATCTGCGTTTATTTGATTATTTTGATAGCCAGTTTCTTTAACCACAAGC<br>GAAGCCAATTTAGCCGCCAGCAAGCTGATTAAACAGCGTCGTCAGAGCAAAGTGATTGCCTTCGATCTGAAGGACGGCGCCATCATTCCTAAAGCCTATTTCTTTTTAAAGGGTAAATCTTTAGCCAGTGGTAT<br>CCCGGTTCAAGATGTGGCATTCAATGCCATCGAAAGTATCGCCCCGAAGCAGATCGAGAGTCCGCTGCGTGTGCTGCGCACATTCGTTACCAAACTGTTCAGCAAGCCGACCGTGACCAGCGATGTGTTCATTT<br>TAGCCGTTGATTGCATCGTGCCGGAAAAGAGTCGCATCAAACTGTACGTGGCAGACAGTCAGCTGAGTCTGGCCACACTGCGTGAGTTCTGGACTTTAGGTGGTAGCGTTACCGATAGCGCCACAATGAAAGG<br>TTTAGAAATCGCCGAGGAACTGTGGCGCATTTTACAATATGATGACGCCGTGTGCAGCCATAGCAACATGGATCAGCTGCCTCTGGTTGTGAACTACGAACTGAGCAGCGGTAGTGCCACCCCTAAGCCGCAG<br>CTGTATCTGCCGCTGCATGGTCGCAATGATGAAGCAATGGCCAACGCTTTAACCAAATTCTGGGACTATTTAGGTTGGAAGGGTTTAGCCGCACAGTATAAAAAAGATCTGTATGCCAACAATCCGTGCCGCA<br>ATCTGGCCGAGACCACAACAGTTCAGCGTTGGGTGGCCTTTAGCTATACCGAAAGCGGCGGTGCCTATTTAACCGTGTATTTTCACGCCGTGGGTGGCATGAAGGGTAATCTGTAATGACTCGAG |
| BrePT | CCATGGCCACAGCACCCGAATTACGCGCCCCGGCTGGTCATCCGCAAGAACCGCCCGCTCGTAGTAGTCCCGCTCAAGCTCTGAGTAGCTATCACCACTTCCCGACCAGCGATCAAGAACGCTGGTATCAAGAA<br>ACCGGCAGCTTATGTAGCCGTTTTCTGGAAGCCGGCCAGTACGGTTTACATCAGCAATACCAGTTCATGTTTTTCTTCATGCACCATTTAATTCCGGCTTTAGGCCCGTATCCGCAGAAATGGCGCAGTACCATTA<br>GCCGCAGCGGTCTGCCGATTGAATTCTCTTTAAATTTCCAGAAAGGTAGCCATCGTTTACTGCGTATCGGTTTTGAGCCGGTGAACTTTTTAAGTGGTAGCAGCCAAGATCCGTTCAATCGCATTCCGATTGCCGA<br>TCTGCTGGCACAACTGGCCCGCTTACAGCTGCGCGGCTTTGACACCCAGTGCTTTCAGCAGCTGCTGACCCGCTTTCAACTGAGTCTGGATGAGGTGCGTCAACTGCCGCCGGACGATCAGCCGCTGAAAAGCCA<br>AGGTGCCTTCGGCTTCGATTTTAATCCGGACGGTGCCATTTTAGTGAAGGGTTACGTGTTCCCGTATTTAAAAGCAAAGGCAGCTGGTGTGCCGGTGGCCACACTGATTGCCGAAAGCGTTCGCGCCATTGACGC<br>AGATCGCAACCAGTTTATGCACGCCTTCTCTTTAATCAACGACTACATGCAAGAAAGCACCGGTTATAACGAGTATACCTTTTTAAGCTGCGATCTGGTGGAAATGAGCCGCCAGCGCGTGAAGATCTACGGCGC<br>CCATACCGAAGTTACTTGGGCAAAAATCGCCGAAATGTGGACTTTAGGTGGCCGTCTGATTGAAGAGCCGGAGATTATGGAAGGTTTAGCCCGTCTGAAACAGATTTGGTCTTTATTACAGATCGGCGAGGGTA<br>GCCGTGCCTTTAAAGGCGGCTTCGACTATGGCAAAGCCAGCGCCACCGATCAGATTCCGAGTCCGATCATCTGGAACTACGAGATCAGCCCGGGCAGCAGCTTTCCGGTGCCGAAATTTTATTTACCGGTGCACG<br>GCGAAAACGATTTACGTGTTGCACGCTCTTTAGCCCAGTTCTGGGATAGTCTGGGTTGGAGTGAGCACGCATGCGCATATCCGGACATGCTGCAGCAGCTGTACCCGGATCTGGATGTGAGCCGCACAAGCCGTC<br>TGCAGAGCTGGATCAGCTACAGCTATACCGCCAAAAAGGGTGTGTACATGAGCGTGTACTTCCACAGCCAGAGCACCTATCTGTGGGAAGAAGATTAATGACTCGAG |
| CdpC2PT | CCATGGCCGCAATTAATAGCCGTTGCGGTCTGGCTTGTAAACGCCATAGCGAGAGCGGCGAAGCCGAATATACCAATCGCGTTACTTTAAGCCCGATCGAAGCACTGGCCCGCTACTGGACCAGTCCGACCGAA<br>CATCACACCCAGTGGCAGAACGACACCGCCCCGCTGTTTAGTCGTCTGCTGGAGGCCGCACAGTATGATGTGCACGACCAGTACCAGTATTTATTATTTATGTTTGGCCACATTATTCCGTCTTTAGGTCCGTATC<br>CGCAGCGTTGGAAAAGCACCATGACAACTTTAGGCATTCCGCTGGAATTTAGCATCAATTACCAGAAGCACGGCAAGGAGACCGTTCGCGTGGCTTTAGAACCGGTGAGCGTGGTGAGTGGTACCGACGAGGA<br>CCCGTTTAATCTGATCACCACCCGCAAGTTCATGAGCGACATTGCCAATTTAGGTTTAGAAAGCTTTGACATGGAGCTGCACGACTATTTAATCAACGCCTTCTTTCCGAGCGCCAATGAGATCCGTGATCTGCAA<br>GCTAGTGGTTACGGCAAGCGTGGTGGTGAGCAGAAAAGTCCGAGCGCCTTCGGTTTTGATTTAAAAGCCGGCGGTTGCATTACCGTGAAAGGCTACTGCCACTTACTGCTGAAACATTTAGCAACCGGTACCCC<br>GGTTAGCGATATTGTTCGCGAACCGCTGGTTCGTTTATGCCGTCAGATGACTTGTAGCGCCGCATTCGATCTGATGCATGATTATCTGACAAAAAGCAGTGGCTATATTAAACACACCTTTTTAAGCTGGGATTAC<br>GTGCCGAAAGAAAAGAGCCGTTTAAAGGTTTATAGCGGCAGCACCAGTATCTCTTTAGAAAAGGCAGAAGAAGTTTGGAGCTTAGGTGGTCGTGTTCAAGGTGAGGAAATTAGCGAGGGCATCAAGCTGATTA<br>AGCAGCTGTGGAAGACTTTAGGTCTGAAGAGCTGCACCGATAATACCGCCCAGCCGGTGGAACTGGAGTTCGATGTGAATGAAGAAGCCCAGCAGAAATTTCAACCGCCGCTGACTTGGAACTATGAACTGCAG<br>CCGGGTATTAGCCACCCGCTGACCAAGCTGTATTTCCCGGTGCATGGCATGAACGATTTAAAGGTGGCCGAGGCTTTAAGCAAATTTTTTCATTTTCTGGGTTGGCATGACCAAGCTGAAAGCTACTTAAGTGTGC<br>TGCGTGATTTATATCCGGAAGAGGACATCAGCAAGTGCACACAGCTGCAGAGCTGGATTTCTTTAGCCTATACCAGCCAGAAAGGCGCCTATATTAGCGTGTACTACCGCAGCGCCAAACGCAGCTTATAATGA<br>CTCGAG |
| CdpC3PT | CCATGGCCACAGTGAGCAGTACCGCAGTTGAAGCCAGCGCACCGTGTGCCGAGATGGGCCATGATATCCCGTATCGTACTTTAAGCCGCAGCATGATTTTTGCCAATTTAGATCAGTACCAGTATTGGCACCAG<br>ATCGGCCCGGTGCTGGGCAAAATGCTGGTGGACGGCGAGTATAGCATCCACCGCCAGTATGAGTATTTATCTTTATTTGCCCATCTGATCATTCCGAAGCTGGGCCCGTTCCCTAGTCCGGGTCGTGACATCTAT<br>CGTTGTTTACTGGGCAATATCGGTGGTCCGTTCGAACTGAGCCAGAATTTCCAGCGTCTGGGTAGCACAGCACGTCTGGCCTTTGAGCCTACCAGTTATCTGGCAAGCACAAGCGCCGATCCGTTTAACCGTCAC<br>GCAGTTCATGCAACTTTAGCCGAGTTACGTATGACCGGTAGCGCCACCGTGGATTTAGAGCTGCATCATGCACTGGCAGCCGATTTAACTTTAACCGACCGCAATGAACAGCTGCTGACCGCCGAACTGGCCAA<br>AACCAACTGGAAAAGCCAGATTTTACTGGCTTTAGATCTGAACAAGACCGGCATCACCGTGAAAGAGTACTTCTACCCGGCCTTAAAAGCCGCCGCAACCGGCCATAGTGTTGCAGAGCTGTGTTTCAGTGCCA<br>TTCGCAAGGTTGACGTGCAAGGTCGTCTGGCAGCACCGTGTAAAGCCATTGAAGCCCATATGCAGCGCCAGACCCAAACAGATATCCACTTTTTAAGCGTGGATCTGGTTGAACCGGGTACAACCCGCTTCAAG<br>CTGTATCTGATGGAACTGGAGGTGACTTTAGCCAAACTGGAAGAACATTGGACTTTAGGCGGCACTTTAACCGATAAGGAAACCATGCGTGGTCTGCAGATGATCCGCGAACTGTGGGTGGATCTGGAGATCGT<br>GGACGGTAAACGTGCAGAGCCGCAGCGTCCGAGCTTACCGGGTGACCCGCTGAGCATCGTGCCGTTTTTTATGAATTATGAAATTACCCCGGGCCAGCCGCTGCCGAAACCTAAATTTTATTTTCCGCTGATCG<br>GTATTCCGGAGCTGAAGATCGCAAACGTTCTGGCCGCCTTCTTTGAACGTCATGGTATGCACGATCTGGCCCGCGTGTATCCGGAAAATTTACAGAGCTACTATCCGGGCGAAGATCTGGCCATTGCCACCGATC<br>GCCAAGCTTGGCTGAGCATCAGTTACACCGAGGAGAAAGGCCCGTATTTAACCATGTACTATCATTAATGACTCGAG |

†The *Nco*I and *Xho*I cloning sites are highlighted in yellow and cyan, respectively; the stop codons are highlighted in green.

**Supplementary Table S1** (continued).

| PT | Sequence of synthetic gene† |
|---|---|
| CdpC7PT | CCATGGCCGCAAACCGCGTGAGCAATGACCCGGACGCCGTTAGCCAGAATGAAGTTCAAGCTGCCAAACGTCCGGACATTGCCCAGAACACCGACAATGTGGACAGCACCCCGTATCGCATTCTGACCCAGTT<br>TCTGACCTTTCCGGACGAAGCACAGAAGGGTTGGTGGGAAGATAGCGGCAGTTTACTGAGCCGTTTACTGCAAGTTGCCCGTTATGACGTGCATGATCAGTACGCAACTTTACTGTTTCTGTACAAGCATTTAAT<br>TCCGTTTATGGGCCCGTACCCGCAGCGTCGCCGCTGCGTTTTCGAACCGTACGGCGCCCCTATTGAATATAGCATTAATTTTCAAGAAAATAAGAAAGAGATTTTACGCATCGTGCTGGATCTGGTTAGCATCCC<br>GTATTGCACCAAGGACAACCCGAGCCATCTGGATTGCGTTGCCGCATTTGAGAGCACTTTAGGCCAAATCAGCCCGAAAGTGTACAATCGCGATTTATATAAGCATTTCGCAGACGATCTGCTGCCGACCAAAA<br>AGGACGACGAAGAACTGACTTTACACGAAATGATCACCGACAGCAACTCTTTACCGGCAGTGGCACTGTGGGGCTTTGTGCTGGAAGAAGGCGGTGATGTGACAATTAAAGGTTATATTAATCCGCAGCCGAA<br>TGCAAAAGCCGTTGGTGCCAGCCCGCGTGAACGTTTAAATGAGAGTCTGCAGAAGCTGGACAGCATGGTGGACTATAGCGATTCTTTAAAGCTGGTGCGCGAATACACAATGGAAGGCAGTTGGCACACCGG<br>CGATCAGCTGGTGAGCTGGGACTATGGTCCGCCGGAGGTTAGCCGCATTAAAATGTATGGTGCCGCCCCGGAAAACGTTAGCCCGGCCACCATTCGTGATGTTTGGACTTTAGGCGGTCGCGTTGACAAAGAA<br>ACCAGCCGTAAGGGTTTAGAACTGGCCATCAAACTGTGGGAGCTGATTCACATGCAGATGGAGAGCCCGCCGATGGACCGCAAACGTGAGTTTTTAATGCATGGCATGCTGTGGCATTATGAGGTGTGGCCGG<br>GTGCCCAGTACCCCGTTCCGAAGATTTATTTACCGGCAGCTGGTACAAACGACGGCCGTGTTGCCGAGACAATCAGCAAATTCTTCTATTCTTTAGGCTGGAAAGAGCGCGCAGAGAGCTACCCTCAGATGCTG<br>AAGGACATCTTTCCGGACGTGGATCTGAGCCAGAGTAGTCGTCTGCAGACATGGATTAGCTTTAGTTATACCGAGCAAGGTGGCGCATATAGCACCGTGTACTACCAAGCTGCCACCCGTAGCACCGAATTTC<br>TGGCAGAATAATGACTCGAG |
| CdpNPT | CCATGGCCGATGGCGAAATGACCGCCAGTCCGCCGGATATTAGCGCTTGTGACACCAGTGCAGTTGACGAACAGACTGGTCAAAGTGGCCAGAGCCAAGCTCCGATCCCGAAAGATATCGCCTATCACACTTTA<br>ACCAAAGCTTTACTGTTCCCGGACATTGACCAGTATCAGCATTGGCACCACGTGGCACCTATGCTGGCAAAGATGCTGGTTGATGGCAAGTACAGCATCCACCAGCAGTACGAATATTTATGTTTATTCGCACAG<br>CTGGTGGCCCCCGTTCTGGGCCCTTACCCGAGTCCGGGTCGTGATGTGTATCGCTGTACTTTAGGTGGCAACATGACAGTGGAACTGAGCCAAAACTTCCAACGTAGCGGTAGCACAACCCGCATCGCCTTTGAA<br>CCGGTGCGCTATCAAGCTAGTGTTGGCCACGATCGTTTCAATCGCACCAGCGTGAACGCCTTCTTTAGCCAGCTGCAGCTGCTGGTGAAAAGCGTGAACATCGAGCTGCATCATCTGCTGAGCGAACATCTGACT<br>TTAACCGCCAAAGATGAACGCAATTTAAACGAAGAACAGTTAACCAAATATCTGACCAACTTTCAAGTTAAGACCCAATATGTGGTGGCTTTAGACTTACGTAAGACCGGCATTGTGGCCAAAGAATACTTCTTT<br>CCCGGTATTAAATGCGCCGCCACCGGTCAGACCGGCAGCAATGCATGCTTTGGCGCCATCCGCGCAGTTGACAAAGATGGCCATCTGGATAGTCTGTGCCAGCTGATCGAGGCCCATTTTCAACAAAGCAAAATT<br>GATGATGCATTTTTATGCTGTGATTTAGTGGATCCGGCACATACCCGCTTTAAAGTTTATATTGCAGACCCGCTGGTGACACTGGCCCGCGCCGAAGAACACTGGACTTTAGGTGGCCGTCTGACAGATGAAGAT<br>GCAGCCGTGGGTCTGGAAATCATCCGCGGTCTGTGGAGCGAACTGGGTATCATCCAAGGTCCGCTGGAGCCGAGTGCCATGATGGAGAAAGGTTTACTGCCGATTATGCTGAATTATGAGATGAAAGCCGGTCA<br>GCGCTTACCGAAGCCGAAACTGTATATGCCGCTGACTGGTATCCCGGAAACCAAAATTGCCCGCATCATGACCGCCTTCTTCCAGCGCCATGACATGCCGGAGCAAGCTGAGGTGTTTATGGAGAATCTGCAAGC<br>CTACTACGAGGGTAAAAATTTAGAAGAAGCTACACGCTACCAAGCTTGGCTGAGCTTTGCCTACACAAAGGAGAAGGGCCCGTATTTAAGCATTTACTACTTTTGGCCGGAATAATGACTCGAG |
| CTrpPT | CCATGGCAACCACCTATACTTTAAGTAAGGGCAGCGATCGCCCGCAAACAAGTGGCGATACCGATGGTCCGCAGCCGTATGATGTGCTGAGCAAATATTTACGTTTCCCGGACTTTGACCAAGATCAGTGGTGG<br>CAGAATACAGCACCGATGCTGGGCAAGCTGCTGAGCCAGTGCAAATACAATGTGCACCAACAGTATCAGCATTTATGTTTATATGGTTTACATGTGATTCCGTTTCTGGGTCCGTGGCCGGACATCCAGCGCAGT<br>AAGCTGTACAAGAGCGTGTTAAGTGGTTTAGGTTCTTTAGAATTTAGCCAGAACTTCACCAAGACCGGTAAAACCGTGCGTATGGGCTTTGAGCCGACAACCTTTATCGCCAGTACCAGTCGTGACGTGTGTAAC<br>CGCCACTGTTTAGGTAAAGTGCTGAACCGCTTTAAGCAGCTGGACATTAAATTAGATCTGCAACTGTATCATCAGCTGGTGAACGAGGTGAGTCTGACCGATCAAGAAGAAAATACTTTACTGGAACGTGCCATT<br>CTGGATGATGAACCGGCAAAGAGTCAGTCTTTACTGGCTTTAGATTTCAATAAAGACGACGTTACCGTGAAACTGTATCTGTACCCGCAGCTGAAGTCTTTAGCCACTGGTATCCCGCGTACCCAGCTGATGTTC<br>AGCGCAGTGCGCAACGTGGATAAAACCCGCGCATTTAGCGAGAGCATGAACATGATCGAAGAATATTTCACCAGCGTGCGCGCCAACGCAGCACCGTATTGGATTAGCTGCGATCTGGTGGAACCGAATAAAA<br>CCCGCTTCAAAATCTATATTGCTTTATTCCAAGTTAACTTTGAGAACGCCGTGAGTATGTGGACTTTAGGTAACCGCATTACCGACCCGGAAACCATGCGCGGTCTGGCAATGGTGCGCGATTTATGGAACAGCT<br>TTGATATCCAAGATGGTTTACGTGAACAGAAAAACCGTCCCGGTAATCCGGGCGATCCGAGCAACATCGTGCCGATGCTGTTTAATTTAGAGATTCTGCCGGGCAAAGCCTATCCGCAGCCGAAAATCTACTTTC<br>CGACCACCGGCATGAACGATCTGGACGTGGCAAAGGTGATGGTGGAGTTTTTAAGCGTCACGGTTTACACGAGCATGCCCAGAGCTATATCGACAATTTAGCCAGTTACGTGCCGCACATGGATTTAAACGAT<br>TGTACCGATCTGCAAGCTTGGATTAGTTTCAGCTACAGTGACGCCACCGGCCCGTATATCACCGTTTACTATCATTAATGACTCGAG |
| EchPT1 | CCATGGCACCGAGCGAGGTGCTGACCAGCTATTACGATTACCCGACCCATGACCAAGAAGCATGGTGGCGTGATACCGGTCCTCTGTTTGGCCGCTTTCTGAAAGGCGCTGGTTACGACGTTCACACACAGTAC<br>CAGTACTTAGTGTTTTTATCAAGAATATTCTGCCGAGTCTGGGTCCGTATCCGGCCCGTTGGCGTAGTACCATCACCCCGACTGGTTTACCGATCGAATACTCTTTAAACTTCCAGCTGAACAGTCGTCCGTTAC<br>TGCGCATCGGTTTCGAACCGTTAAGCCGCTTTAGTGGCACCCCGCAAGATCCGTACAATAAGATTGCAGCCGCCGATCTGCTGAATCAGCTGAGCAAGCTGCAGCTGCATGAGTTTGACACACAGCTGTTTAAT<br>CATTTCACCAACGAGTTTGAACTGAGCAAGAGCGAGAGCGAATCTTTACAGAAGCAAGGTGGCATTAATGGCAAGAGCACAGTGCGTAGCCAGACCGCATTCGGTTTTGATCTGAAAGGCGGTCGTGTGGCAG<br>TGAAAGGCTATGCATTTGCCGGTTTAAAAAATCGCGCAACCGGCACCCCCGTTGGTCAGCTGATCAGCAACAGCATCCGCAATTTAGAGCCGCAGATGCATTGCTGGGATAGCTTTAGCATTTTAAATAGCTAC<br>ATGGAAGAGAGCGATGGCTGGAACGAGTACAGTTTCGTGAGCTGGGATTGCGTGGATATCGAACGCAGCCGTCTGAAACTGTACGGTGTGCATAACGCCGTGACTTGGGATAAGGTGAAGGAAATGTGGACA<br>CTGGGCGGCCGTATTGAAAACAACGCCACCATCAAGACCGGTTTAGAACTGCTGCAGCACATGTGGTCTTTACTGCAGATTAACGAGGGCGATCGCGACTATAAGGGTGGTTTTGCAGCCGACAACGGCGGCA<br>AAACTTTACCGATTATTTGGAATTATGAACTGAACAAAGGTAGCCCGCATCCGGCCCCGAAATTTTATTTTCCGGTGCACGGCGAAAACGATCTGCAAGTTAGCAAGAGTATCAGCGAATTTTTCACCCATTTA<br>GGCTGGCAAGATCATGCACGCCAGTATCCGCATCTGCTGCGTCAGATCTACCCGAATCAAAACATTAGCCAGACCGAGCGTTTACAAGCTTGGATTAGCTTTGCCTACAACGAACGCACCGGCCCGTATCTGAG<br>CGTGTATTATTACAGCGCCGAACGCCCGCCTTGGGGTAGTGATCAAGTTAAATAATGACTCGAG |

†The *Nco*I and *Xho*I cloning sites are highlighted in yellow and cyan, respectively; the stop codons are highlighted in green.

**Supplementary Table S1** (continued).

| PT | Sequence of synthetic gene† |
|---|---|
| FtmPT1 | CCATGGCACCGCCGGCACCTCCGGATCAGAAACCGTGTCATCAGCTGCAGCCGGCACCGTACCGTGCACTGAGCGAAAGCATTTTATTTGGCAGCGTGGATGAGGAACGCTGGTGGCATAGCACCGCACCGA<br>TTCTGAGCCGTCTGCTGATCAGCAGCAACTATGATGTGGACGTGCAGTACAAATATTTATCTTTATACCGTCATTTAGTGCTGCCCGCTCTGGGTCCTTATCCGCAGCGTGATCCGGAAACTGGTATTATCG<br>CCACCCAGTGGCGTAGTGGTATGGTGCTGACCGGTCTGCCGATTGAGTTTAGCAACAACGTGGCACGTGCTTTAATTCGTATTGGCGTGGATCCCGTTACCGCCGATAGCGGTACCGCACAAGATCCGTTTA<br>ATACCACCCGTCCGAAGGTGTATTTAGAGACAGCCGCACGTCTGCTGCCGGGTGTGGATTTAACCCGCTTTTACGAATTTGAAACCGAGCTGGTTATCACCAAAGCCGAGGAAGCAGTTCTGCAAGCTAATC<br>CGGACTTATTCCGCAGTCCGTGGAAAAGCCAGATTCTGACCGCTATGGATTTACAGAAGAGCGGCACCGTGCTGGTGAAAGCCTACTTCTATCCGCAGCCGAAAAGTGCCGTGACCGGCCGTAGCACCGAGG<br>ATCTGCTGGTGAATGCAATCCGCAAAGTGGATCGCGAGGGTCGCTTTGAAACCCAGCTGGCCAATTTACAACGTTACATTGAGCGTCGTCGCCGTGGTCTGCATGTGCCGGGCGTTACAGCCGATAAACCGC<br>CGGCAACCGCAGCCGATAAGGCCTTCGACGCTTGTAGTTTCTTCCCGCATTTTCTGAGCACCGATTTAGTGGAGCCGGGCAAAAGCCGTGTGAAGTTCTACGCCAGTGAGCGCCATGTGAATCTGCAGATGG<br>TTGAGGATATCTGGACCTTCGGCGGTTTACGCCGTGACCCGGATGCTTTACGCGGTTTAGAGCTGCTGCGCCACTTCTGGGCCGACATTCAAATGCGCGAAGGTTATTACACCATGCCGCGCGGTTTTTGCG<br>AACTGGGTAAGAGCAGCGCCGGTTTCGAAGCCCCGATGATGTTCCACTTCCATCTGGATGGCAGTCAGAGCCCTTTTCCGGATCCGCAGATGTATGTGTGCGTTTTCGGCATGAACAGCCGTAAGCTGGTTG<br>AAGGTCTGACCACATTTTATCGCCGCGTTGGCTGGGAAGAAATGGCCAGTCATTACCAAGGTAACTTTTTAGCCAATTACCCGGACGAGGACTTTGAGAAAGCAGCCCATCTGTGCGCATACGTGAGCTTTG<br>CATACAAGAACGGCGGTGCCTACGTTACTTTATACAATCATAGCTTCAATCCGGTGGGCGACGTGAGCTTCCCGAATTAATGACTCGAG |
| NotF | CCATGGCAACCGCCCCGGAACTGCGCGTTGACACCTTTCGTGCACCCGAAGATGCACCGAAAGAACCGAGCGCACAGCAGCCGCGCTTACCGAGTAGTCCGAGTCCCGCTCAAGCTTTAGCCAGCTATCATCA<br>TTTCCCGACCAACGATCAAGAACGCTGGTGGGAAGAAACCGGTTCTTTATTTAGCCGCTTTCTGGAGGCCGGCCAGTATGGTCTGCCTCAGCAGTACCAGTTTATGTTTTTCTTCATGCACCACTTAATTCCGGC<br>TTTAGGTCCTTACCCGCAGAAATGGCGCAGCACCATTAGCCGCAGTGGTTTACCGATTGAGTTCTCTTTAAACTTCCAGAAGGGCAGTCATCGTCTGCTGCGCATCGGCTTCGAACCGGTGAGTTTTTAAGTGG<br>CAGCAGTCAAGATCCGTTCAACCGCATCCCGATTACCGATCTGCTGAATCGTTTAAGTAAGCTGCAACTGAGCAACTTCGACACCCCGTTTTTCCAGCATTTACTGAGCAAATTCCAGCTGAGTCTGAGCGAAG<br>TGCGCCAGTTACAAAAACAAGGTAGCGGCCCCGATGCACATCCGTTAAAGAGCCAAGCCGCCTTCGGCTTCGACTTCAATCCGGATGGTGCCATTTTAGTGAAAGGCTACGTGTTCCCGTATCTGAAAGCCAAA<br>GCCGCCGACGTTCCCGTTGGTACTTTAATTGCAGAGGCCGTGCGCACCATCGACGTTGAACGCAACCAGTTTACCCACGCCTTCGGTTTAATCAACGACTATATGCAAGAAAGCACCGGCTACAACGAGTATAC<br>CTTTTTAAGCTGCGATTTCGTGGAGACAAGCGAGCAGCGTCTGAAGATTTACGGTGCCCATACCGAGGTTACTTGGGCCAAAATTGCCGAGATGTGGACTTTAGGCGGTCGTTTAATCGAAGAGCCGGAGATCA<br>TCGCTGGTTTAGCACGTTTAAAACAGATTTGGAGTCTGCTGCAGATTGGCGAAGGCAGCCGCGCCTTTAAAGGCGGTTTCGATTACGACAAGAGCAGCGCAACCGACCAGATTGCCAGCCCGATCATCTGGAAT<br>TACGAAATTCACCCGGGTAGCCGCTTTCCGGTGCCGAAATTCTATTTACCGGTGCATGGCGAGAACGATTTACATGTTGCACGCGCACTGGCACAGTTCTGGGATTCTTTAGGTTGGCCGGAGCATGCATGTGCC<br>TATCCGGATACTTTACAGCAGCTGTATCCGGATCAAGATATCAGCCAGACCACACGTTTACAAAGCTGGATCAGCTACAGCTATACCGCCAAACGCGGCGTTTATATGAGCGTGTACTATCACAGCCAGAGCAC<br>CTATTTATGGGAAGAGGATTAATGACTCGAG |
| RoqD | CCATGGCAACCGTTAGCAGCACCGTTCAACCGCTGAAAGCTTGTGTGCCGGAAGCAACCGAGGCAGCAGATATGCCGCATCACACTTTAAGCAAAAGCATGACCTTCGCAAATTTAGACCAGTATCAATATTG<br>GCATGCCGTGGGTCCGATGCTGGGCCGTATGCTGAGCAACGGCAGTTACAGCATCCATAAGCAATATGAGTATCTGTGTTTATTCGCCCACGTGATCATTCCGAAGCTGGGCCCGTTTCCGGGTGGCCGCGATAT<br>TTACAAATGTCTGCTGGGCGGCACCGGTAGCGTTGAACTGAGCCAGAACGTGCAGAAGCTGGGTCTGACCGCCCCGCGTTGCCTTTGAGCCTACCAGTTACATCGCCAGCACCGGTGTTGATCCGTTAAATCGC<br>CACACCGTGCATGCCACTTTAGTGGAATTACGTGCCATTGGCAGCGCCAGTATCGATATGGAGCTGCACCAAATGCTGGTGAACGAGCTGACTTTAACCGACCGCGAGGAACGTCTGATGAGCCCGGAAGCC<br>ATTAGCGGCACCGCATGGAAAACCCAGATTTTACTGGCTTTAGATTTAGGTCAGACCGGCATCACCATCAAAGAGTATTTTTATCCGGCTTTAAAAGCCAGCGTTACCGGCCAAAGCGTGGCCAAGCTGTGCT<br>TCAGTGCCATTCGCAAAGTGGATAAGCAAGGTCGCTTTGAACCGGCAAGTAAGGCCATTGAAACCTATATGAAGACCCAGAGCCAGACCGATCTGTACTTTCTGAGCTGTGATTTAGTGGACCCGGCAGCAA<br>CACGTATTAAACTGTATTTAATGGAACTGGATATGCGTTTAGCCAAAGTGGAGGAACACTGGACTATGGGTGGCAAACTGAATGACGAGGAAACACTGCTGGGTCTGAAGATGCTGCAAGAACTGTGGGTGG<br>AGTTTGGCATCATTGAAGGCATGCGTAATGAACCGGAACGCCCTAGTCTGCCGGGTGATCCGGATACCATCGTGCCGTTCATCATGAACTATGAGATGAGCCCGGGCGAGGCACTGCCGAAACCGAAGTTCT<br>ATTTCCCGCTGGTTGGCATCCCGGAGCTGAAGATCGCCAATGTGTTAACCGCCTTCTTTGAACGCTACGGCATGCCGGAACAAGCTGCCGTGTATCGCAATAATTTACAAACACCGAGCAAAGATTTAGTTAT<br>TGCCACAGACCACCAAGCTTGGCTGAGCTTCAGCTACACCAAGAAGAAGGGTCCGTATTTAACAATGTATTATCATTAATGACTCGAG |

†The *Nco*I and *Xho*I cloning sites are highlighted in yellow and cyan, respectively; the stop codons are highlighted in green.

**Supplementary Table S2.** Sequence data of recombinant PTs.

| PT | Sequence of expressed recombinant PT | MW (kDa) |
|---|---|---|
| AnaPT | MASPLSMQTDSVQGTAENKSLETNGTSNDQQLPWKVLGKSLGLPTIEQEQYWLNTAPYFNNLLIQCGYDVHQQYQYLAFYHRHVLPVLGPFIRSSAEAN<br>YISGFSAEGYPMELSVNYQASKATVRLGCEPVGEFAGTSQDPMNQFMTREVLGRLSRLDPTFDLRLFDYFDSQFSLTTSEANLAASKLIKQRRQSKVIAFD<br>LKDGAIIPKAYFFLKGKSLASGIPVQDVAFNAIESIAPKQIESPLRVLRTFVTKLFSKPTVTSDVFILAVDCIVPEKSRIKLYVADSQLSLATLREFWTLGGSV<br>TDSATMKGLEIAEELWRILQYDDAVCSHSNMDQLPLVVNYELSSGSATPKPQLYLPLHGRNDEAMANALTKFWDYLGWKGLAAQYKKDLYANNPCR<br>NLAETTTVQRWVAFSYTESGGAYLTVYFHAVGGMKGNL | 48.9 |
| BrePT | MATAPELRAPAGHPQEPPARSSPAQALSSYHHFPTSDQERWYQETGSLCSRFLEAGQYGLHQQYQFMFFFMHHLIPALGPYPQKWRSTISRSGLPIEFSL<br>NFQKGSHRLLRIGFEPVNFLSGSSQDPFNRIPIADLLAQLARLQLRGFDTQCFQQLLTRFQLSLDEVRQLPPDDQPLKSQGAFGFDFNPDGAILVKGYVF<br>PYLKAKAAGVPVATLIAESVRAIDADRNQFMHAFSLINDYMQESTGYNEYTFLSCDLVEMSRQRVKIYGAHTEVTWAKIAEMWTLGGRLIEEPEIMEGL<br>ARLKQIWSLLQIGEGSRAFKGGFDYGKASATDQIPSPIIWNYEISPGSSFPVPKFYLPVHGENDLRVARSLAQFWDSLGWSEHACAYPDMLQQLYPDLDV<br>SRTSRLQSWISYSYTAKKGVYMSVYFHSQSTYLWEED | 49.6 |
| CdpC2PT | MAAINSRCGLACKRHSESGEAEYTNRVTLSPIEALARYWTSPTEHHTQWQNDTAPLFSRLLEAAQYDVHDQYQYLLFMFGHIIPSLGPYPQRWKSTMTTL<br>GIPLEFSINYQKHGKETVRVALEPVSVVSGTDEDPFNLITTRKFMSDIANLGLESFDMELHDYLINAFFPSANEIRDLQASGYGKRGGEQKSPSAFGFDL<br>KAGGCITVKGYCHLLLKHLATGTPVSDIVREPLVRLCRQMTCSAAFDLMHDYLTKSSGYIKHTFLSWDYVPKEKSRLKVYSGSTSISLEKAEEVWSLGGR<br>VQGEEISEGIKLIKQLWKTLGLKSCTDNTAQPVELEFDVNEEAQQKFQPPLTWNYELQPGISHPLTKLYFPVHGMNDLKVAEALSKFFHFLGWHDQAESY<br>LSVLRDLYPEEDISKCTQLQSWISLAYTSQKGAYISVYYRSAKRSL | 50.6 |
| CdpC3PT | MATVSSTAVEASAPCAEMGHDIPYRTLSRSMIFANLDQYQYWHQIGPVLGKMLVDGEYSIHRQYEYLSLFAHLIIPKLGPFPSPGRDIYRCLLGNIGGPF<br>ELSQNFQRLGSTARLAFEPTSYLASTSADPFNRHAVHATLAELRMTGSATVDLELHHALAADLTLTDRNEQLLTAELAKTNWKSQILLALDLNKTGITVK<br>EYFYPALKAAATGHSVAELCFSAIRKVDVQGRLAAPCKAIEAHMQRQTQTDIHFLSVDLVEPGTTRFKLYLMELEVTLAKLEEHWTLGGTLTDKETMRGL<br>QMIRELWVDLEIVDGKRAEPQRPSLPGDPLSIVPFFMNYEITPGQPLPKPKFYFPLIGIPELKIANVLAAFFERHGMHDLARVYPENLQSYYPGEDLAIA<br>TDRQAWLSISYTEEKGPYLTMYYH | 47.7 |
| CdpC7PT | MAANRVSNDPDAVSQNEVQAAKRPDIAQNTDNVDSTPYRILTQFLTFPDEAQKGWWEDSGSLLSRLLQVARYDVHDQYATLLFLYKHLIPFMGPYPQRR<br>RCVFEPYGAPIEYSINFQENKKEILRIVLDLVSIPYCTKDNPSHLDCVAAFESTLGQISPKVYNRDLYKHFADDLLPTKKDDEELTLHEMITDSNSLPAVA<br>LWGFVLEEGGDVTIKGYINPQPNAKAVGASPRERLNESLQKLDSMVDYSDSLKLVREYTMEGSWHTGDQLVSWDYGPPEVSRIKMYGAAPENVSPATIR<br>DVWTLGGRVDKETSRKGLELAIKLWELIHMQMESPPMDRKREFLMHGMLWHYEVWPGAQYPVPKIYLPAAGTNDGRVAETISKFFYSLGWKERAESYP<br>QMLKDIFPDVDLSQSSRLQTWISFSYTEQGGAYSTVYYQAATRSTEFLAE | 51.1 |
| CdpNPT | MADGEMTASPPDISACDTSAVDEQTGQSGQSQAPIPKDIAYHTLTKALLFPDIDQYQHWHHVAPMLAKMLVDGKYSIHQQYEYLCLFAQLVAPVLGPYP<br>SPGRDVYRCTLGGNMTVELSQNFQRSGSTTRIAFEPVRYQASVGHDRFNRTSVNAFFSQLQLLVKSVNIELHHLLSEHLTLTAKDERNLNEEQLTKYLTNF<br>QVKTQYVVALDLRKTGIVAKEYFFPGIKCAATGQTGSNACFGAIRAVDKDGHLDSLCQLIEAHFQQSKIDDAFLCCDLVDPAHTRFKVYIADPLVTLARA<br>EEHWTLGGRLTDEDAAVGLEIIRGLWSELGIIQGPLEPSAMMEKGLLPIMLNYEMKAGQRLPKPKLYMPLTGIPETKIARIMTAFFQRHDMPEQAEVFME<br>NLQAYYEGKNLEEATRYQAWLSFAYTKEKGPYLSIYYFWPE | 49.8 |
| CTrpPT | MATTYTLSKGSDRPQTSGDTDGPQPYDVLSKYLRFPDFDQDQWWQNTAPMLGKLLSQCKYNVHQQYQHLCLYGLHVIPFLGPWPDIQRSKLYKSVLSG<br>LGSLEFSQNFTKTGKTVRMGFEPTTFIASTSRDVCNRHCLGKVLNRFKQLDIKLDLQLYHQLVNEVSLTDQEENTLLERAILDDEPAKSQSLLALDFNKDD<br>VTVKLYLYPQLKSLATGIPRTQLMFSAVRNVDKTRAFSESMNMIEEYFTSVRANAAPYWISCDLVEPNKTRFKIYIALFQVNFENAVSMWTLGNRITDPET<br>MRGLAMVRDLWNSFDIQDGLREQKNRPGNPGDPSNIVPMLFNLEILPGKAYPQPKIYFPTTGMNDLDVAKVMVEFFKRHGLHEHAQSYIDNLASYVPHM<br>DLNDCTDLQAWISFSYSDATGPYITVYYH | 49.1 |

**Supplementary Table S2** (continued).

| PT | Sequence of expressed recombinant PT | MW (kDa) |
|---|---|---|
| EchPT1 | MAPSEVLTSYYDYPTHDQEAWWRDTGPLFGRFLKGAGYDVHTQYQYLVFFIKNILPSLGPYPARWRSTITPTGLPIEYSLNFQLNSRPLLRIGFEPLSRF<br>SGTPQDPYNKIAAADLLNQLSKLQLHEFDTQLFNHFTNEFELSKSESESLQKQGGINGKSTVRSQTAFGFDLKGGRVAVKGYAFAGLKNRATGTPVGQL<br>ISNSIRNLEPQMHCWDSFSILNSYMEESDGWNEYSFVSWDCVDIERSRLKLYGVHNAVTWDKVKEMWTLGGRIENNATIKTGLELLQHMWSLLQINEG<br>DRDYKGGFAADNGGKTLPIIWNYELNKGSPHPAPKFYFPVHGENDLQVSKSISEFFTHLGWQDHARQYPHLLRQIYPNQNISQTERLQAWISFAYNERT<br>GPYLSVYYYSAERPPWGSDQVK | 48.0 |
| FtmPT1 | MAPPAPPDQKPCHQLQPAPYRALSESILFGSVDEERWWHSTAPILSRLLISSNYDVDVQYKYLSLYRHLVLPALGPYPQRDPETGIIATQWRSGMVLTGL<br>PIEFSNNVARALIRIGVDPVTADSGTAQDPFNTTRPKVYLETAARLLPGVDLTRFYEFETELVITKAEEAVLQANPDLFRSPWKSQILTAMDLQKSGTVL<br>VKAYFYPQPKSAVTGRSTEDLLVNAIRKVDREGRFETQLANLQRYIERRRRGLHVPGVTADKPPATAADKAFDACSFFPHFLSTDLVEPGKSRVKFYASE<br>RHVNLQMVEDIWTFGGLRRDPDALRGLELLRHFWADIQMREGYYTMPRGFCELGKSSAGFEAPMMFHFHLDGSQSPFPDPQMYVCVFGMNSRKLVEG<br>LTTFYRRVGWEEMASHYQGNFLANYPDEDFEKAAHLCAYVSFAYKNGGAYVTLYNHSFNPVGDVSFPN | 52.7 |
| NotF | MATAPELRVDTFRAPEDAPKEPSAQQPRLPSSPSPAQALASYHHFPTNDQERWWEETGSLFSRFLEAGQYGLPQQYQFMFFFMHHLIPALGPYPQKWRST<br>ISRSGLPIEFSLNFQKGSHRLLRIGFEPVSFLSGSSQDPFNRIPITDLLNRLSKLQLSNFDTPFFQHLLSKFQLSLSEVRQLQKQGSGPDAHPLKSQAAF<br>GFDFNPDGAILVKGYVFPYLKAKAADVPVGTLIAEAVRTIDVERNQFTHAFGLINDYMQESTGYNEYTFLSCDFVETSEQRLKIYGAHTEVTWAKIAEMW<br>TLGGRLIEEPEIIAGLARLKQIWSLLQIGEGSRAFKGGFDYDKSSATDQIASPIIWNYEIHPGSRFPVPKFYLPVHGENDLHVARALAQFWDSLGWPEHA<br>CAYPDTLQQLYPDQDISQTTRLQSWISYSYTAKRGVYMSVYYHSQSTYLWEED | 51.7 |
| RoqD | MATVSSTVQPLKACVPEATEAADMPHHTLSKSMTFANLDQYQYWHAVGPMLGRMLSNGSYSIHKQYEYLCLFAHVIIPKLGPFPGGRDIYKCLLGGTGSV<br>ELSQNVQKLGLTARVAFEPTSYIASTGVDPLNRHTVHATLVELRAIGSASIDMELHQMLVNELTLTDREERLMSPEAISGTAWKTQILLALDLGQTGITI<br>KEYFYPALKASVTGQSVAKLCFSAIRKVDKQGRFEPASKAIETYMKTQSQTDLYFLSCDLVDPAATRIKLYLMELDMRLAKVEEHWTMGGKLNDEETLLG<br>LKMLQELWVEFGIIEGMRNEPERPSLPGDPDTIVPFIMNYEMSPGEALPKPKFYFPLVGIPELKIANVLTAFFERYGMPEQAAVYRNNLQTPSKDLVIAT<br>DHQAWLSFSYTKKKGPYLTMYYH | 47.4 |

**Supplementary Table S3.** Identification of recombinant PTs by PMF and peptide sequencing.

| Sample | Best hit with PMF Mascot search | | | Summary of PMF results | | | Summary of peptide sequencing results | | |
|---|---|---|---|---|---|---|---|---|---|
| | Accession number | Name | Organism | Mascot score | Peptide mass values matched | Protein sequence coverage (%) | Identified peptide sequences | Scores (min-max) for sequenced peptides | Protein sequence coverage (%) |
| AnaPT | XP_001258078 | Dimethylallyl tryptophan synthase | *Aspergillus fischeri* NRRL 181 | 103 | 19 | 53 | 8 | 49-139 | 28 |
| BrePT | I4AY86 | Brevianamide F reverse prenyltransferase | *Aspergillus versicolor* | 127 | 22 | 57 | 12 | 43-115 | 38 |
| CdpC3PT | XP_001259405 | Dimethylallyl tryptophan synthase | *Aspergillus fischeri* NRRL 181 | 139 | 23 | 68 | 5 | 56-128 | 20 |
| CdpC7PT | XP_001213396 | Predicted protein | *Aspergillus terreus* NIH2624 | 77 | 20 | 47 | 9 | 59-112 | 34 |
| CdpNPT | ABR14712 | Cyclic dipeptide N-prenyltransferase | *Aspergillus fumigatus* | 162 | 27 | 57 | 6 | 42-91 | 15 |
| CTrpPT | ADI60056 | cyclo-L-Trp-L-Trp prenyltransferase | *Aspergillus oryzae* | 114 | 28 | 53 | 9 | 50-131 | 39 |
| FtmPT1 | AAX56314 | Brevianamide F prenyltransferase | *Aspergillus fumigatus* | 198 | 29 | 62 | 12 | 40-80 | 35 |
| NotF | ADM34132 | Reverse prenyltransferase | *Aspergillus* sp. MF297-2 | 155 | 24 | 59 | 16 | 7-129 | 56 |
| RoqD | XP_002568553 | Pc21g15430 | *Penicillium rubens* Wisconsin 54-1255 | 61 | 17 | 40 | 6 | 32-72 | 23 |

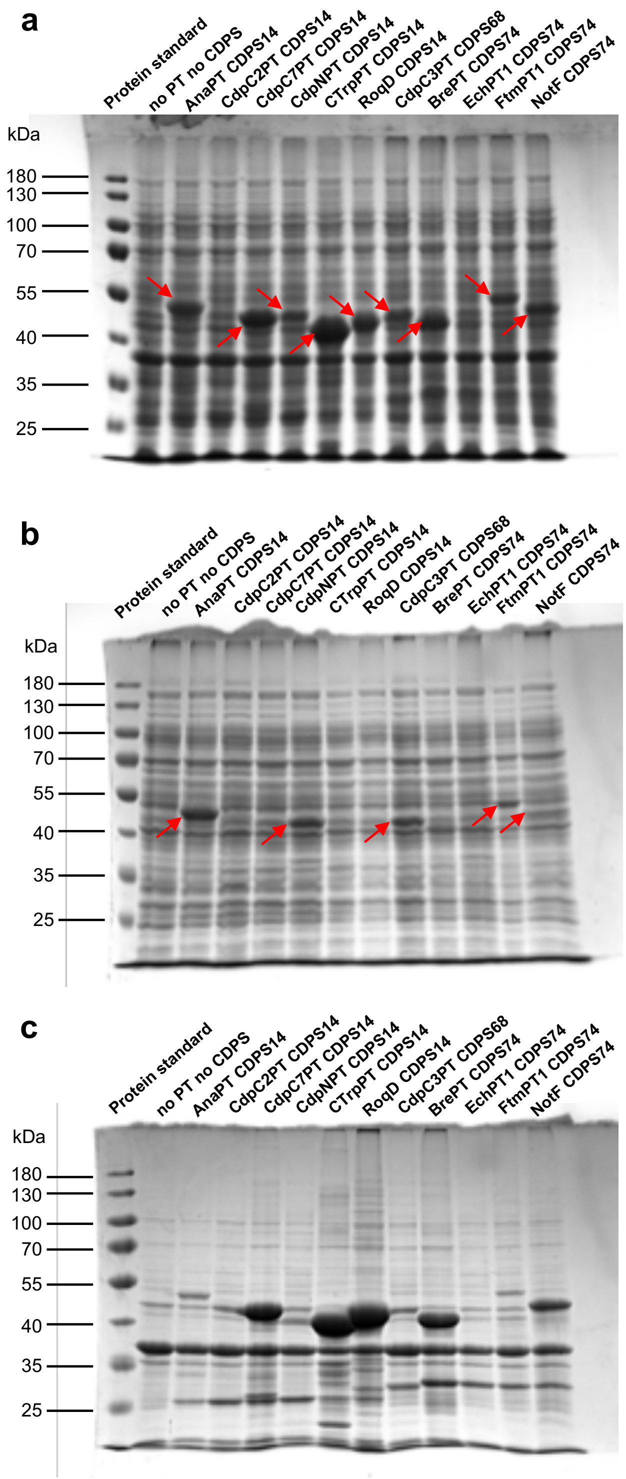


**Supplementary Figure S1**. Analysis of recombinant PT expression by SDS-PAGE. Bacteria BL21AI grown in M9 minimal medium were collected after 48-hour expression of various PTs. Whole-cell fractions (a), soluble fractions (b), and insoluble fractions (c) were prepared and analysed by 12% SDS-PAGE and Coomassie-blue staining. The corresponding overexpressed PT and CDPS are indicated for each lane. The sizes of the proteins of the protein standard (PageRuler$^{TM}$ Prestained Protein Ladder; ThermoScientific) are indicated in kDa. Red arrows in a and b indicate the presence of additional protein bands for which molecular weight corresponds to that of the expected PTs.

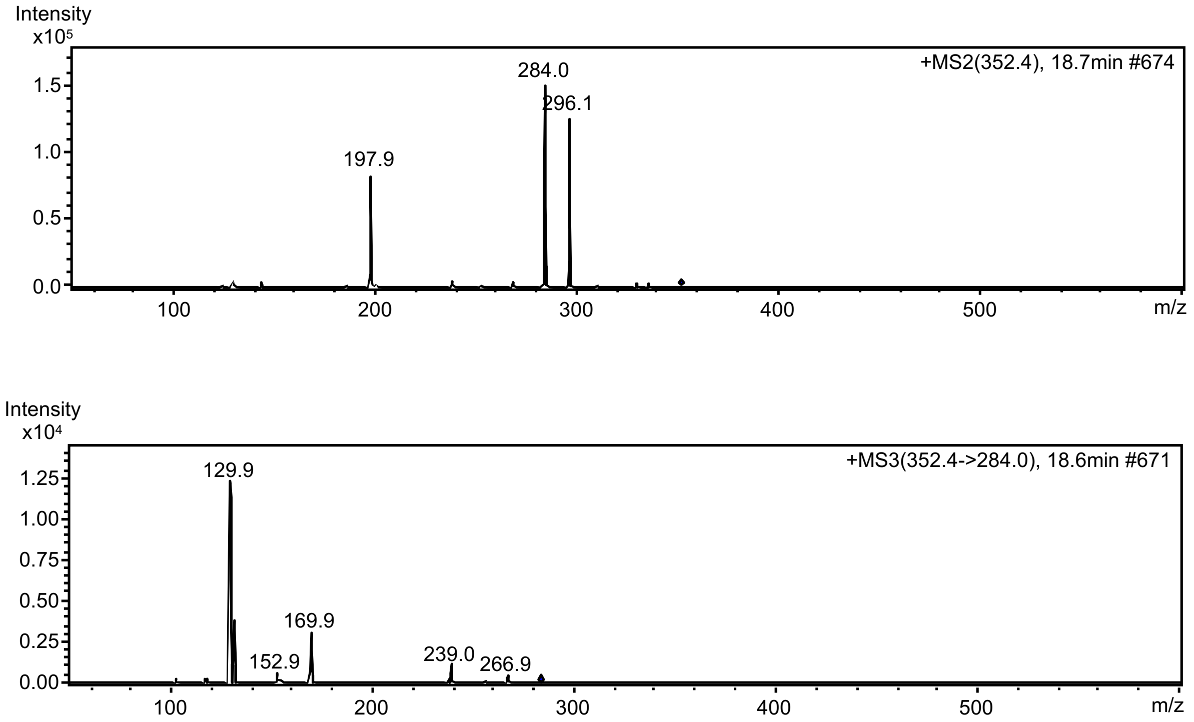


**Supplementary Figure S2.** MS2 and MS3 spectra of prenylated cWP produced by recombinant *E. coli* overexpressing CDPS74 and BrePT without DMAPP pathway engineering.

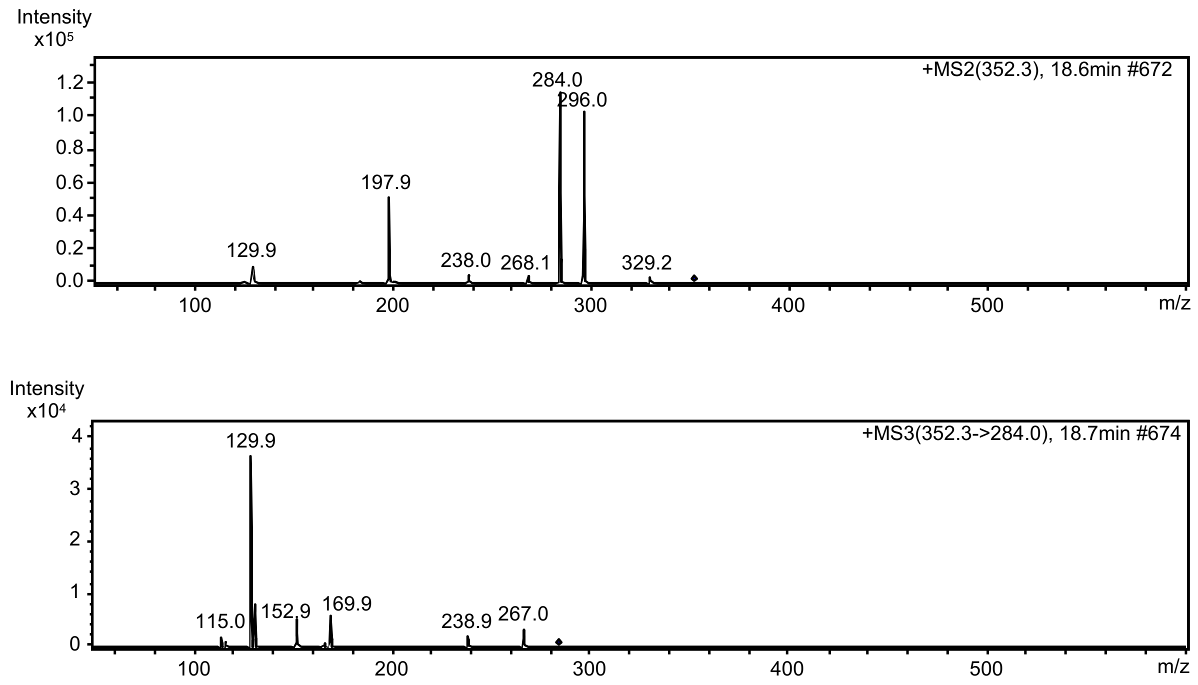


**Supplementary Figure S3**. MS2 and MS3 spectra of prenylated cWP produced by recombinant *E. coli* overexpressing CDPS74 and EchPT1 without DMAPP pathway engineering.

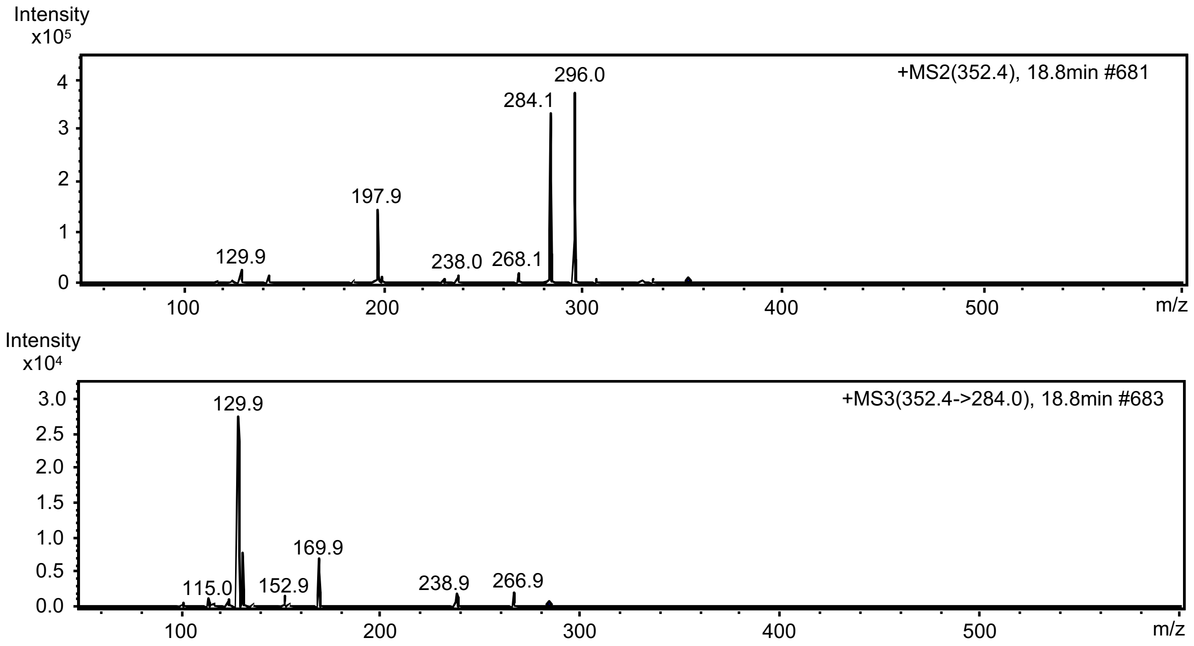


**Supplementary Figure S4.** MS2 and MS3 spectra of prenylated cWP produced by recombinant *E. coli* overexpressing CDPS74 and NotF without DMAPP pathway engineering.

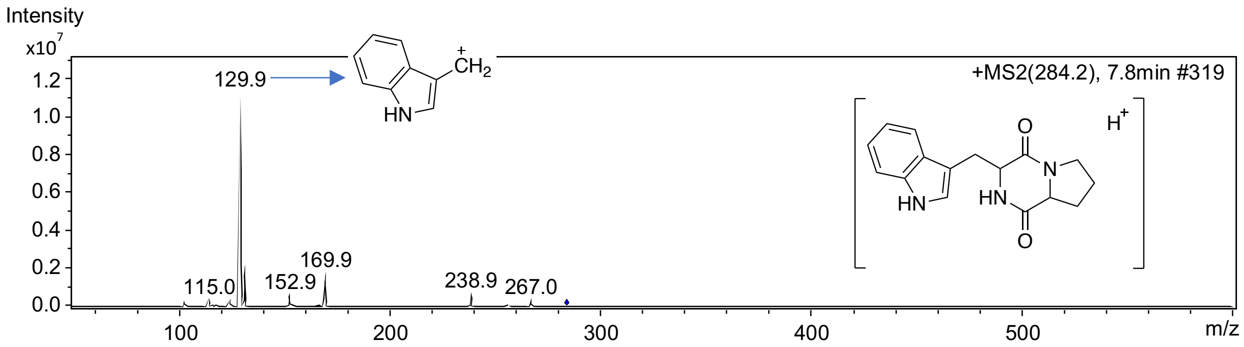


**Supplementary Figure S5**. MS2 spectrum of cWP produced by recombinant *E. coli* expressing CDPS74. The chemical structure of the $MH^+$ ion at m/z 130 characteristic of the indole ring is shown.

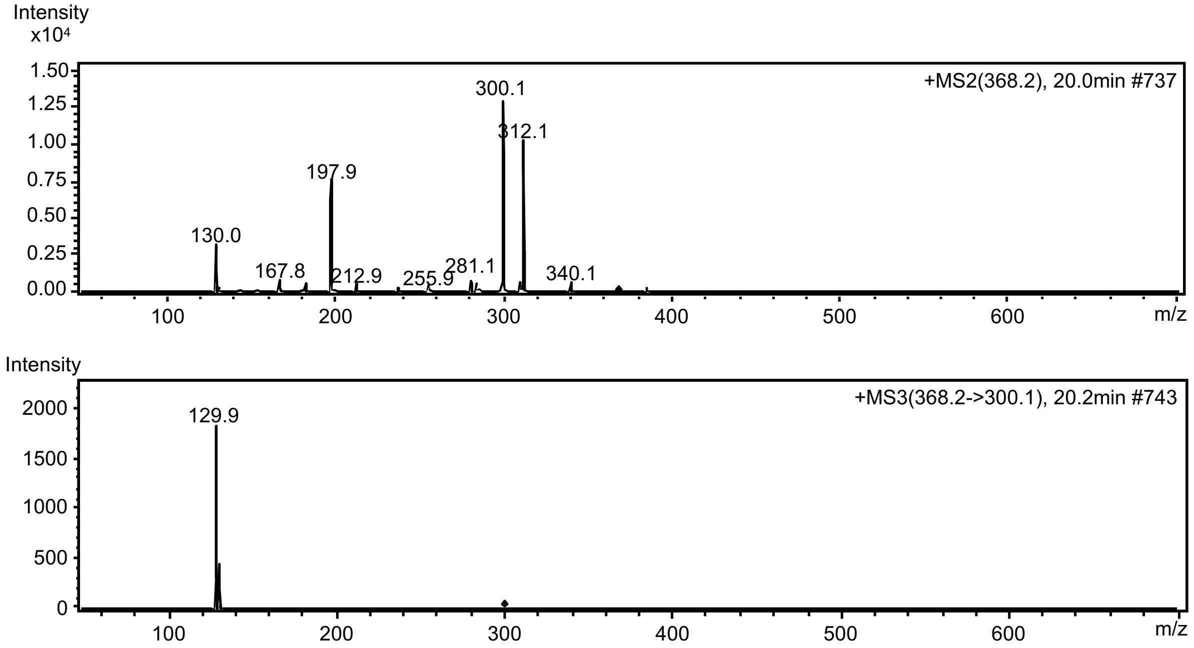


**Supplementary Figure S6.** MS2 and MS3 spectra of the compound produced by recombinant *E. coli* expressing CDPS68 and CdpC3PT with a retention time of 20.1 min in Fig. 2b.

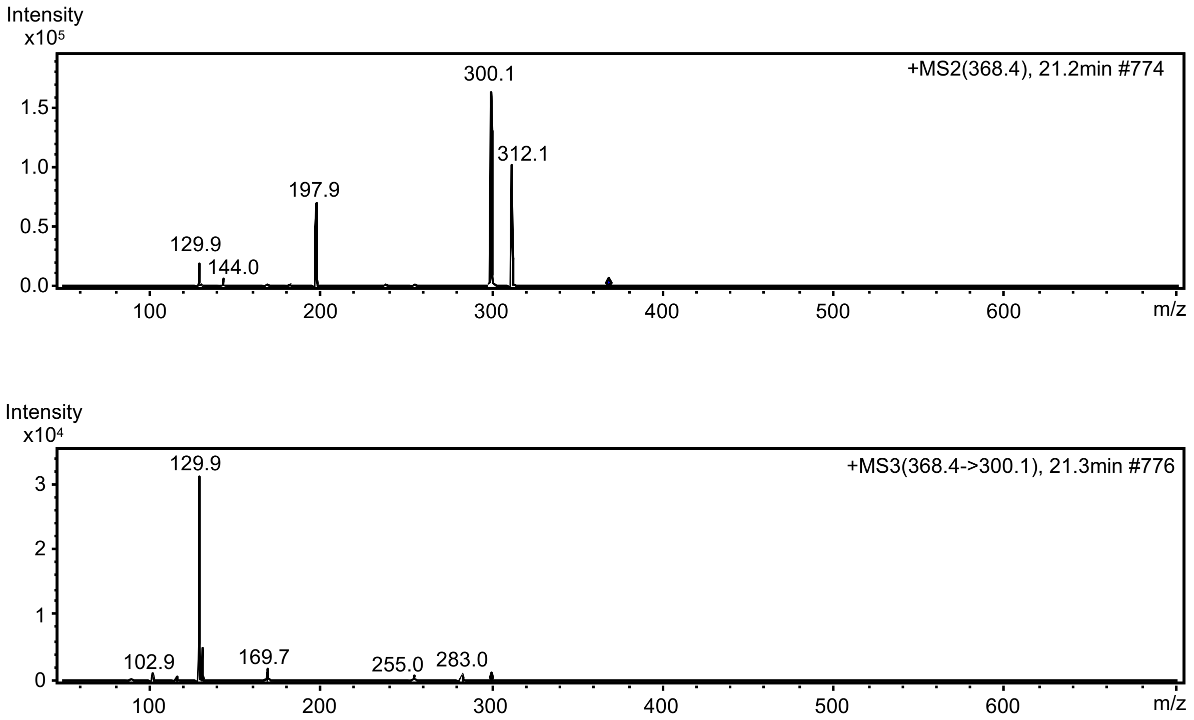


**Supplementary Figure S7.** MS2 and MS3 spectra of the compound produced by recombinant *E. coli* expressing CDPS68 and CdpC3PT with a retention time of 21.2 min in Fig. 2b.

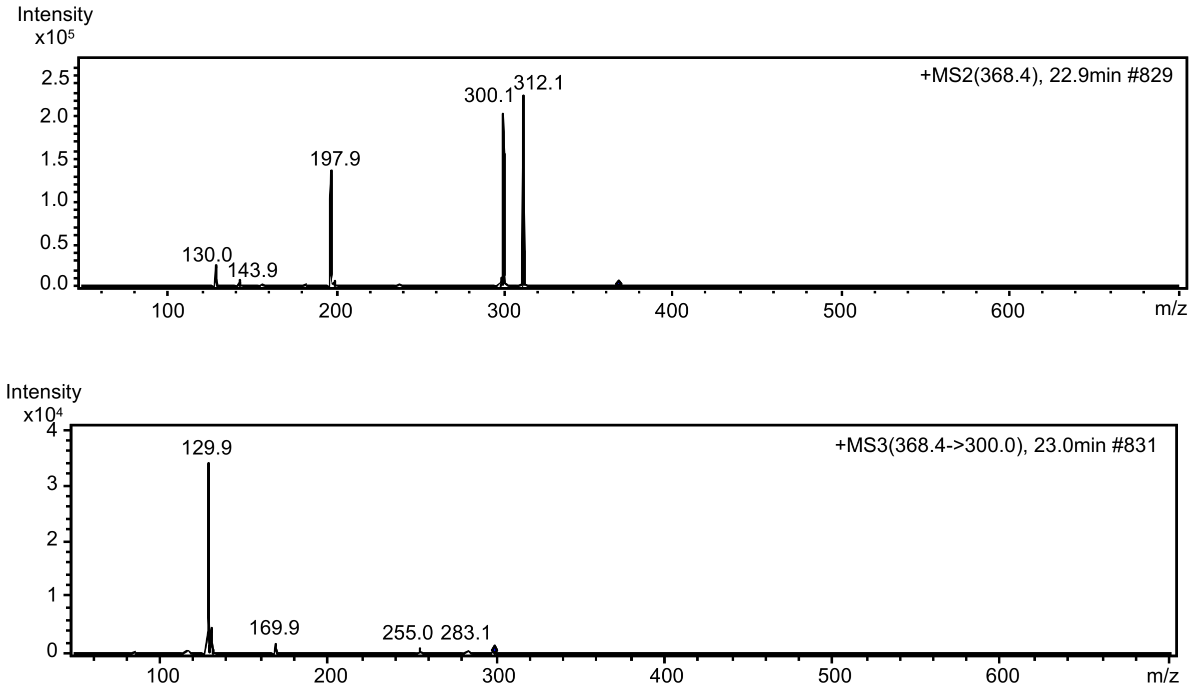


**Supplementary Figure S8.** MS2 and MS3 spectra of the compound produced by recombinant *E. coli* expressing CDPS68 and CdpC3PT with a retention time of 22.9 min in Fig. 2b.

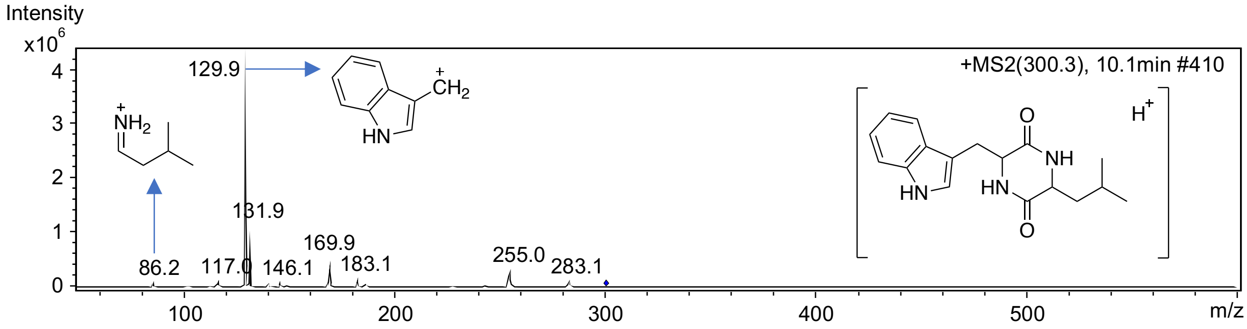


**Supplementary Figure S9.** MS2 spectrum of cWL produced by recombinant *E. coli* expressing CDPS68. The chemical structure of the $MH^+$ ion at m/z 130 characteristic of the indole ring is shown. The chemical structure of the immonium ion characteristic of leucine at m/z 86 is also shown.

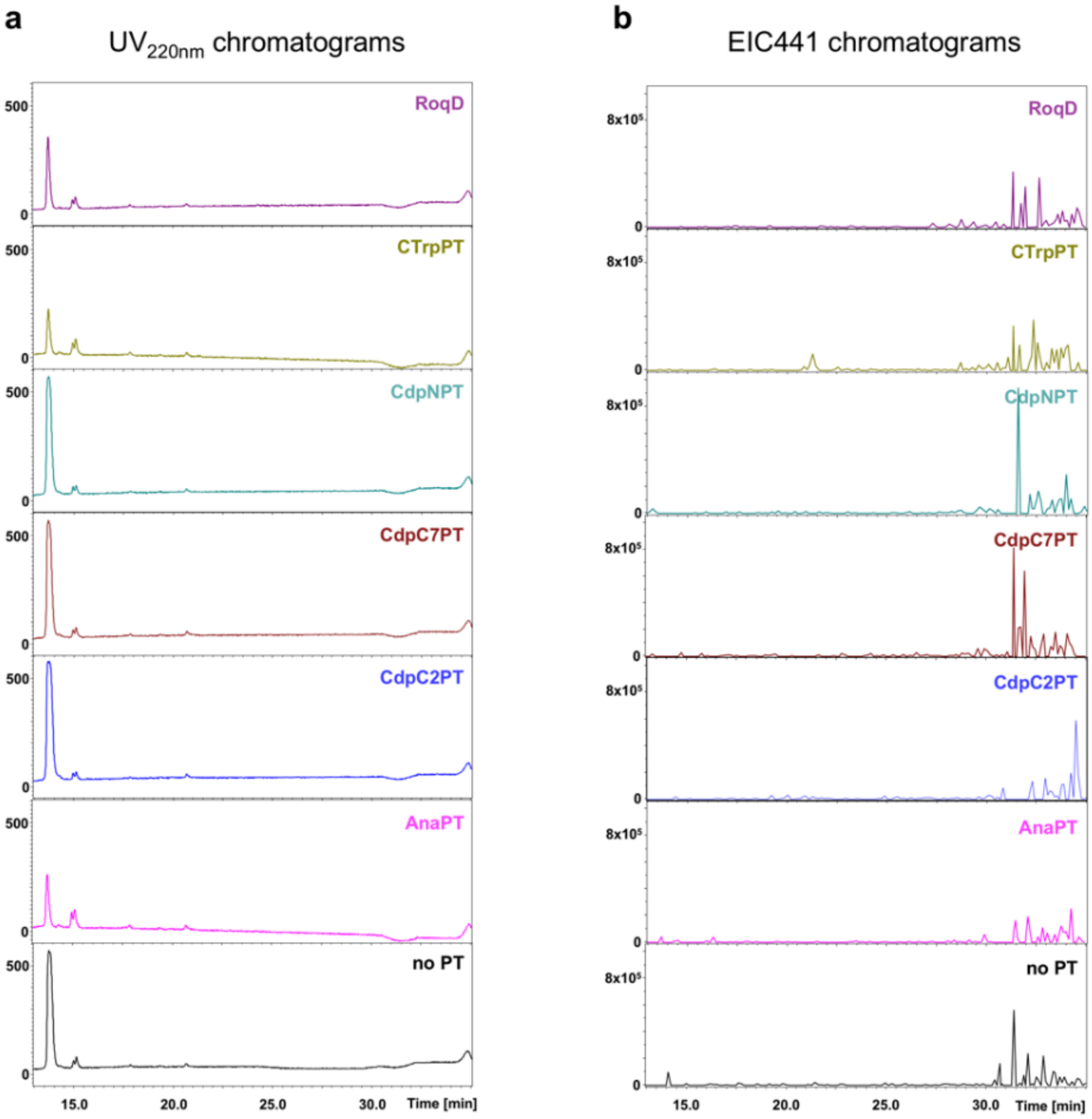


**Supplementary Figure S10.** LC-MSMS analysis of metabolite production by recombinant *E. coli* expressing CDPS14 and PTs. SPE-treated bacterial supernatants of cultures of BL21AI expressing CDPS 14 and AnaPT, CdpC2PT, CdpC7PT, CdpNPT, CTprPT, RoqD or no PT were analysed. UV chromatograms recorded at 220 nm (a) and EIC chromatograms for m/z 441 (b) are shown between 13 and 35 min. The injected volume of SPE eluates corresponded to 50 µl of culture supernatant.

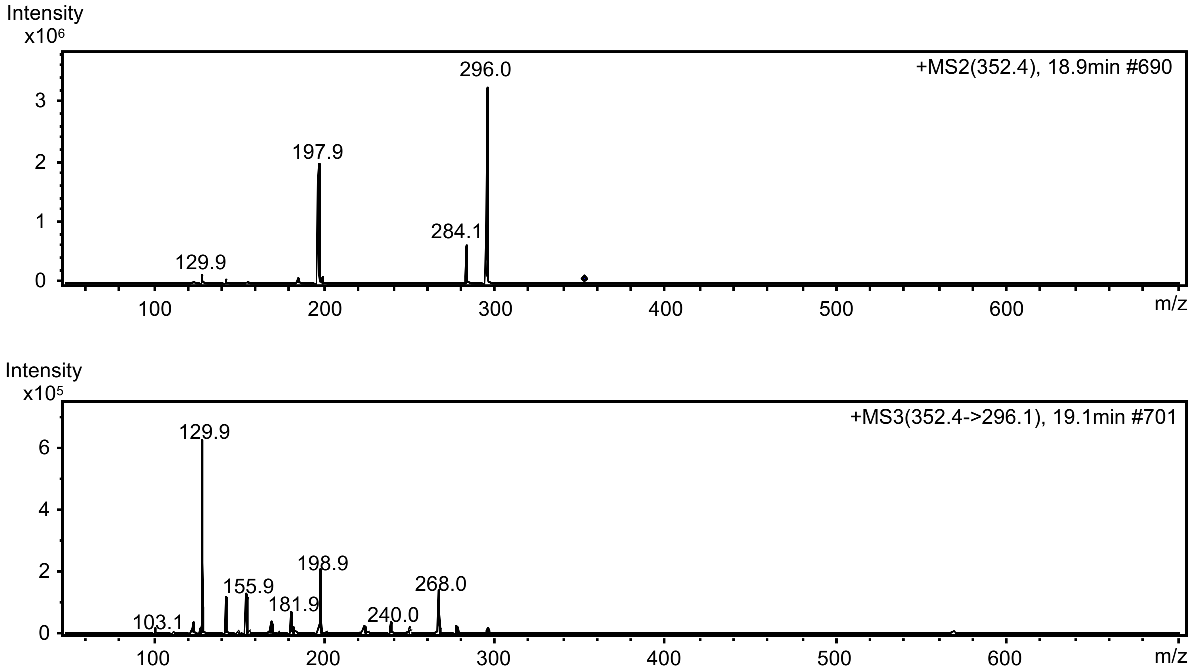


**Supplementary Figure S11.** MS2 and MS3 spectra of the compound produced by recombinant *E. coli* expressing CDPS74 and FtmPT1 with DMAPP pathway engineering with a retention time of 19.0 min in Fig. 3a.

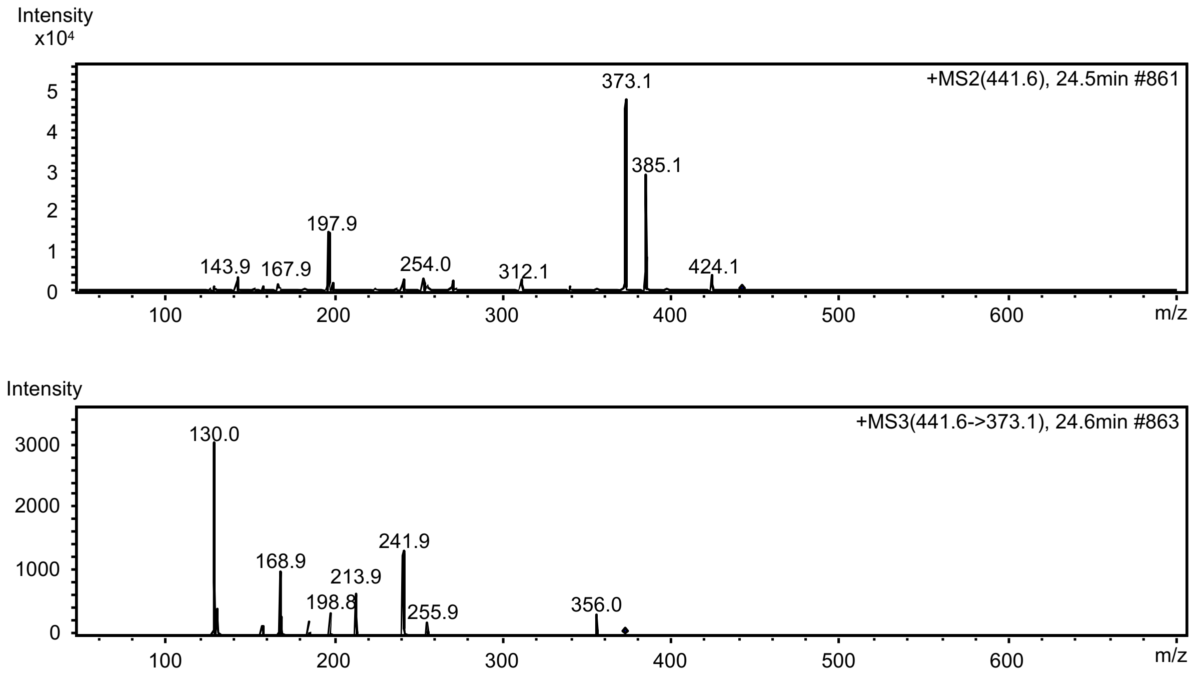


**Supplementary Figure S12.** MS2 and MS3 spectra of the compound produced by recombinant *E. coli* expressing CDPS14 and AnaPT with DMAPP pathway engineering with a retention time of 24.6 min in Fig. 3c.

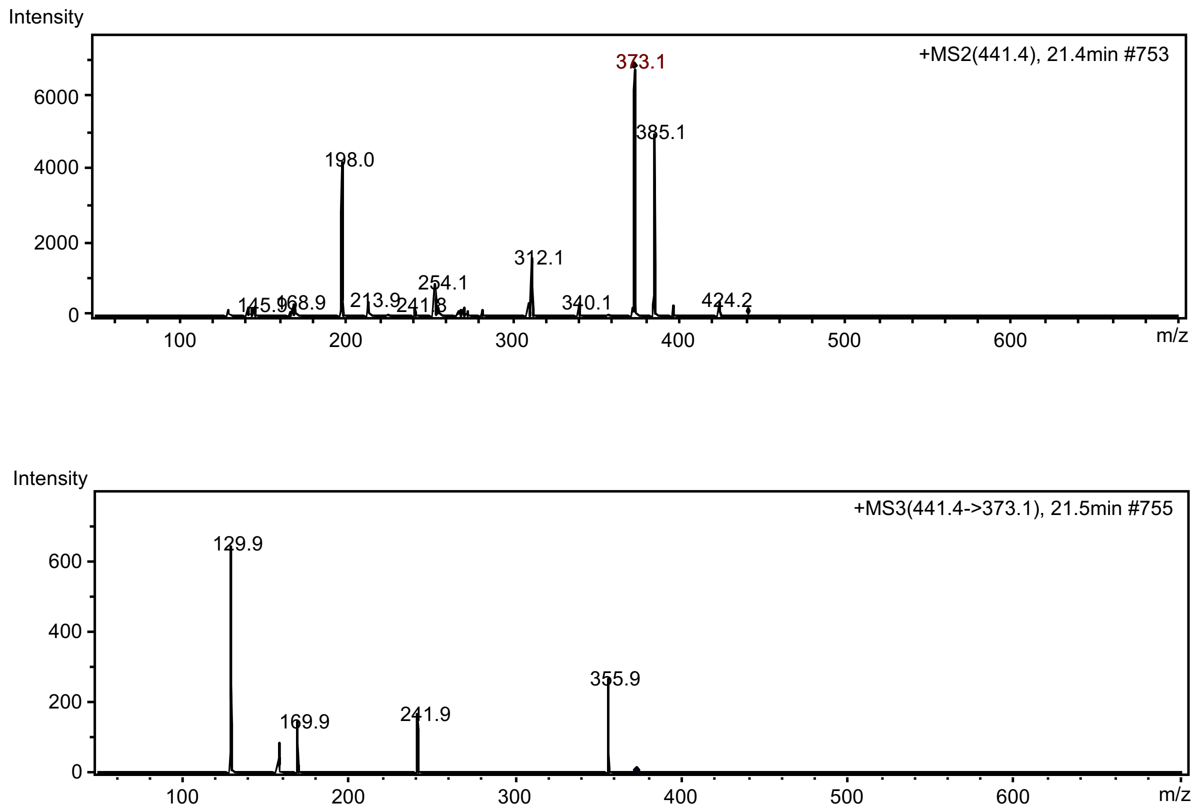


**Supplementary Figure S13.** MS2 and MS3 spectra of the compound produced by recombinant *E. coli* expressing CDPS14 and CTrpPT with DMAPP pathway engineering with a retention time of 21.4 min in Fig. 3c.

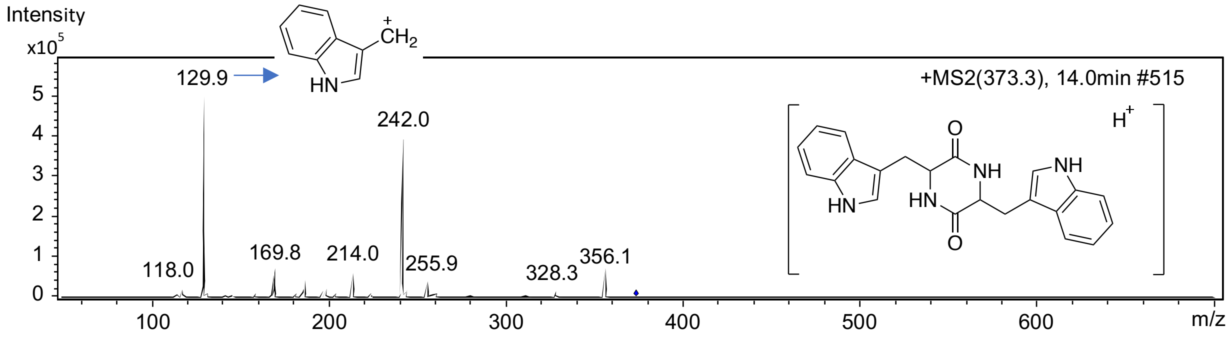


**Supplementary Figure S14.** MS2 spectrum of cWW produced by recombinant *E. coli* expressing CDPS14 with DMAPP pathway engineering. The chemical structure of the $MH^+$ ion at m/z 130 characteristic of the indole ring is shown.

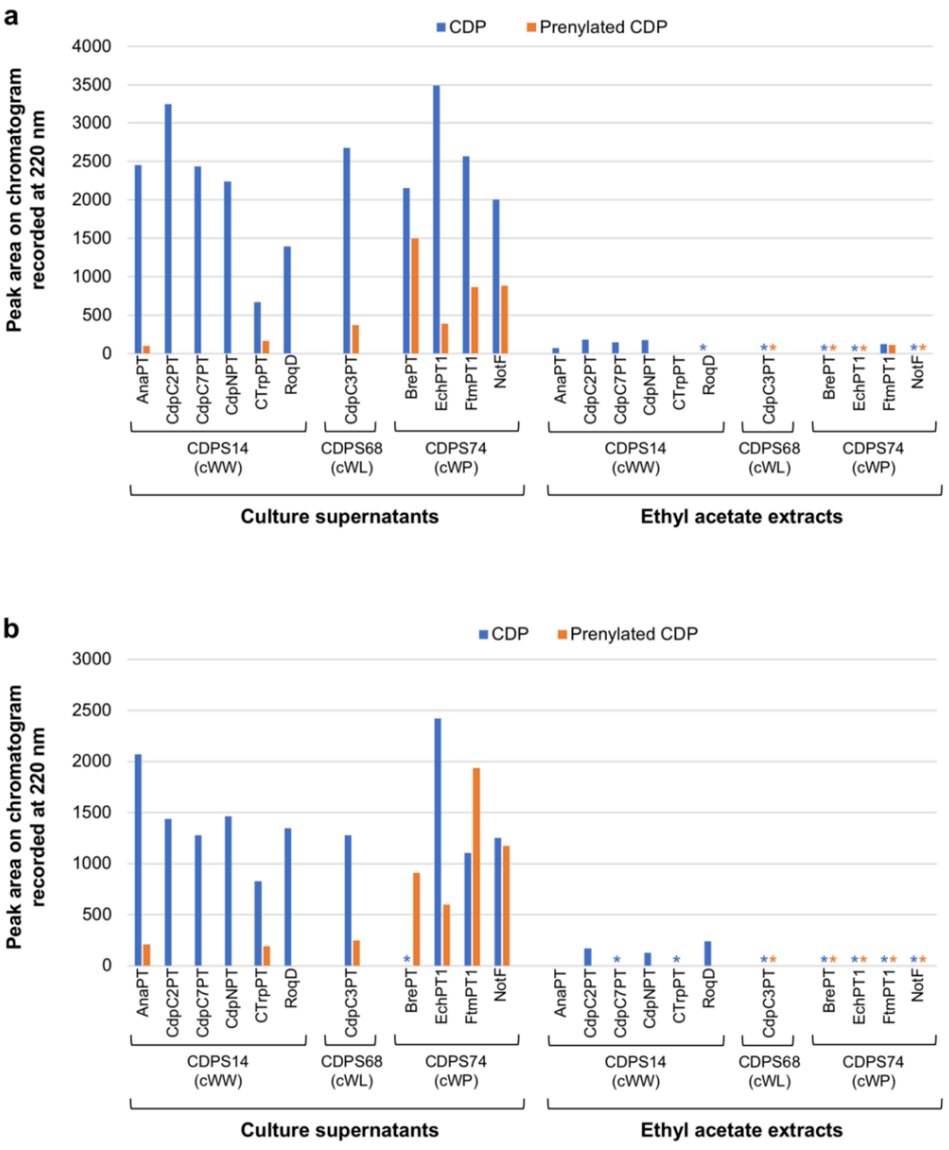


**Supplementary Figure S15.** Recovery of CDPs and prenylated CDPs from different bacterial culture fractions. a) BL21AI bacteria carrying pJBEI3085 and the 11 CDPS/PT combinations tested in our study where grown and induced for prenylated CDPs production. Culture supernatants were treated by SPE as described. Bacteria were lysed by sonication and the lysates extracted with ethyl acetate. Samples corresponding to culture supernatants and bacterial lysate extracts were analysed by LC-MSMS. The injected volumes corresponded to 50 µl of the original cultures. Peak areas on chromatograms recorded at 220 nm are shown for CDPs (blue) and prenylated CDPs (orange) found in the culture supernatants and in ethyl acetate extracts. When several prenylated CDPs were detected, their peak areas were summed. Blue stars indicate CDPs and orange stars prenylated CDPs that were detected only in EIC traces. b) The experiment was repeated using a chemical lysis protocol to prepare the bacterial lysates. The legend is as in a).

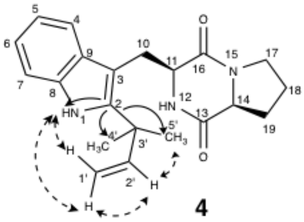

**4**

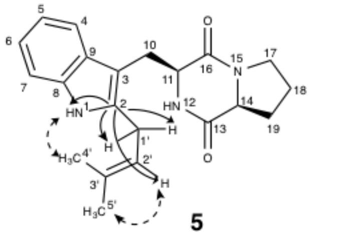

**5**

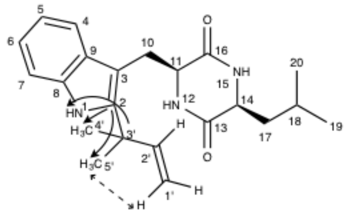

**6**

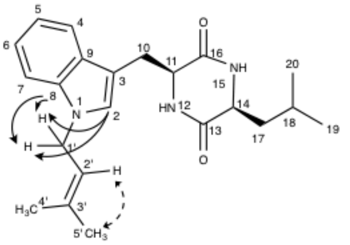

**7**

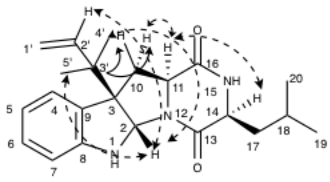

**8**

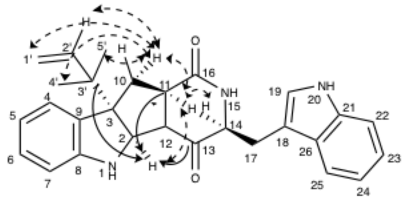

**9**

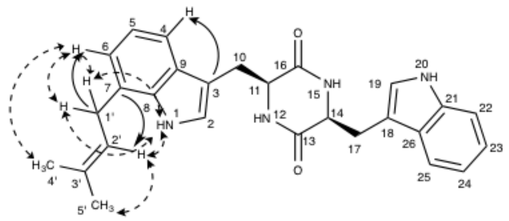

**10**

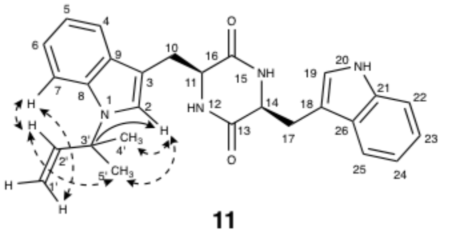

**11**

**Supplementary Figure S16**. Key HMBC and ROE connectivities used to elucidate the structure of compounds **4-11**. $^{1}$H-$^{13}$C HMBC correlations are indicated by plain arrows (from $^{13}$C to $^{1}$H) and $^{1}$H-$^{1}$H ROEs are indicated by dashed arrows.

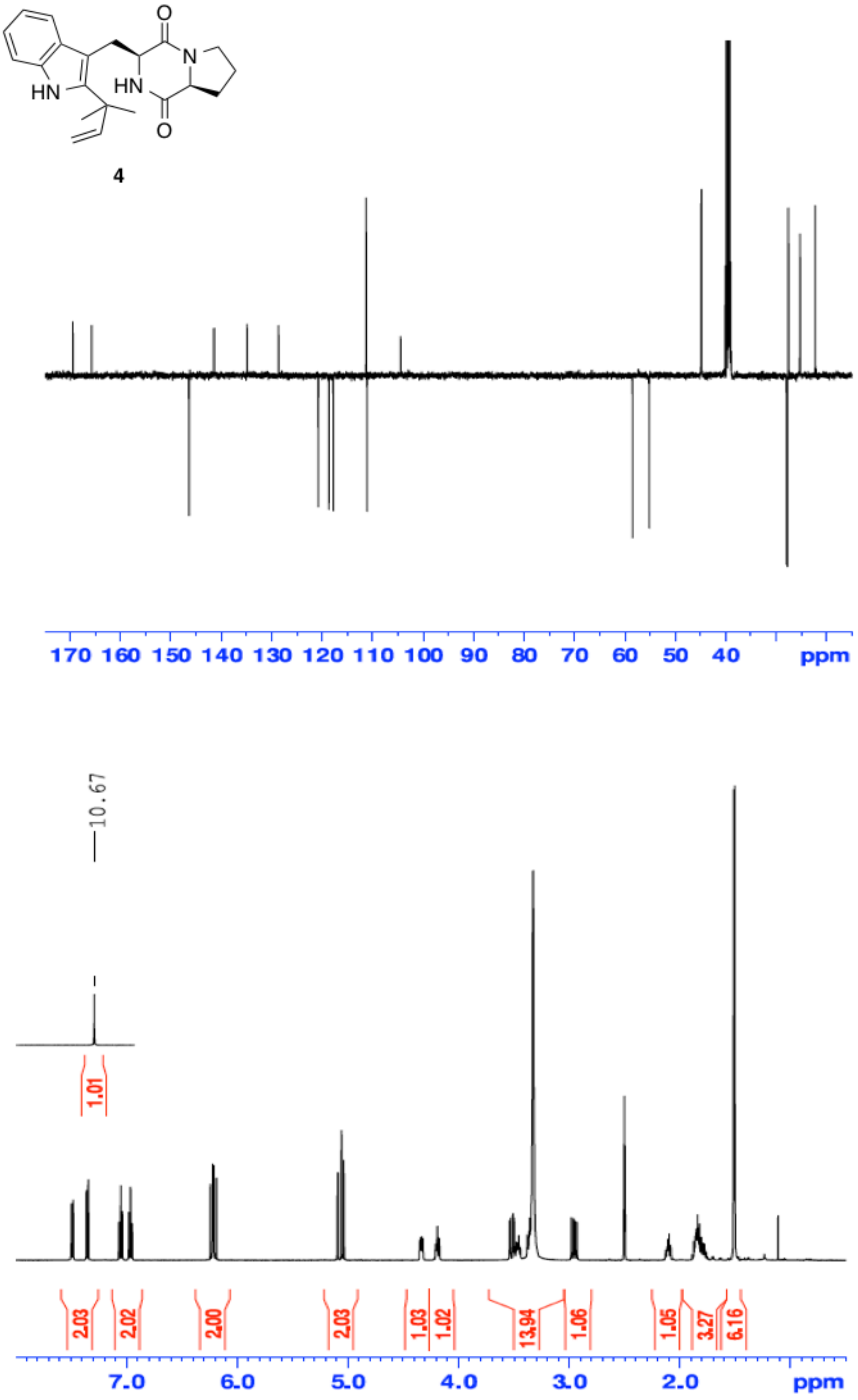


**Supplementary Figure S17**. 1D $^{13}C$ DEPTQ spectrum (upper panel) and 1D $^{1}H$ spectrum (lower panel) of compound **4** in DMSO-$d_6$.

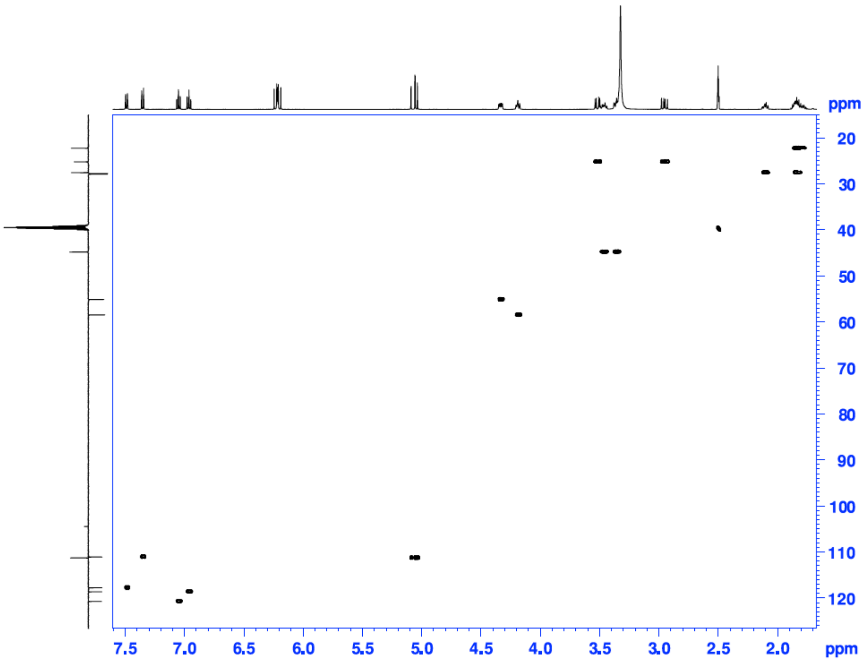


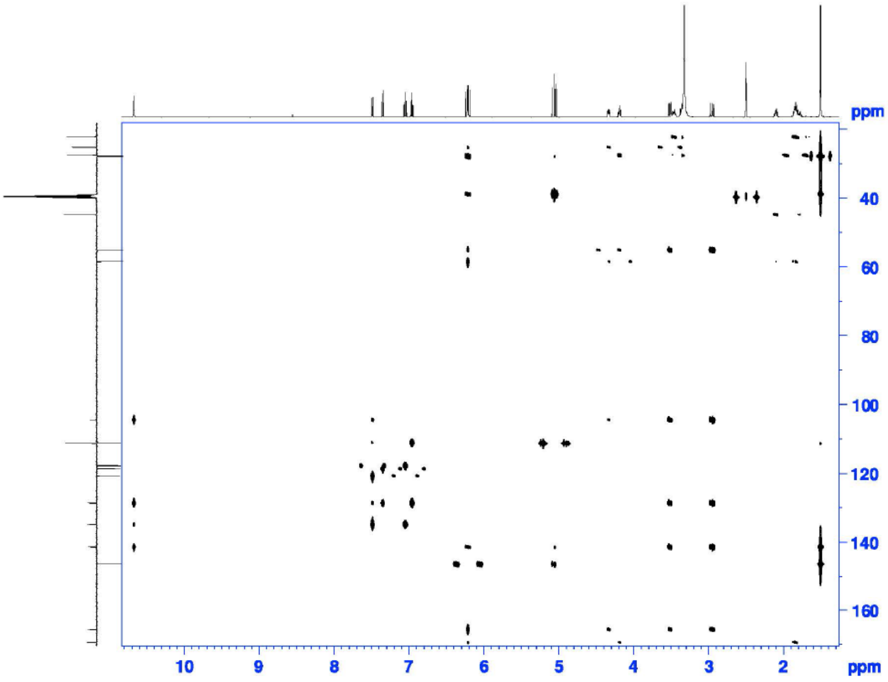


**Supplementary Figure S18**. 2D $^{13}C$-$^{1}H$ HSQC spectrum (upper panel) and 2D $^{13}C$-$^{1}H$ HMBC spectrum (lower panel) of compound **4** in DMSO-*d6*.

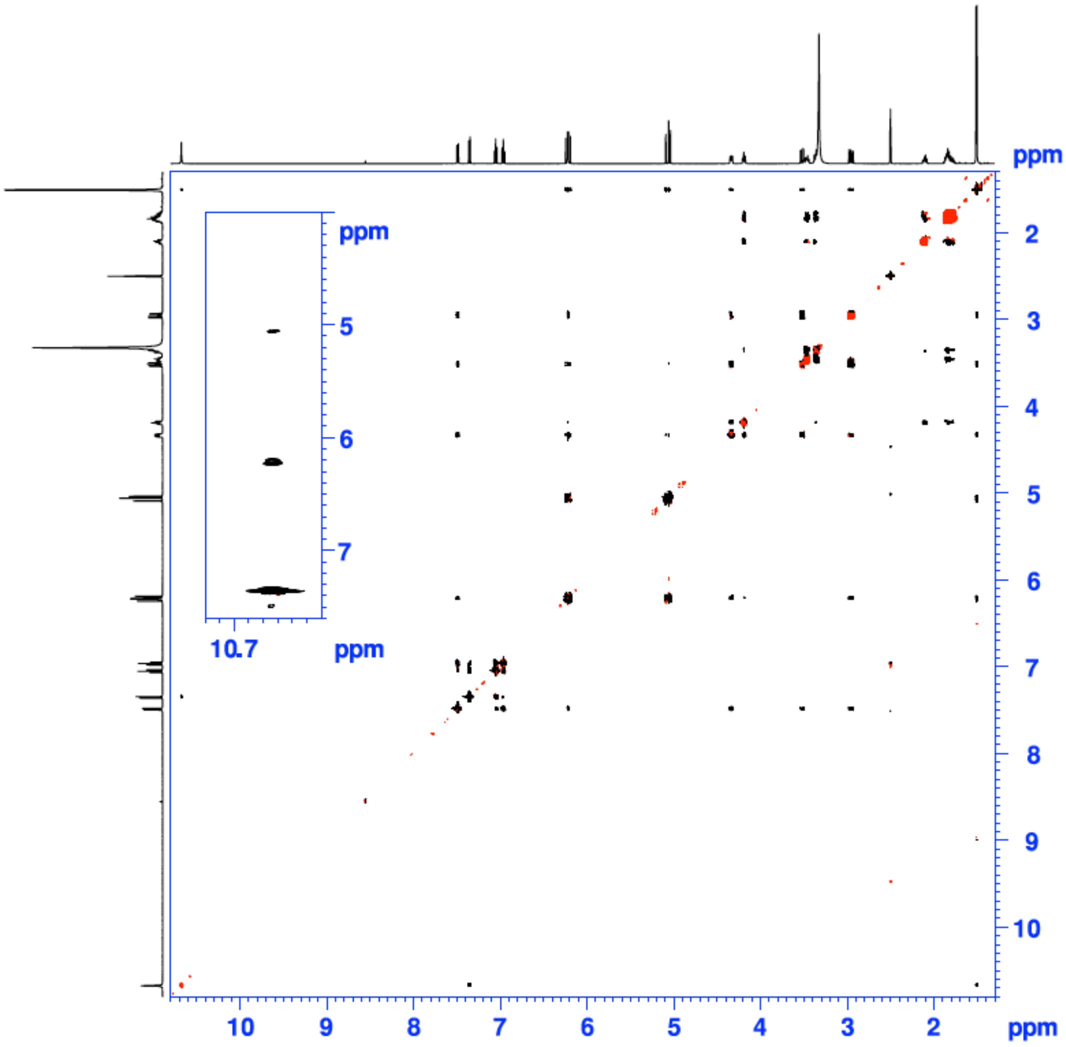


**Supplementary Figure S19**. 2D $^{1}H$-$^{1}H$ ROESY spectrum of compound **4** in DMSO-*d6*.

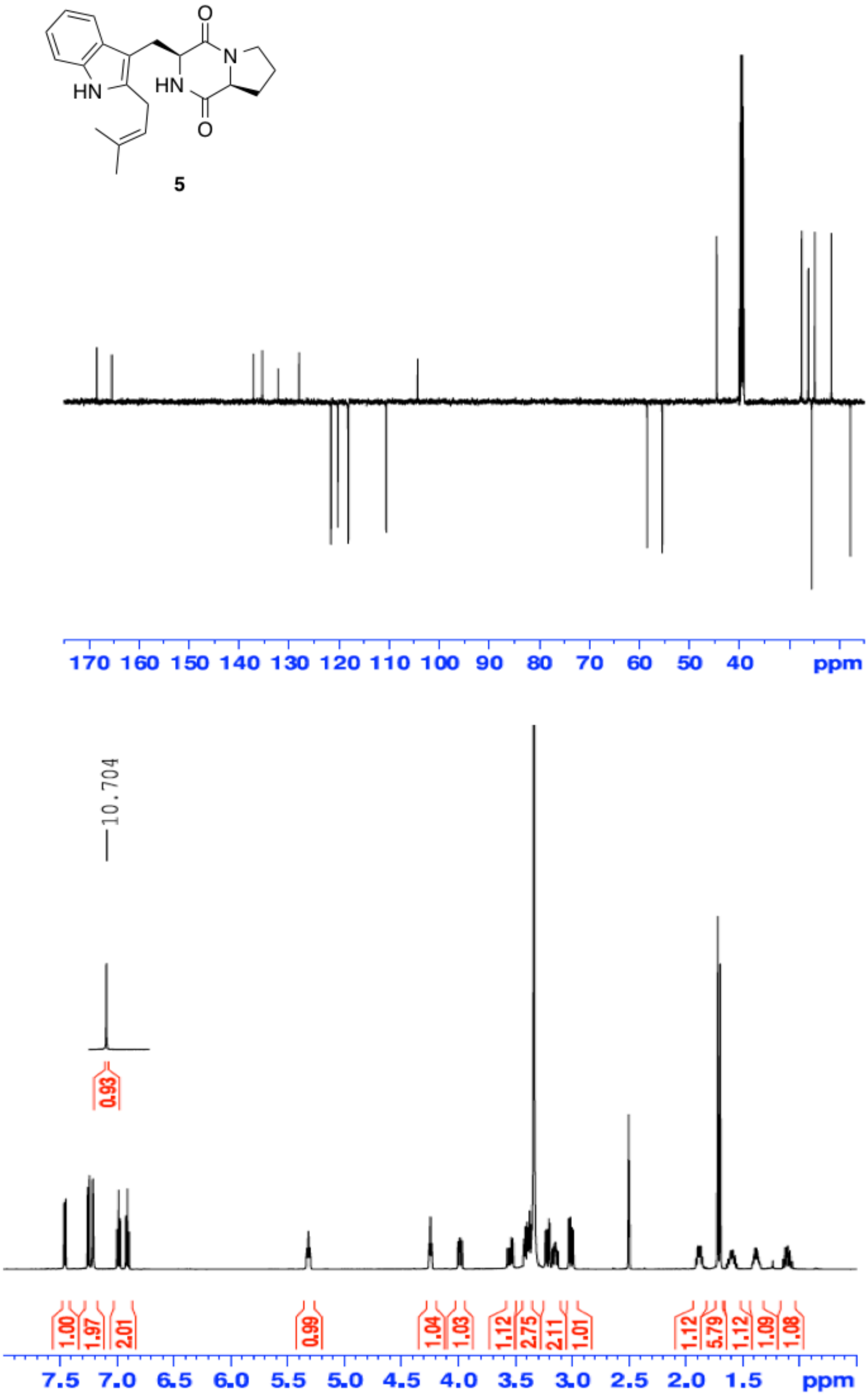


**Supplementary Figure S20**. 1D $^{13}C$ DEPTQ spectrum (upper panel) and 1D $^{1}H$ spectrum (lower panel) of compound **5** in DMSO-$d_6$.

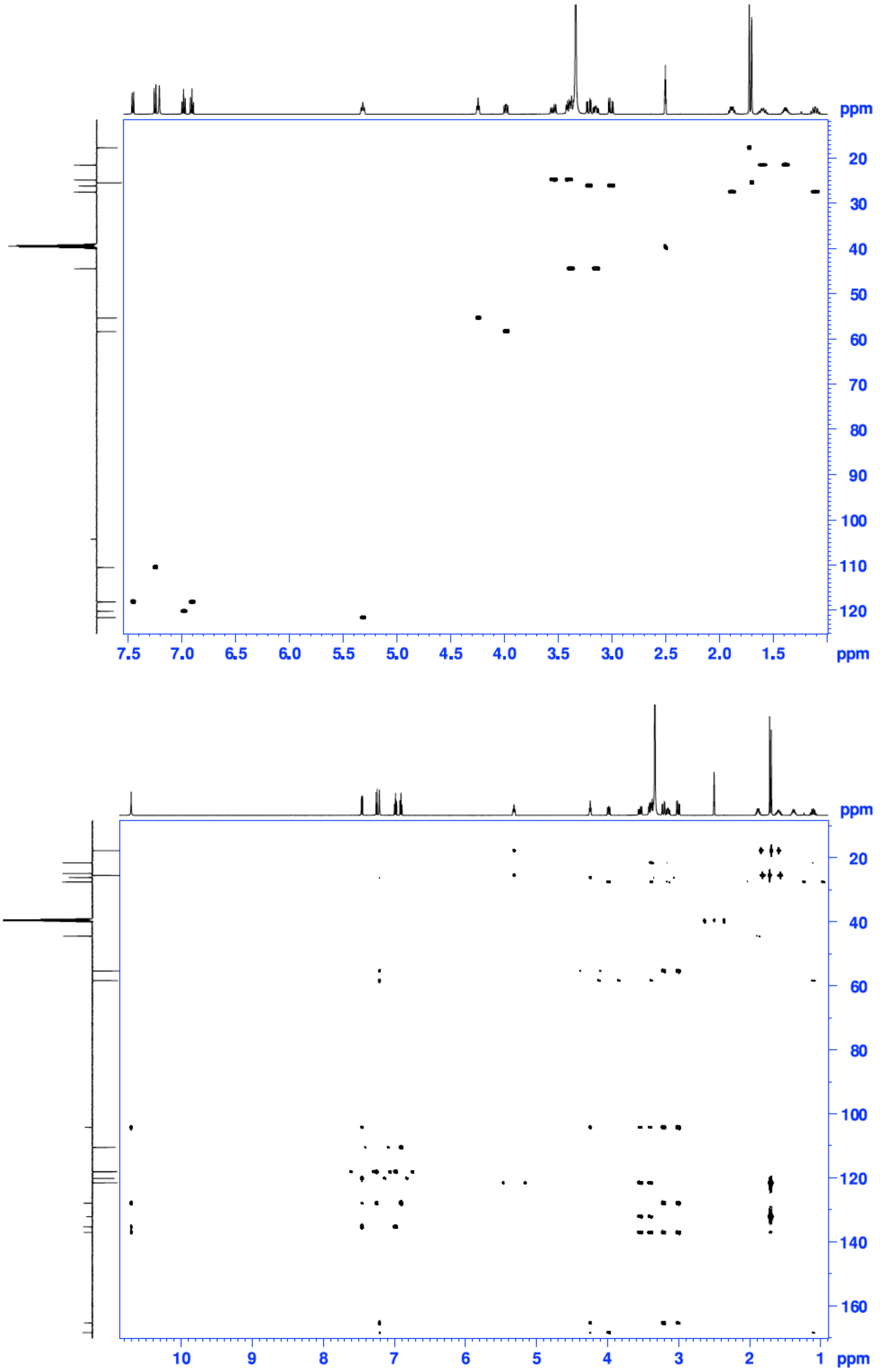


**Supplementary Figure S21**. 2D $^{13}C$-$^{1}H$ HSQC spectrum (upper panel) and 2D $^{13}C$-$^{1}H$ HMBC spectrum (lower panel) of compound **5** in DMSO-*d6*.

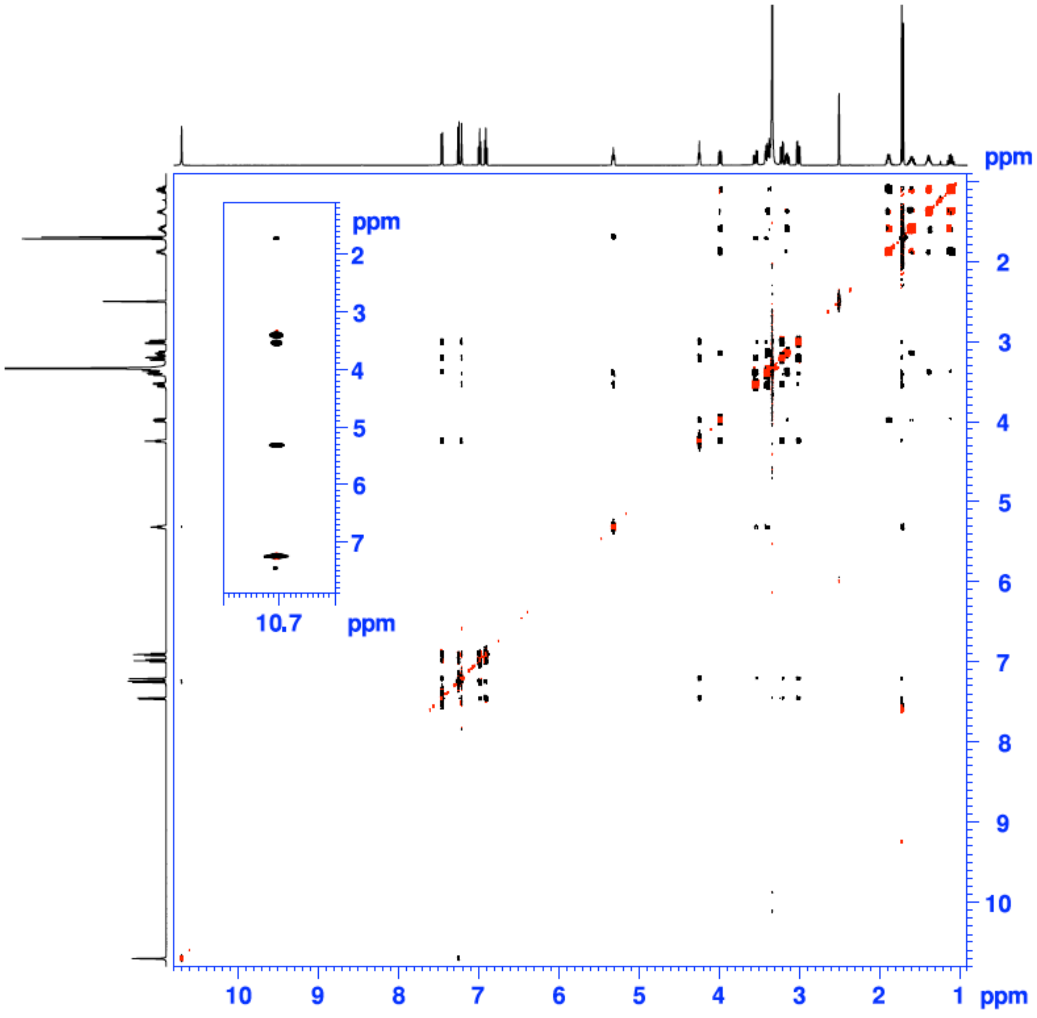


**Supplementary Figure S22**. 2D $^{1}H$-$^{1}H$ ROESY spectrum of compound **5** in DMSO-*d6*.

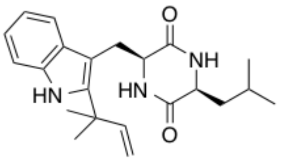


**6**

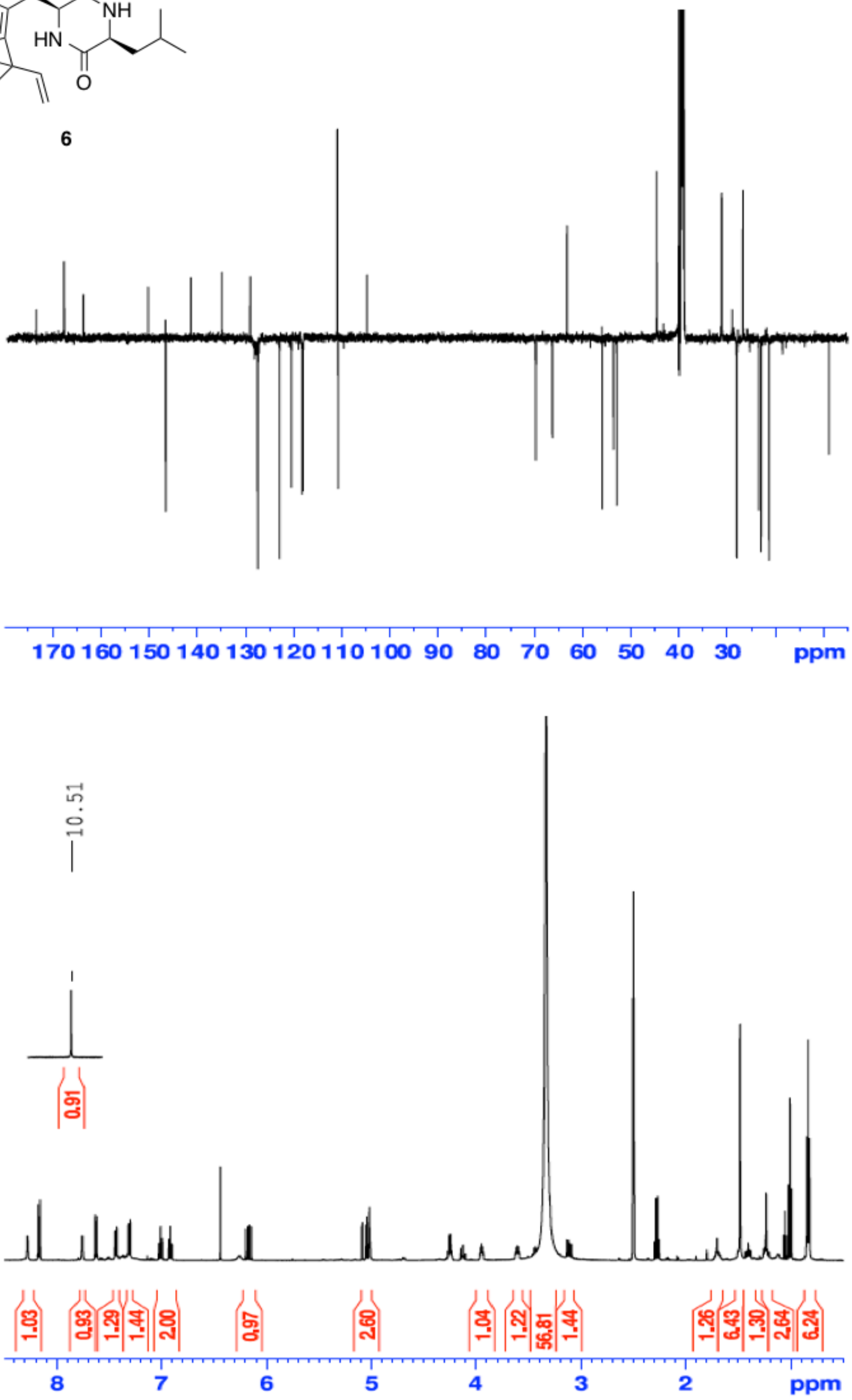


**Supplementary Figure S23**. 1D $^{13}C$ DEPTQ spectrum (upper panel) and 1D $^{1}H$ spectrum (lower panel) of compound **6** in DMSO-$d_6$.

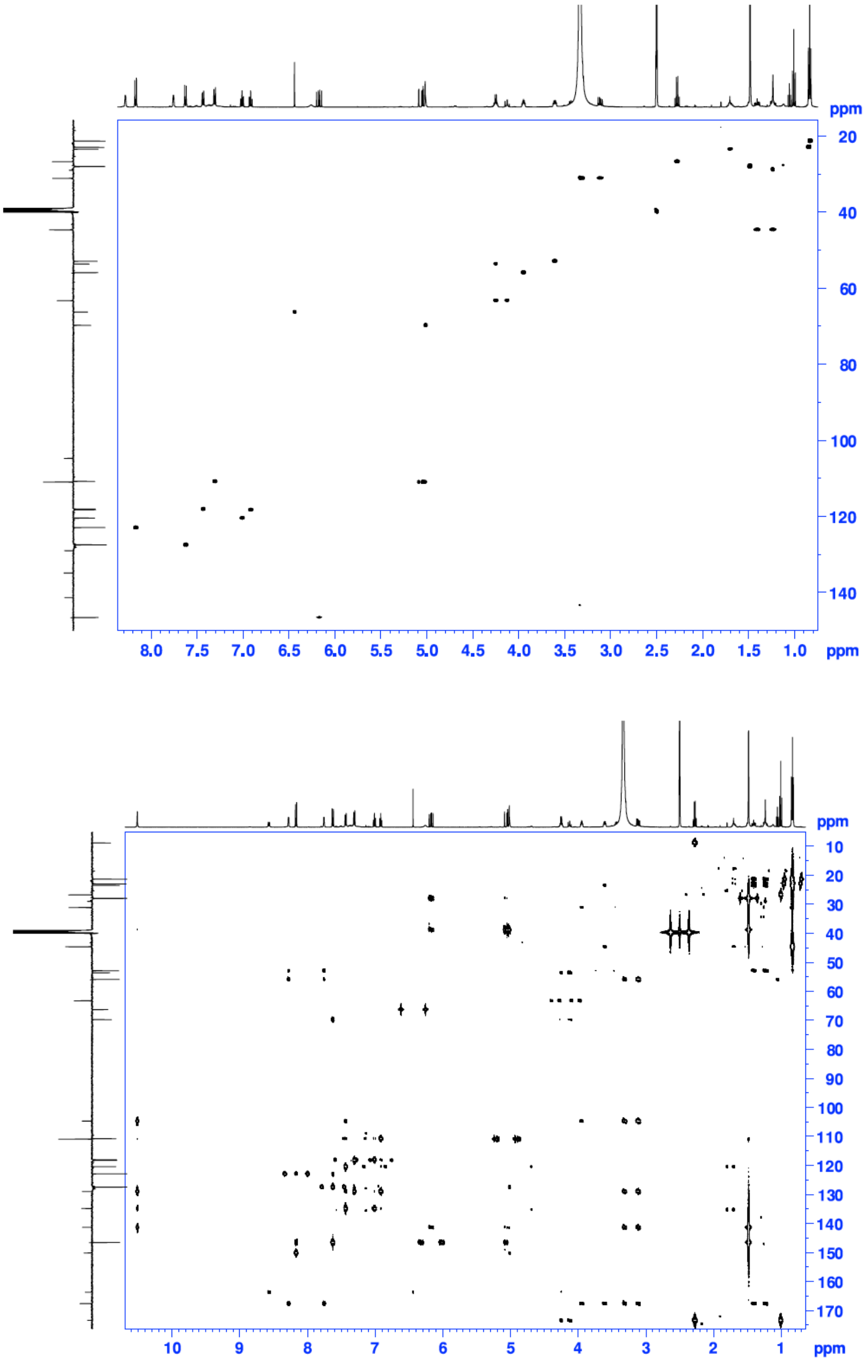


**Supplementary Figure S24**. 2D $^{13}$C-$^{1}$H HSQC spectrum (upper panel) and 2D $^{13}$C-$^{1}$H HMBC spectrum (lower panel) of compound **6** in DMSO-*d6*.

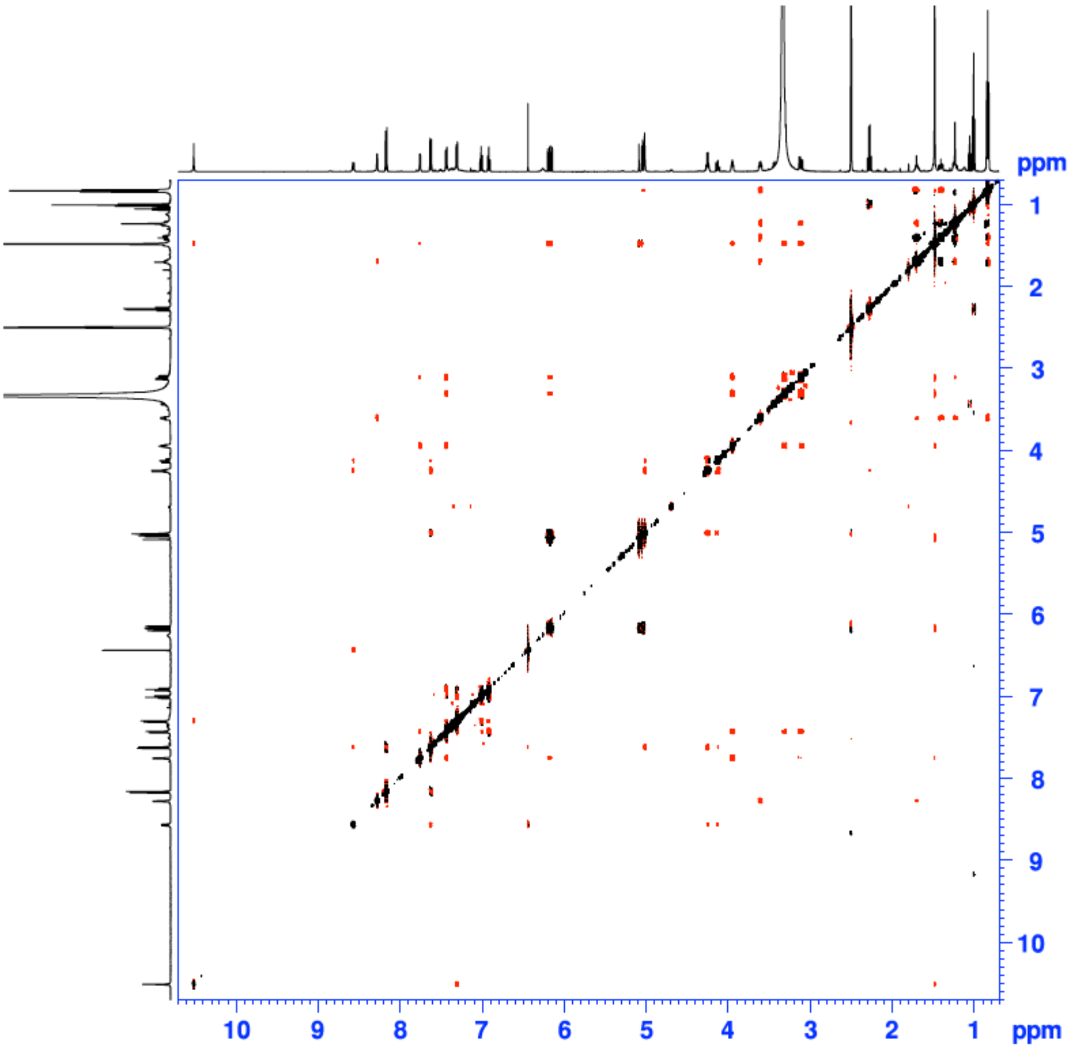


**Supplementary Figure S25**. 2D $^{1}H$-$^{1}H$ ROESY spectrum of compound **6** in DMSO-*d6*.

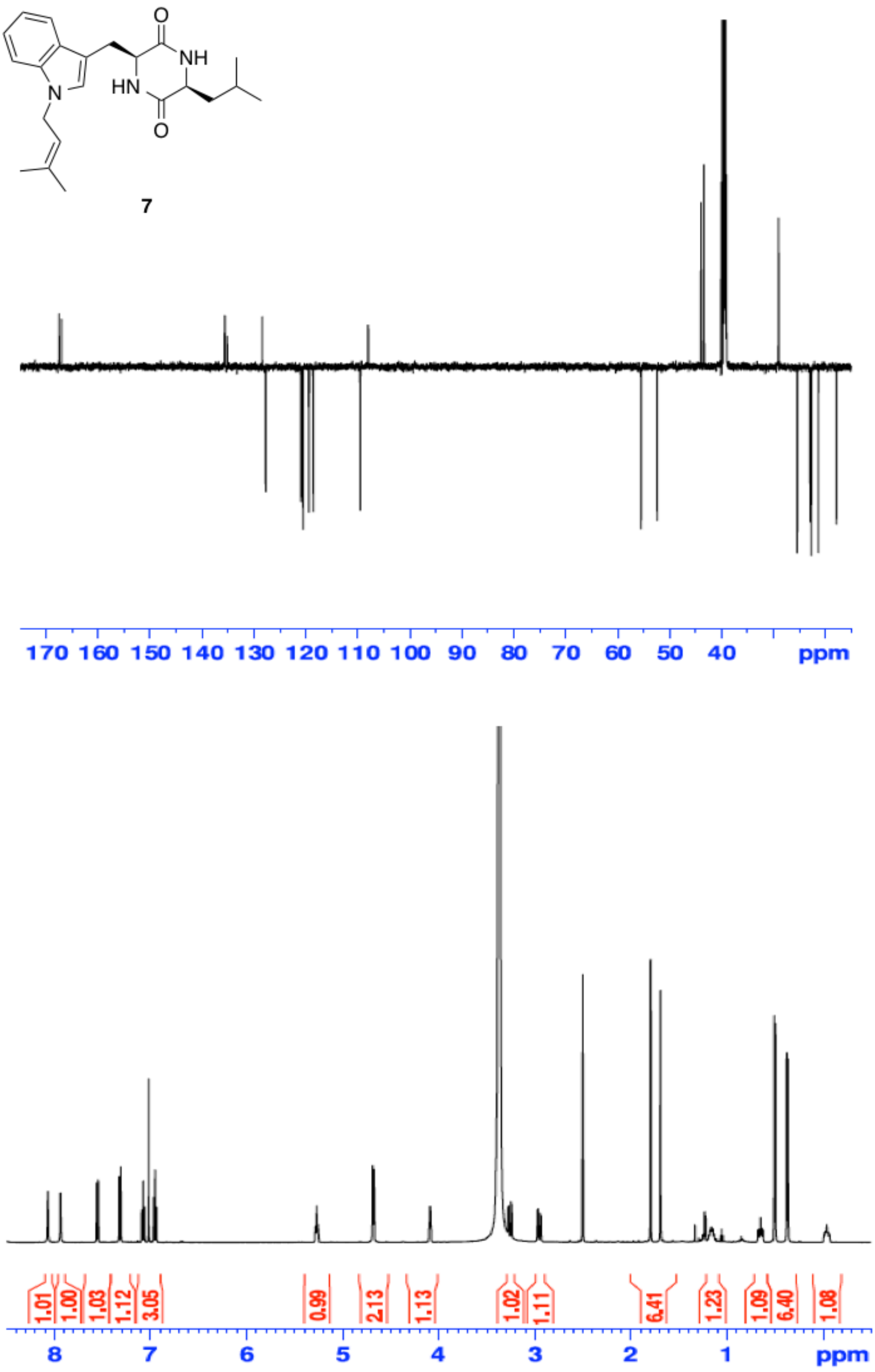


**Supplementary Figure S26**. 1D $^{13}C$ DEPTQ spectrum (upper panel) and 1D $^{1}H$ spectrum (lower panel) of compound **7** in DMSO-$d_6$.

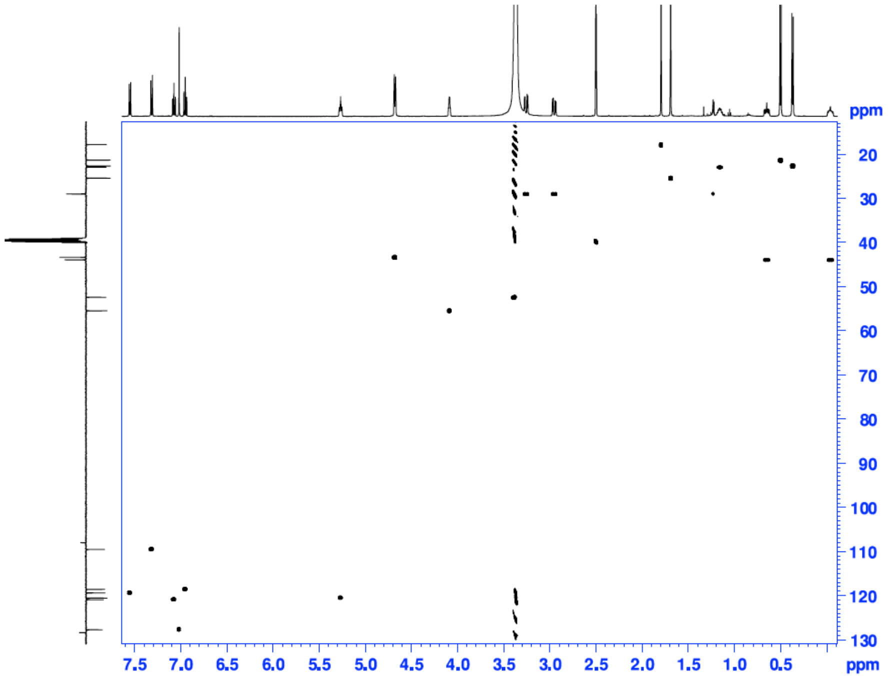


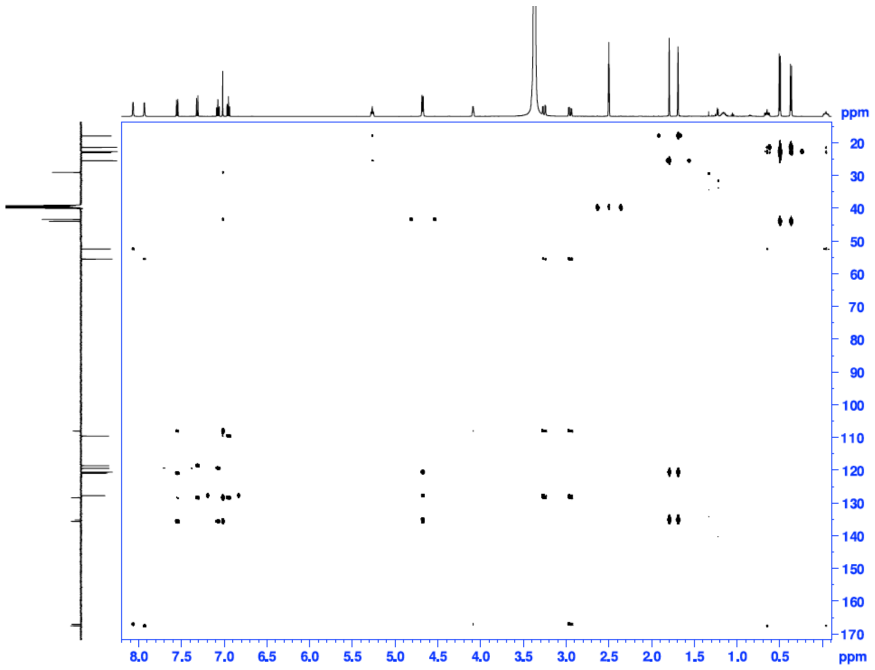


**Supplementary Figure S27**. 2D $^{13}C$-$^{1}H$ HSQC spectrum (upper panel) and 2D $^{13}C$-$^{1}H$ HMBC spectrum (lower panel) of compound **7** in DMSO-*d6*.

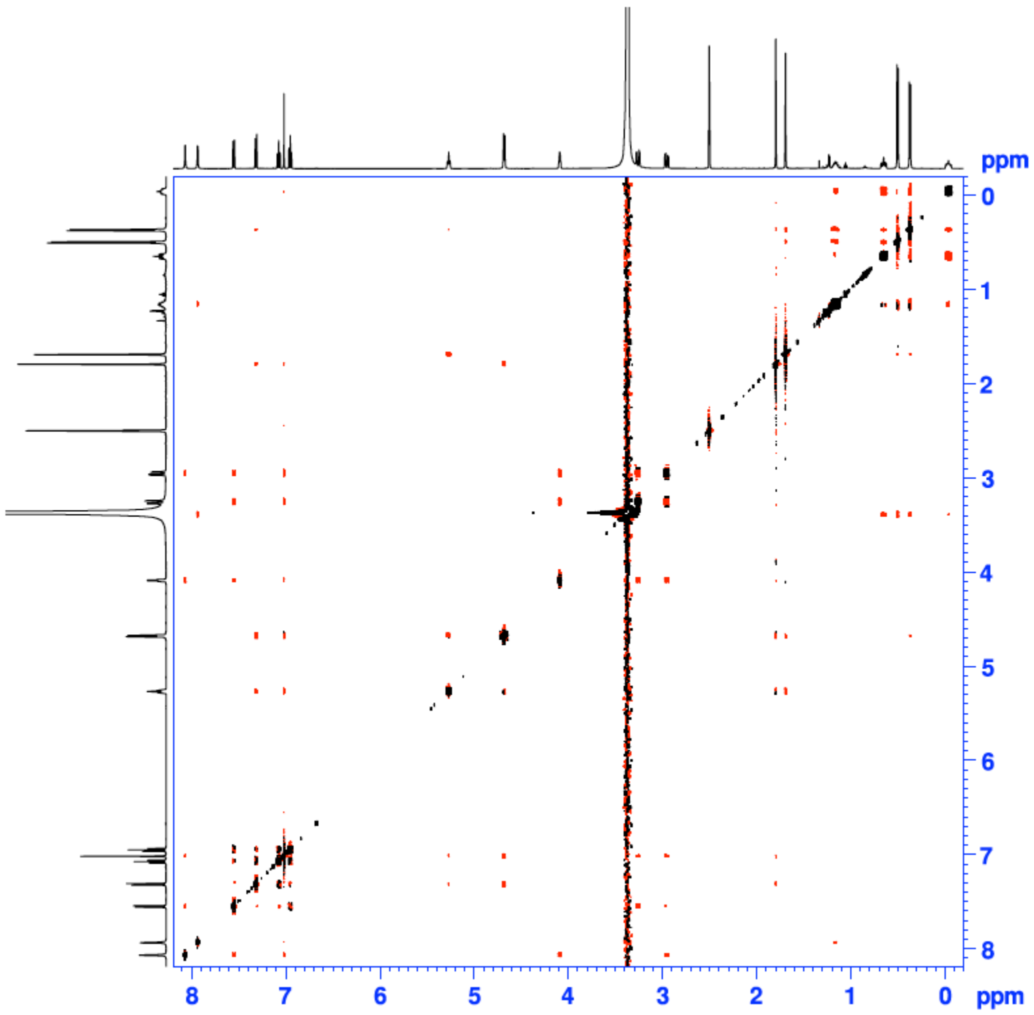


**Supplementary Figure S28**. 2D $^{1}H$-$^{1}H$ ROESY spectrum of compound **7** in DMSO-*d6*.

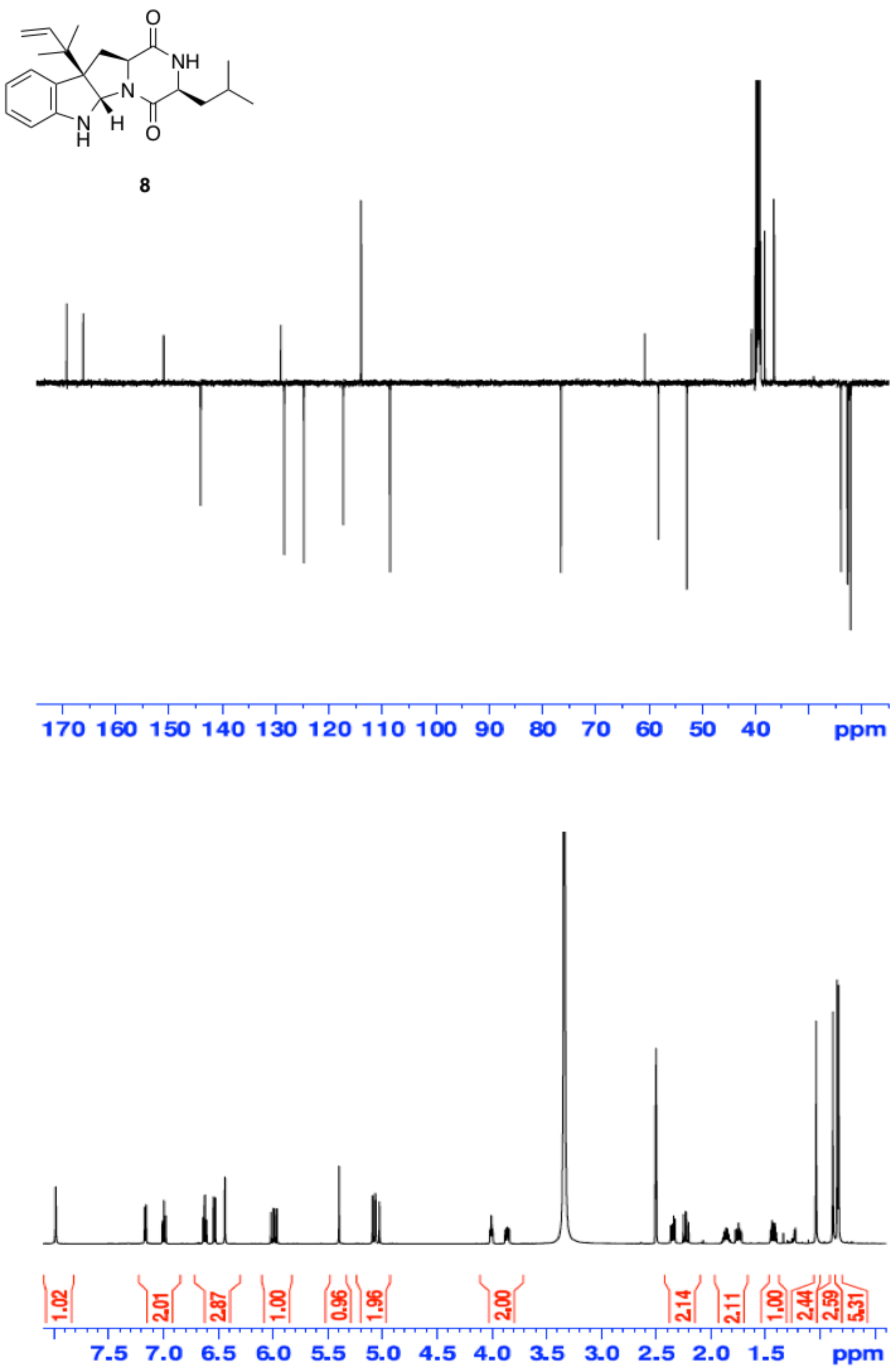


**Supplementary Figure S29**. 1D $^{13}C$ DEPTQ spectrum (upper panel) and 1D $^{1}H$ spectrum (lower panel) of compound **8** in DMSO-$d_6$.

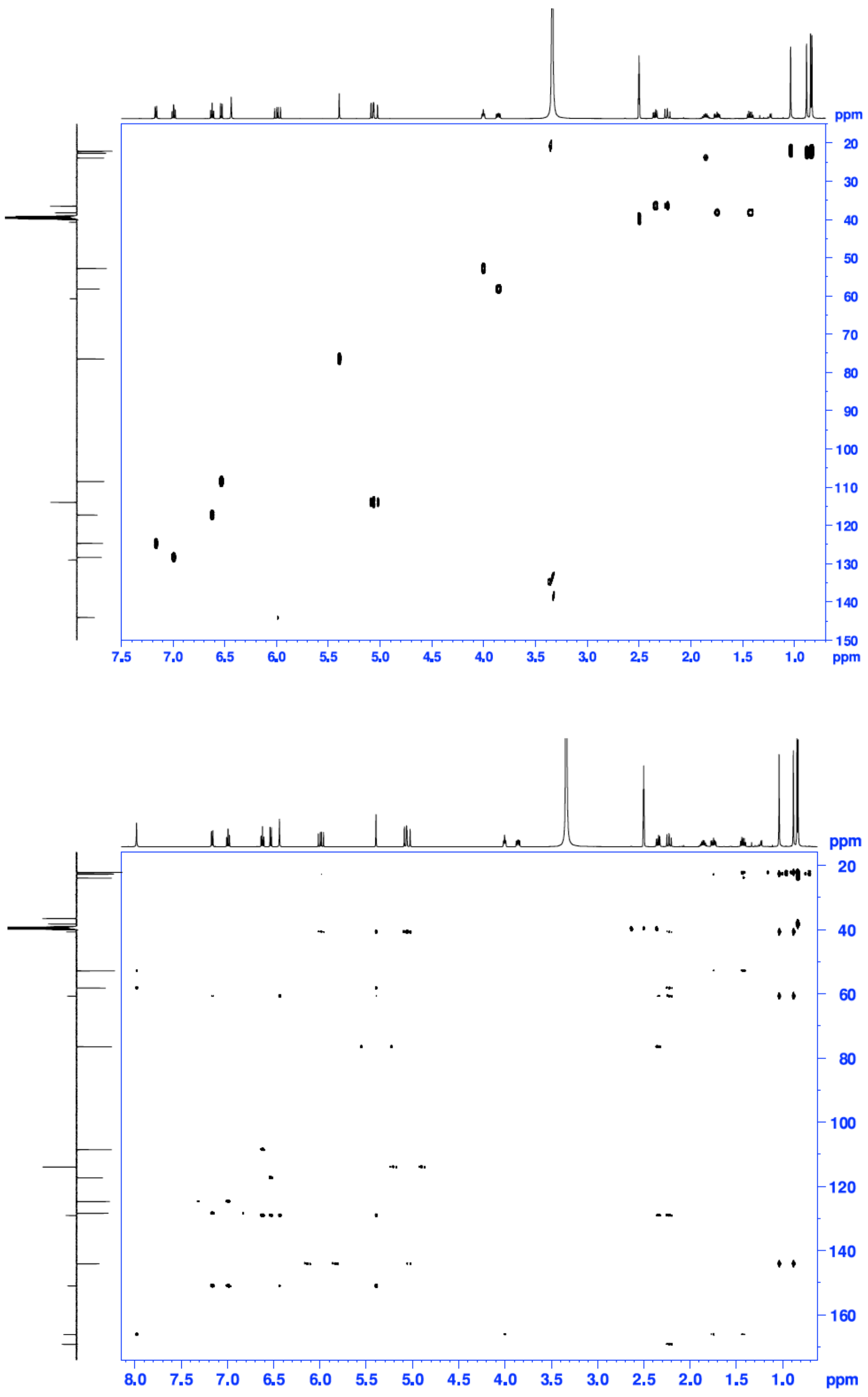


**Supplementary Figure S30**. 2D $^{13}C$-$^{1}H$ HSQC spectrum (upper panel) and 2D $^{13}C$-$^{1}H$ HMBC spectrum (lower panel) of compound **8** in DMSO-*d6*.

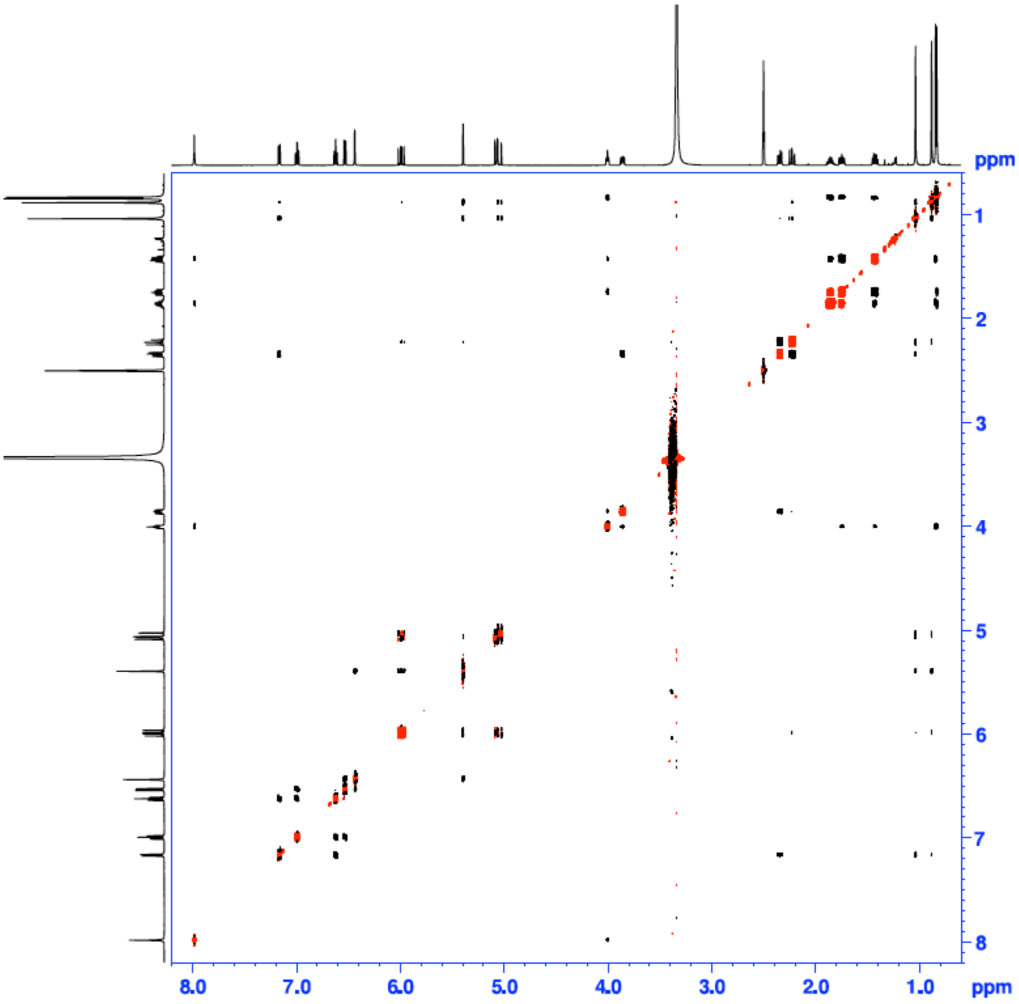


**Supplementary Figure S31**. 2D $^{1}$H-$^{1}$H ROESY spectrum of compound **8** in DMSO-*d6*.

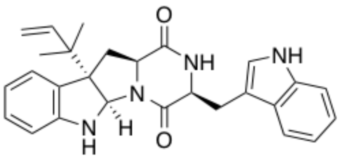


**9**

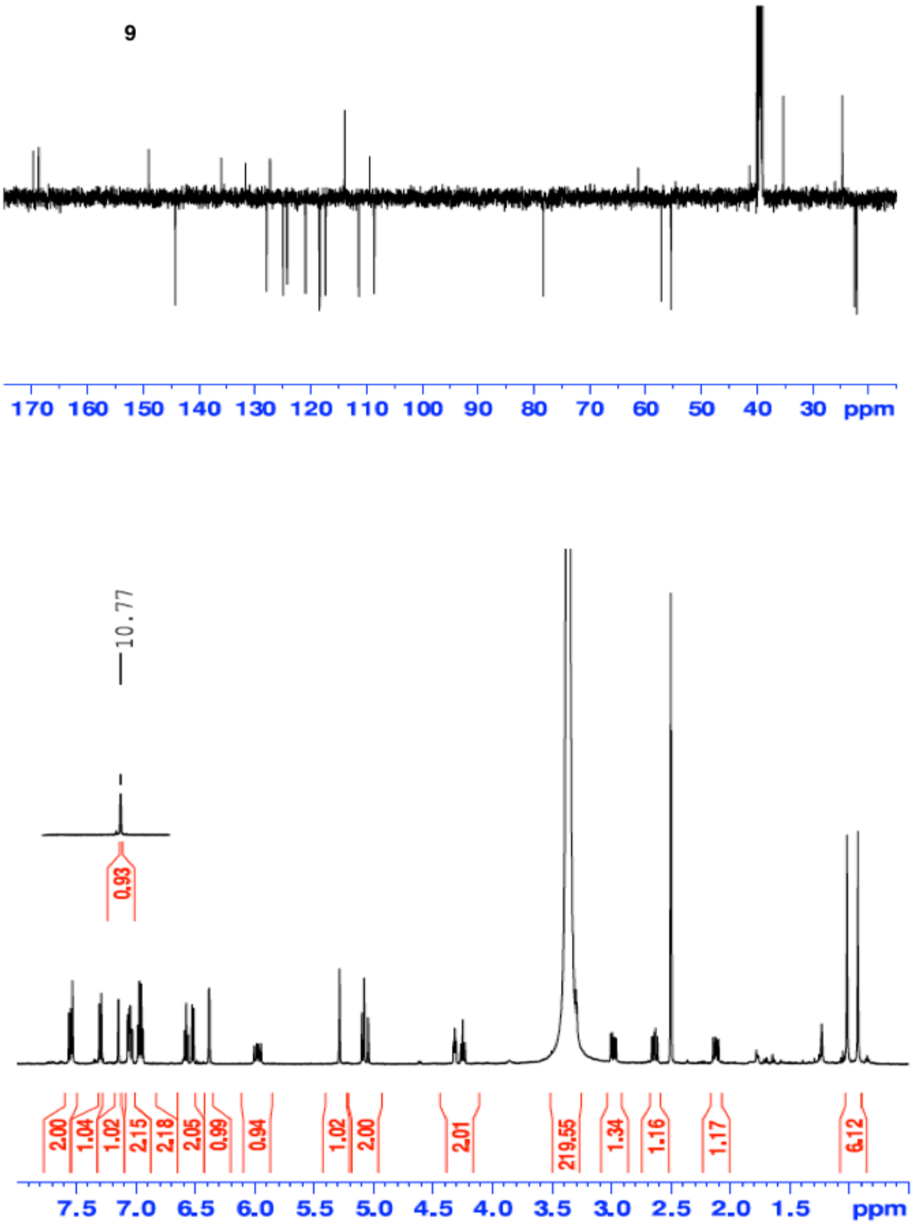


**Supplementary Figure S32**. 1D $^{13}$C DEPTQ spectrum (upper panel) and 1D $^{1}$H spectrum (lower panel) of compound **9** in DMSO-$d_6$.

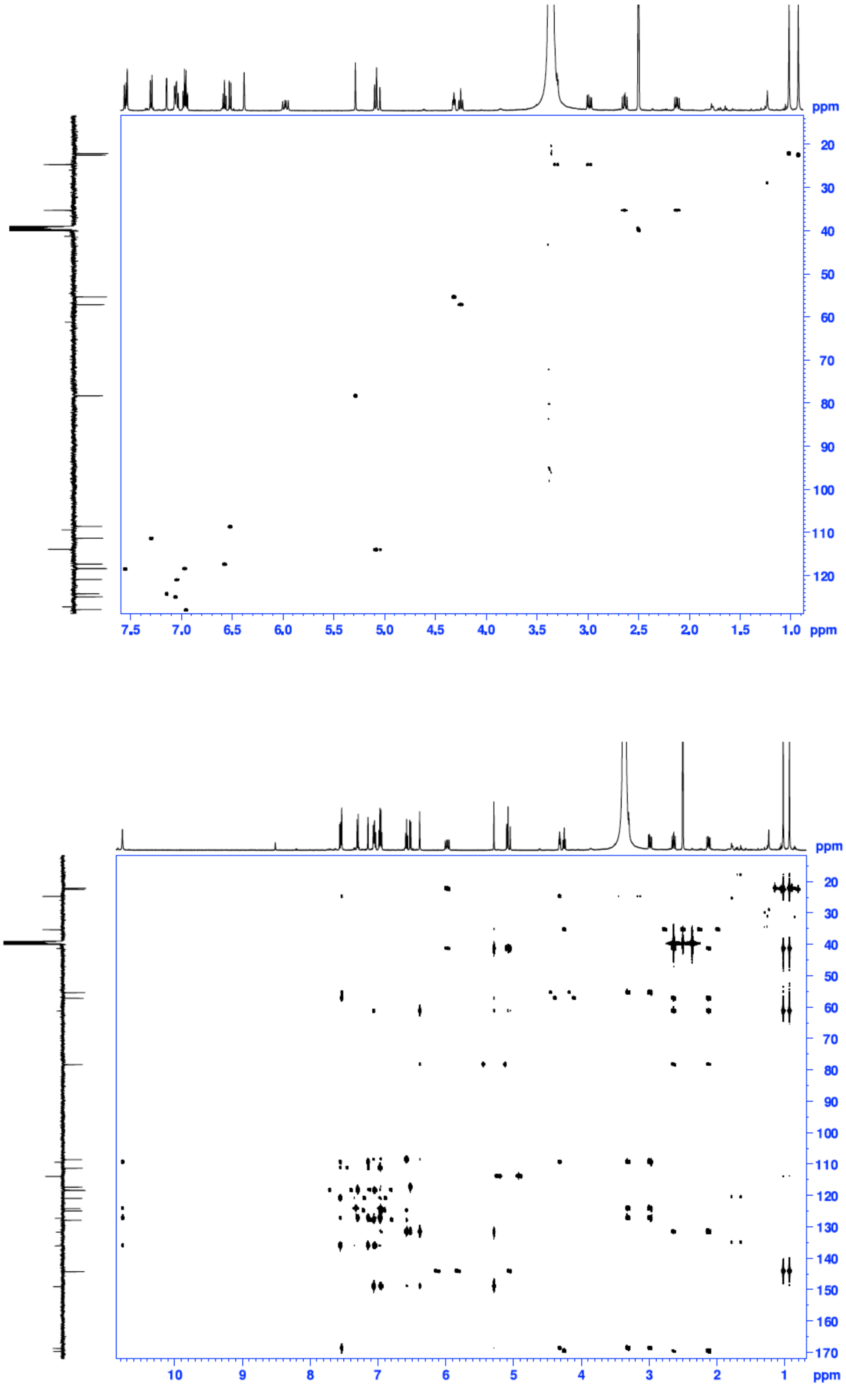


**Supplementary Figure S33**. 2D $^{13}$C-$^{1}$H HSQC spectrum (upper panel) and 2D $^{13}$C-$^{1}$H HMBC spectrum (lower panel) of compound **9** in DMSO-*d6*.

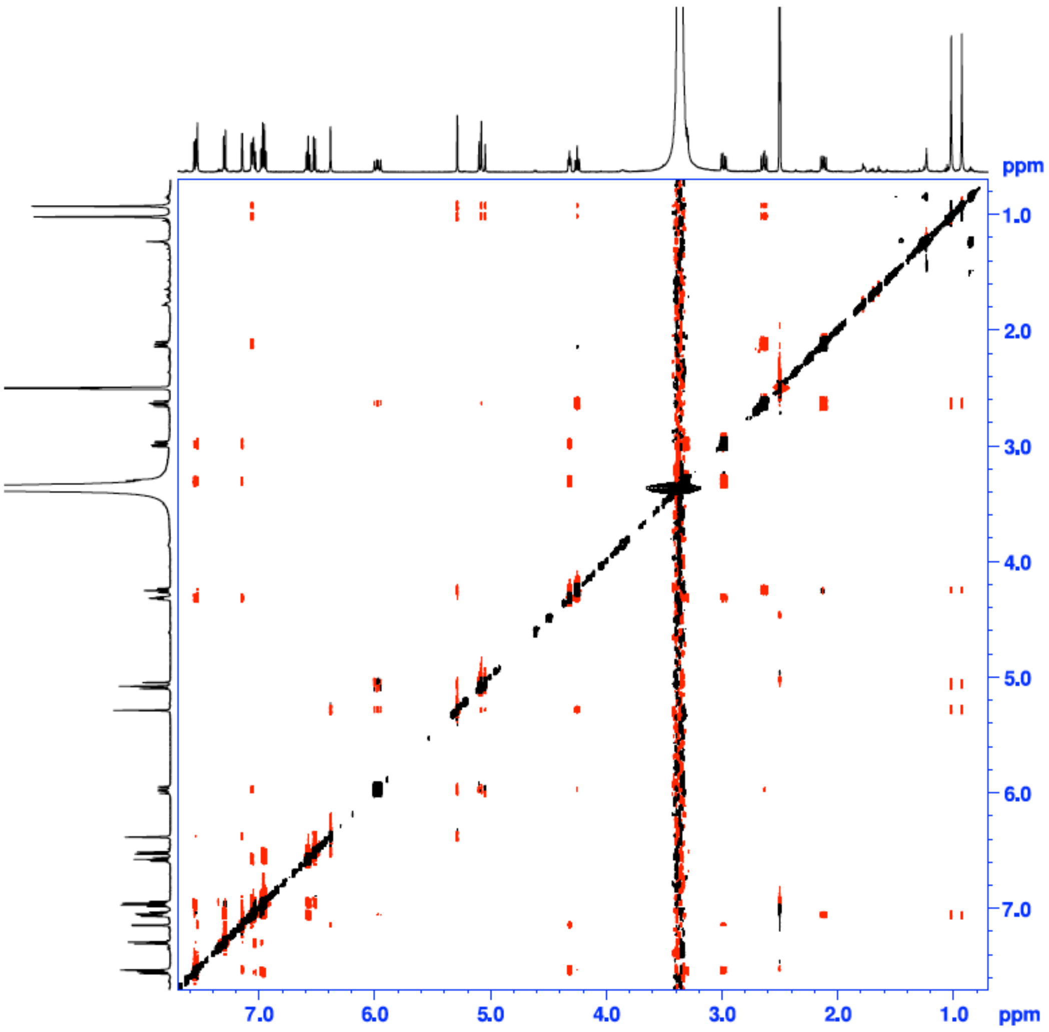


**Supplementary Figure S34**. 2D $^{1}$H-$^{1}$H ROESY spectrum of compound **9** in DMSO-*d6*.

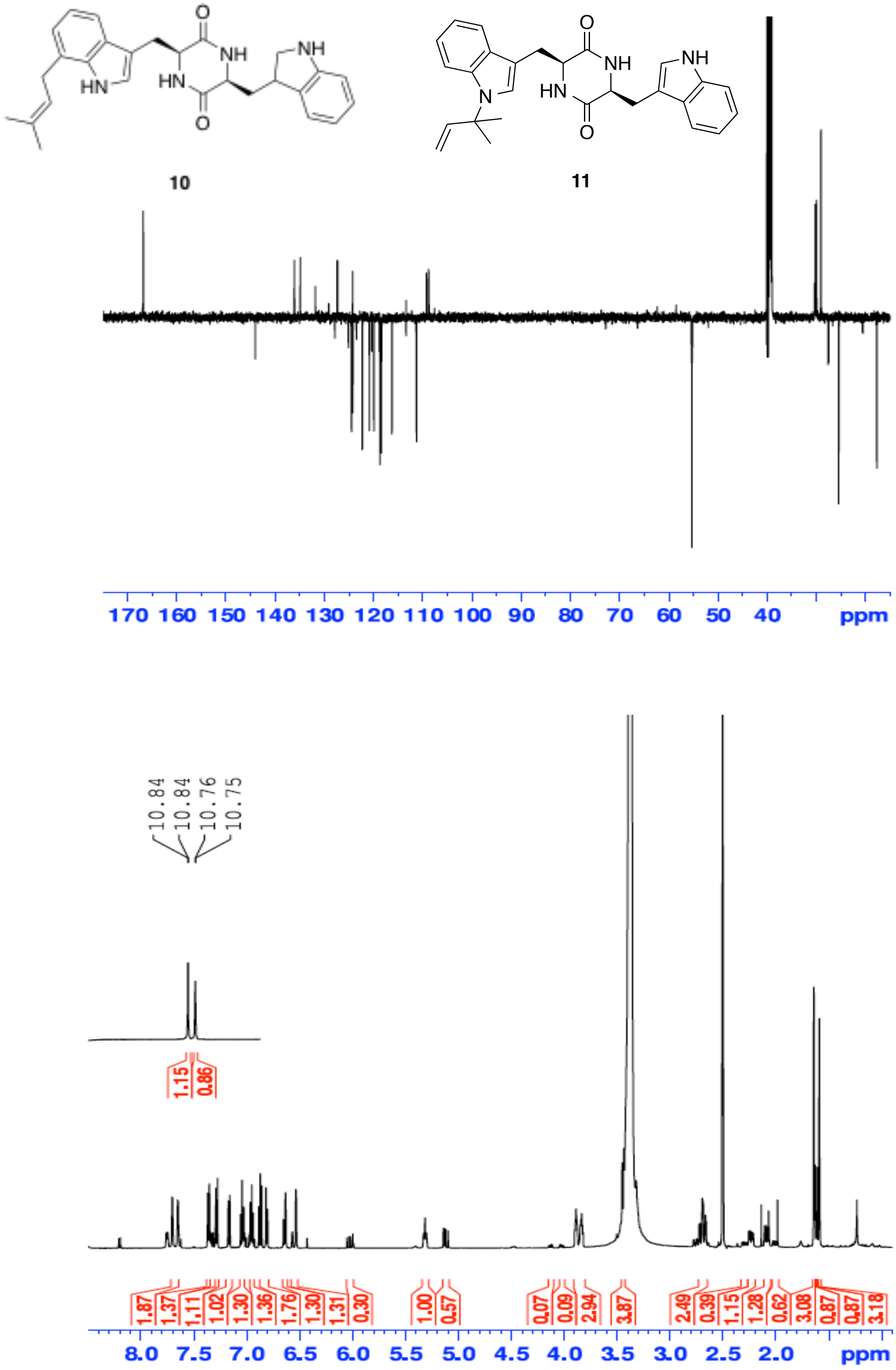


**Supplementary Figure S35**. 1D $^{13}C$ DEPTQ spectrum (upper panel) and 1D $^{1}H$ spectrum (lower panel) of compounds **10** and **11** in DMSO-$d_6$.

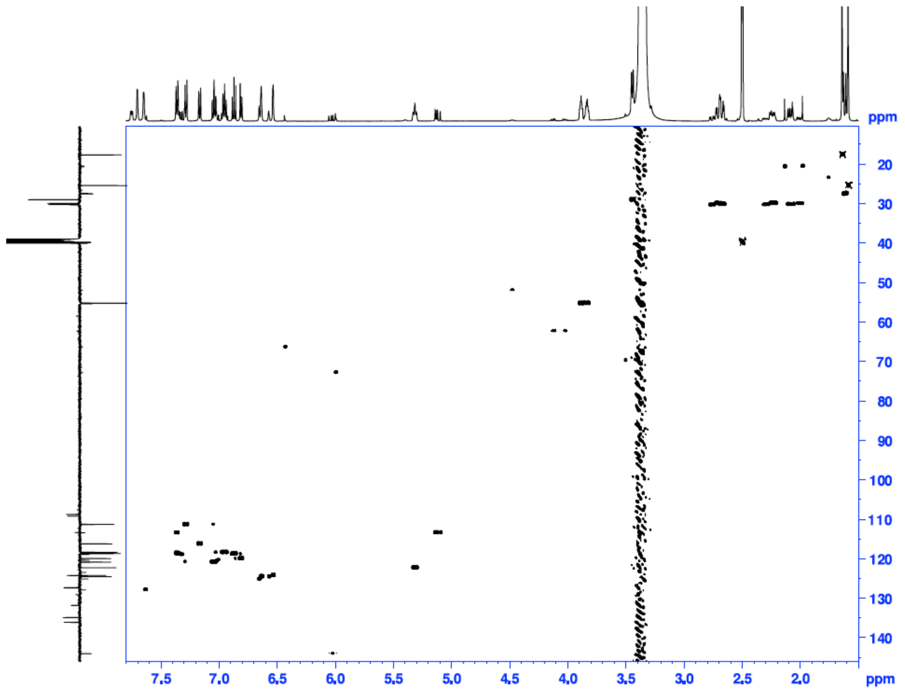


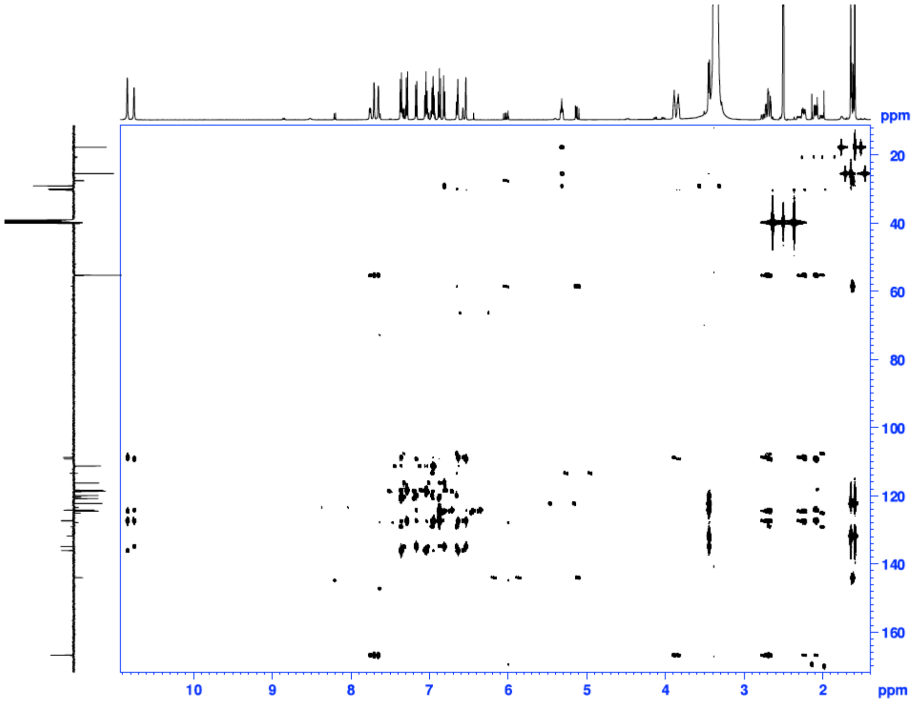


**Supplementary Figure S36**. 2D $^{13}C$-$^{1}H$ HSQC spectrum (upper panel) and 2D $^{13}C$-$^{1}H$ HMBC spectrum (lower panel) of compound **10** and **11** in DMSO-*d6*.

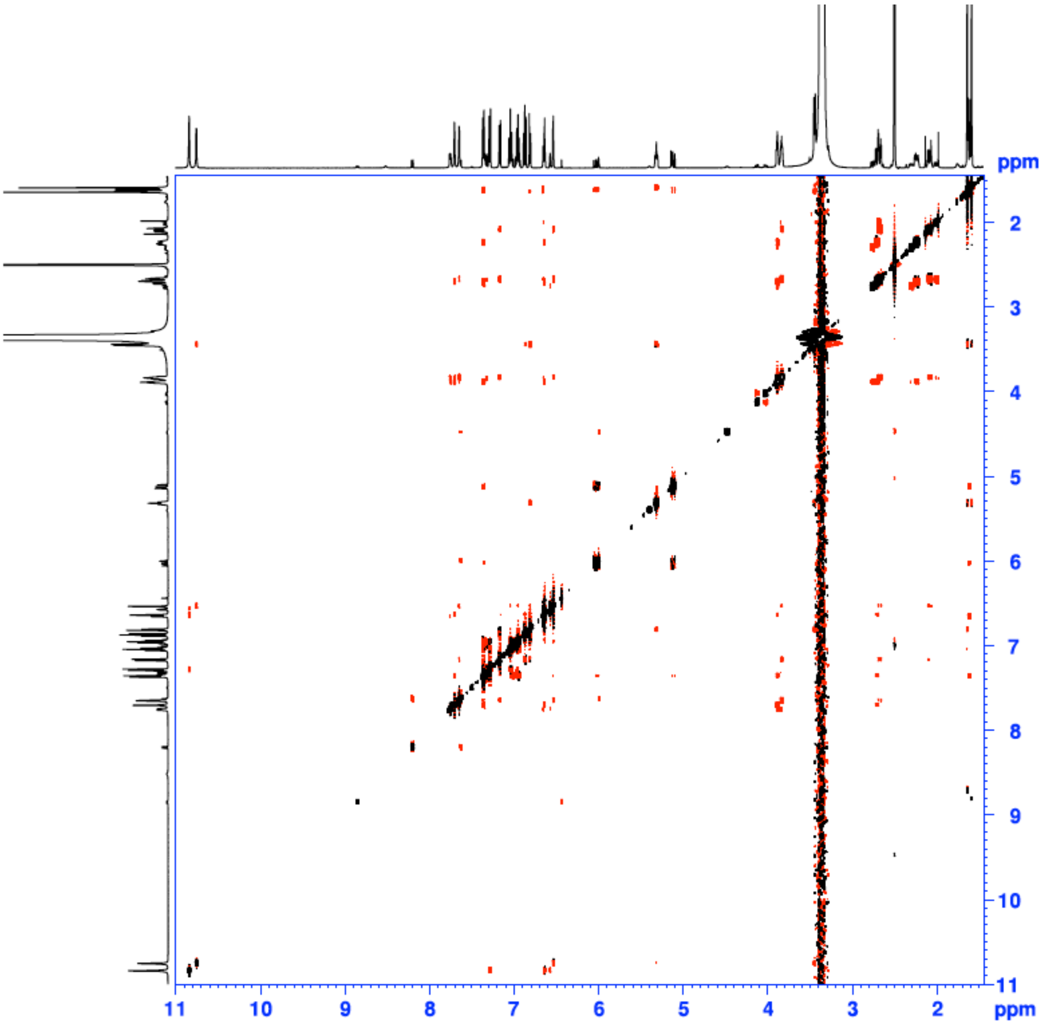


**Supplementary Figure S37**. 2D $^{1}H$-$^{1}H$ ROESY spectrum of compound **10** and **11** in DMSO-*d6*.
